
JOURNAL INFORMATION
==============================
NLM Title Abbreviation: JBMR Plus
Journal ID: JBMR Plus
NLM Journal ID: 101707013
Journal ID: jbmrplus

JBMR Plus

EISSN: 2473-4039
Publisher: Oxford University Press

ARTICLE INFORMATION
==============================
PMCID: PMC12274970
DOI: 10.1093/jbmrpl/ziaf122
Article ID: ziaf122
Article version: 1
Subjects: Abstracts, AcademicSubjects/MED00010, AcademicSubjects/MED00160, AcademicSubjects/MED00250

Abstracts of the Bone Research Society Annual Meeting 2025

Publication date: 2025 Aug
Electronic publication date: 2025 Jul 19
Volume: 9
Issue: Suppl 3
First page: iii1
Last page: iii155
Copyright: © The Author(s) 2025. Published by Oxford University Press on behalf of the American Society for Bone and Mineral Research.
Copyright year: 2025
License: This is an Open Access article distributed under the terms of the Creative Commons Attribution License (https://creativecommons.org/licenses/by/4.0/), which permits unrestricted reuse, distribution, and reproduction in any medium, provided the original work is properly cited.
License URL: https://creativecommons.org/licenses/by/4.0/

Page count: 1

==============================

Bone Research Society
ANNUAL MEETING 2025
25-27 JUNE 2025 | EDINBURGH, SCOTLAND
75 YEARS
75th Anniversary of BRS

### Book of Abstracts

### Edinburgh, UK

### Wednesday 25 – Friday 27 June 2025

### Invited Speaker Talk Summaries

*(where provided by the speaker)*

**INCLUDING**
**AHP and Osteoporosis Session**
**Renal Bone Workshop**
**Clinical Ageing Workshop**

### Oral Communications

### Printed Poster Abstracts

### Overview of the BRS Annual Meeting 2025

The Bone Research Society (BRS), formerly the Bone and Tooth Society, was founded in 1950. The BRS is one of the largest national scientific societies in Europe dedicated to clinical and basic research into mineralised tissues and is the oldest such society in the world. Meetings are held annually, attracting a wide audience from throughout the UK and beyond. The presentations are traditionally balanced between clinical and laboratory studies. The participation of young scientists and clinicians is actively encouraged.

We were delighted to welcome over 160 delegates, faculty and sponsors to our in-person meeting in Edinburgh, which was held at John McIntyre Conference Centre (JMCC), part of the University of Edinburgh campus from Wednesday 25 to Friday 27 June 2025. This year we were also celebrating the 75th Anniversary of the Bone Research Society.

There were two days (Thursday and Friday) with a full programme of invited speakers, oral communications, printed posters and three satellite symposia from our industry partners. We would like to thank our industry sponsors for their valuable support of our meeting.

The day before (Wednesday 25 June) we held three workshops; this year included was a specialised Image Workshop at the Roslin Institute site along with a new AHP and Osteoporosis Session at the JMCC. These were followed by the popular Early Career Researchers Workshop and networking session on the Wednesday evening at the JMCC.

Topics covered in the main programme were:

- Emerging Therapeutics in Osteoporosis
- Cancer and Bone
- Renal Bone
- Rare Bone Disease
- Biomineralisation: Initiation and Clinical Consequences
- The Musculoskeletal System in the Context of Multimorbidity
- Therapeutics: Engineering from Cells to Tissues
- The Aging Musculoskeletal System – Challenges at Home and Worldwide
- Cellular Communications in Bone Microenvironment

We were delighted to have excellent submissions from our authors which resulted in 17 being selected for Oral Communication presentations, 26 more for Quick Fire Poster Pitches and we had 75 printed posters on display.

***BRS 2025 Local Organising Committee***

Dr Louise Stephen (Chair)
Dr Lucie Bourne
Dr Beth Curtis
Ms Charlotte Clews
Professor Colin Farquharson
Dr Barbara Hauser
Dr Erika Kague
Dr Jennifer Paxton
Ms Aine Pears
Mr Worachet Promruk
Dr Clare Shere
Ms Rachel Wade

### Invited Speaker Talk Summaries

### AHP and Osteoporosis Session

### AHP1

### New Developments in FLS

Professor Muhammad K Javaid

University of Oxford, Oxford, UK

**Abstract**

The purpose of a Fracture Liaison Service (FLS) is to reduce fractures within available resources. Initially, FLSs were designed as one-stop shops for identifying patients from trauma wards and clinics for assessment and referring to primary care for therapy initiation. Over 25 years, our understanding of secondary fracture prevention has evolved dramatically. Not all fracture patients have the same refracture risk; specific sites like the spine, hip, humerus, wrist, and pelvis indicate higher risk. The timing of refracture is now seen as an imminent risk, especially within the first two years. Improved risk precision has identified higher-risk patients for whom anabolic therapies are clinically and cost effective as first line agents. Osteoporosis management is now supported by digital solutions, Quality Improvement and AI. The economic rationale for prioritising FLSs is now well-established, driving systematic secondary fracture prevention initiatives locally, regionally and nationally.

### AHP2

### Automated FLS with AI implementation

Dr Maria Talla

Queen Elizabeth University Hospital, Glasgow, UK

### Abstract

The risk of a second fragility fracture is highest within the first 12-24 months following an index fracture. Secondary prevention of osteoporotic fractures requires an effective Fracture Liaison Service (FLS).

We designed and implemented an innovative digital solution to facilitate early identification of fragility fractures. A text-based algorithm is deployed into the CRIS Radiology Information System to identify radiology reports which meet pre-specified criteria for FLS. This generates a list of unique patient identifiers which are then linked with biochemistry and radiology data from the Scottish Care Information (SCI) Store, alongside prescribing and bone densitometry data from the Safe Haven. This information displayed on a clinician dashboard, and is assessed and actioned daily, generating an individualised care plan for each patient which may include a recommendation for treatment, bone densitometry, biochemical investigations, or further assessment at the clinic.

Implementation of this system has resulted in a significant reduction in time to identification of vertebral fractures, an increase in the identification of patients with fragility (in particular vertebral) fractures and a reduction in our administrative workload. There has been an increase in the number of patients identified and treated with the same clinical resource, and we are able to take a targeted approach to prioritise patients at very high fracture risk for bone densitometry and assessment.

### AHP3

### Management of patients at very high fracture risk: from identification to anabolics

Professor Nicholas Harvey

University of Southampton, Southampton, UK

### Abstract

Osteoporosis and the management of high fracture risk have evolved enormously in recent decades. We have an established densitometric definition for osteoporosis, which has been the bedrock of the field's development, and generation of effective therapies. Furthermore, we have effective means to identify patients and stratify risk. Whereas guidelines have historically focused on identification of individuals for treatment (as opposed to no treatment), the advent of anabolic therapies, with their greater magnitude and speed of efficacy, has generated opportunities to stratify treatment according to baseline fracture risk. The approach was set out by ESCEO and IOF, with FRAX® as the mechanism for risk assessment, and adopted in many national guidelines, for example UK NOGG. Whilst in the ideal world, the most effective therapies would be used for every patient, most healthcare systems are resource constrained. Targeting the most effective therapies to patients at highest risk therefore makes best use of limited resources. In this presentation, I will review the science and implementation of stratified approaches to anti-osteoporosis treatment predicated on baseline fracture risk, and demonstrate the approach in practice using the NOGG pathway.

### AHP5

### Current approaches to osteoporosis assessment and treatment in young adults

Professor Jennie Walsh

University of Sheffield, Sheffield, UK

### Abstract

Causes of bone fragility in young people include malabsorption or poor nutrition, inflammatory disease and steroid treatment. Less common are postpartum osteoporosis and inherited disorders of bone metabolism.

Look for clinical features of osteogenesis imperfecta or hypermobility syndromes and endocrinopathies.

Tests for underlying causes of bone fragility include calcium, phosphate, alkaline phosphatase, vitamin D, FBC, ESR, coeliac antibodies, TSH. Bone turnover markers should be interpreted carefully before peak bone mass, because bone turnover is still naturally high.

In general, absolute fracture risk in young people is low, even in the context of low bone density. Management should begin with treatment of underlying causes where possible, and lifestyle modification.

The evidence base for the pharmacological treatment of young people is quite limited, and should be reserved for people at high current fracture risk

In women with premature ovarian failure, most clinicians would recommend oestrogen replacement until the usual age of menopause. There have been several clinical trials in anorexia nervosa, with some positive results from transdermal oestrogen and bisphosphonates. In glucocorticoid-induced osteoporosis there is some evidence for the use of bisphosphonates or teriparatide in young women.

It is important to consider potential pregnancies when treating women of child-bearing age, and there are case reports of congenital malformations and neonatal hypocalcaemia in association with bisphosphonates during pregnancy. Bisphosphonates which may have a quicker offset of action may be preferable in young women. Patients should be informed if use of osteoporosis drugs is outside the licence.

### Renal Bone Workshop

### Renal1

### Bone fragility in CKD - a paradigm shift

Professor Pieter Evenepoel

University Hospitals, Leuven, Belgium

### Abstract

Patients with CKD experience a multi-fold increased fracture risk compared to age and sex matched controls and the risk of mortality following a hip fracture is substantially higher. Both an increased fall risk and compromised bone strength contribute to the increased fracture risk in patients with CKD. Bone fragility in CKD is a composite of primary osteoporosis, accumulation of traditional and uraemia-related risk factors, assaults brought on by systemic disease, and detrimental effects of drugs (Jorgensen, David et al. 2021). For many years, osteoporosis and CKD-associated bone disorders, also referred to as renal osteodystrophy (ROD), were considered separate entities and mutually exclusive diagnoses. Consequently, nephrologists often deferred from osteoporosis management, while bone experts avoided patients with CKD-MBD. Participants of the 2023 Madrid CKD-MBD KDIGO Controversies Conference suggested that the term ROD may represent a roadblock to managing fracture risk as it fosters an overly PTH- and calcium–phosphate-centric approach to bone disease management. Since therapies focusing on these pathogenic drivers failed to meet expectations, conference participants argued that treatment of renal bone disease should be re-directed to the bone itself and that a change in terminology might facilitate this paradigm shift (Ketteler, Evenepoel et al. 2025). The term CKD-associated osteoporosis was introduced to acknowledge and emphasize that the diagnostic work up and therapy of CKD-associated osteoporosis are similar to what is proposed for other osteoporosis conditions, but need to be individualised with knowledge of CKD (Laurent, Dupont et al. 2025). A recent European consensus report aimed to foster a cohesive approach to the diagnosis and management of CKD-associated osteoporosis in patients with CKD G4-G5D (Evenepoel, Cunningham et al. 2021), to replace prevailing nihilism (renalism).

References

Evenepoel, P., J. Cunningham, S. Ferrari, M. Haarhaus, M. K. Javaid, M.-H. Lafage-Proust, D. Prieto-Alhambra, P. U. Torres, J. Cannata-Andia, a. i. o. t. C. K. D. M. B. D. w. g. o. t. E. R. A. E. European Renal Osteodystrophy workgroup, A. the committee of Scientific, I. O. F. National Societies of the, M. Vervloet, S. Mazzaferro, P. D'Haese, J. Bacchetta, A. Ferreira, S. Salam and G. Spasovski (2021). "European Consensus Statement on the diagnosis and management of osteoporosis in chronic kidney disease stages G4–G5D." Nephrology Dialysis Transplantation 36(1): 42-59.

Jorgensen, H. S., K. David, S. Salam and P. Evenepoel (2021). "Traditional and Non-traditional Risk Factors for Osteoporosis in CKD." Calcif. Tissue Int 108(4): 496-511.

Ketteler, M., P. Evenepoel, R. M. Holden, T. Isakova, H. S. Jorgensen, H. Komaba, T. L. Nickolas, S. Sinha, M. G. Vervloet, M. Cheung, J. M. King, M. E. Grams, M. Jadoul, R. M. A. Moyses and P. Conference (2025). "Chronic kidney disease-mineral and bone disorder: conclusions from a Kidney Disease: Improving Global Outcomes (KDIGO) Controversies Conference." Kidney Int 107(3): 405-423.

Laurent, M. R., J. Dupont, W. Lemahieu, S. Jamar, B. Mellaerts, M. Dejaeger, E. Gielen and P. Evenepoel (2025). "Treatment of Osteoporosis in Patients with Chronic Kidney Disease." Curr Osteoporos Rep 23(1): 26.

### Renal2

### The pathophysiology of bone loss and bone marrow adipose tissue expansion in experimental CKD

Professor Colin Farquharson

University of Edinburgh, Edinburgh, UK

### Abstract

Chronic kidney disease-mineral bone disorder (CKD-MBD) is the irreversible loss of kidney function leading to severe health issues including renal osteodystrophy (ROD) and bone marrow adipose tissue (BMAT) accumulation. Their underlying mechanisms remain unclear and, in this presentation, I will report data from pre-clinical models of CKD in an attempt to explain their pathophysiology. In CKD-MBD mice, the expression of mitophagy regulators was inconsistent with functional mitophagy, and in mito-QC reporter mice, there was a two- to three-fold increase in osteocyte mitolysosomes. Cultured osteoblasts with uremic toxins revealed increased mitolysosome number, distorted mitochondria morphology, decreased membrane potential and oxidative phosphorylation, and increased oxygen-free radical production. These effects were reversible by rapamycin. A causal link between uremic toxins and the development of mitochondrial abnormalities and ROD was established by showing that a mitochondria-targeted antioxidant (MitoQ) and the charcoal adsorbent AST-120 were able to mitigate the uremic toxin-induced mitochondrial changes and improve bone health. In further studies, bone loss, and adipocyte accumulation increased with disease duration. Moreover, during the early stages of CKD-MBD progression, bone marrow stromal cells from CKD-MBD mice had enhanced adipogenic potential but the proportions of osteoblastic and adipogenic precursor cells within the bone marrow were unchanged. In conclusion, our studies suggest that impaired clearance of damaged osteocyte mitochondria may contribute to the ROD phenotype and that BMAT expansion is not driven by altered precursor cell differentiation. Together, these findings may help to inform on the clinical management of CKD-MBD.

### Clinical Ageing Workshop

### CAW2

### The global problem of musculoskeletal aging: what are our knowledge gaps?

Professor Kate Ward

University of Southampton, Southampton, UK

**Abstract**

In low- and middle-income countries, there are already more than 1 billion people aged over 60 years. These older people spend longer living with disability and dependence than their peers in high-income countries, leading to individual, family, societal and healthcare burdens in some of the most resource-poor countries. Life expectancy is rising more rapidly in Africa than any other continent globally. This inevitable rise in older people will double the prevalence of osteoporosis and fragility fractures, sarcopenia, associated comorbidities, and increase associated morbidity and mortality. Rapid urbanisation, HIV, multimorbidity, malnutrition, changing physical activity patterns and climate change will contribute further to this rise. There has never been a timelier opportunity to act to prevent and address this challenge to older persons healthspan. Without action, health inequities will continue to grow for older persons in Africa. This talk will outline the population and health system level challenges that are being faced, drawing on current evidence, identifying gaps and potential implementable solutions.

### S1.2

### New signals in bone marrow: neutrophil progenitors inhibit osteoclasts

Professor Natalie Sims

St Vincent's Institute of Medical Research, Melbourne, Australia

**Abstract**

In inflammation, circulating neutrophils indirectly damage the skeleton by inducing formation of bone-resorbing osteoclasts. However, neutrophil progenitors in marrow have no known physiological function. We recently suggested a bone-protective role for marrow-residing neutrophils when we observed a profound defect in bone structure in mice with neutropenia due to Granulocyte Colony Stimulating Factor (G-CSF) deletion coupled with STAT3 hyperactivation in bone cells. We tested the existence of this protective effect by manipulating neutrophil progenitors in bone marrow by Ly6G antibody (aLy6G) treatment, often termed "neutrophil depletion". Two protocols revealed an inverse relationship between marrow neutrophil progenitors and osteoclasts. Two weeks of aLy6G treatment increased marrow immature neutrophils and halved osteoclast mRNA markers in cortical bone. In contrast, coupling six weeks of aLy6G with anti-rat IgG2a to maintain antigenicity halved the number of marrow pre-neutrophils. This doubled trabecular osteoclast surface, halved trabecular bone mass, and significantly reduced high density bone mass both in control mice, and in mice with bone-specific STAT3 hyperactivation. In culture, isolated pre-neutrophils dose-dependently inhibited osteoclastogenesis independent of direct contact. Proteomics analysis, coupled with single cell sequencing data showed that multiple previously identified osteoclast inhibitors are produced by neutrophil progenitors. We conclude that neutrophil progenitors directly inhibit osteoclast formation by releasing soluble factors. This identifies a new inhibitory pathway by which hematopoietic cells in marrow protect bone structure, which could be exploited for future osteoporosis therapies.

### S2.1

### Metabolism in the tumour-bone microenvironment

Professor Claire Edwards

University of Oxford, Oxford, UK

### Abstract

The bone marrow is a favoured site for a number of cancers, including the haematological malignancy multiple myeloma and metastasis from solid tumours such as breast and prostate cancer. This specialized microenvironment is highly supportive, not only for tumour growth and survival but also for the development of the associated destructive disease. The reciprocal interactions between tumour cells and bone cells, such as osteoclasts, osteoblasts, stromal cells and bone marrow adipocytes are key to driving rapid disease progression. Metabolic reprogramming is recognised as a hallmark of cancer. Furthermore, evidence is increasing for the importance of changing energy requirements within bone homeostasis. Interactions with bone cells alters metabolism within tumour cells, driving metabolic plasticity and leading to changes in tumour cell survival, senescence and dormancy. Conversely, tumour cells can impact the metabolic activity of bone cells, likely contributing to cancer-induced bone disease. As our understanding of such changing energy requirements increases, so does our appreciation for the potential for metabolic targeting to disrupt the complex crosstalk within the tumour bone microenvironment and ultimately reduce tumour burden and/or bone disease.

### S3.1

### New advances in Paget's Disease of bone

Professor Stuart Ralston

University of Edinburgh, Edinburgh, UK

### Abstract

Paget's disease of bone (PDB) is characterised by focal abnormalities in bone turnover driven by increased osteoclast activity coupled to dysregulated and disorganised bone formation. Genetics play an important role in PDB but environmental factors also contribute reflected by the fact that disease prevalence has reduced in many countries over the past five decades. Suggested environmental triggers include exposure to infections, changes in diet, mechanical loading of affected bones, changes in exposure to farm animals and exposure to toxins and pollutants. Genome sequencing and association studies have identified thirteen loci with robust evidence of association with PDB implicating genes that regulate osteoclastogenesis and the ubiquitin proteasome system. The diagnosis of PDB can usually be made by clinical features and typical x-ray changes but radionuclide bone scan examination is the preferred method to delineate the full extent of the disease. Medical management if PDB is based on the use of osteoclast inhibitors of which bisphosphonates are the most widely used. Of the bisphosphonates, zoledronic acid is the current treatment of choice. Although pain is the most common symptom, recent studies have shown that osteoarthritis is the most common cause of pain in PDB emphasising the importance of delineating the cause of pain so that treatment can be tailored appropriately to the individual patient. Genetic testing for pathogenic variants in *SQSTM1* coupled with prophylactic therapy with zoledronic acid in carriers of pathogenic variants has recently been shown to be effective in favourably altering the natural history of the disease.

### S3.2

### Modelling osteogenesis imperfecta using alternative animal models

Dr Antonella Forlino

University of Pavia, Pavia, Italy

### Abstract

Modelling osteogenesis imperfecta (OI) using alternative animal with respect the traditional murine models has proven invaluable in testing potential therapies but also in understanding disease mechanisms and. Among these models, the zebrafish (D. rerio) stands out due to its genetic tractability, rapid development, and optical transparency. Zebrafish possess a well-conserved skeletal system, and both the ossification and the types of skeletal cells are highly conserved with mammals. This makes them particularly suited for reproducing bone disorders such as OI. Zebrafish models have been successfully used to replicate both dominant and recessive forms of OI. Mutations in col1a1a and col1a2, the zebrafish orthologs of human COL1A1 and COL1A2, recapitulate features of dominant OI, including bone fragility and deformity. Meanwhile, recessive forms have been modelled by targeting genes involved in collagen modification and processing, such as bmp1, plod2, p3h1, crtap and tmem38b. These models reproduce key pathological features, including bone deformity, abnormal bone matrix, and often spontaneous fractures. In addition to offering insights into disease etiology, zebrafish models are also well suited for high-throughput drug screening. Their external development and transparency allow real-time imaging of skeletal development and pathology. Overall, zebrafish provide a versatile and powerful system for studying the full spectrum of OI pathologies. An overview of our most recent findings will be provided.

### S4.1

### New techniques in imaging and studying mineralisation

Professor Alexandra Porter

Imperial College London, London, UK

### Abstract

Bone is a hierarchically structured tissue composed of both organic components—such as collagen and non-collagenous proteins—and inorganic components, primarily hydroxyapatite (HAP). Despite significant progress, many key aspects of biomineralisation remain poorly understood, including cell-mediated mineral deposition, the role of trace elements, and the precise arrangement and structure of minerals within the collagen matrix. This talk will discuss the use of advanced cryogenic and advanced nanoscale chemical electron microscopy techniques to shed light on different mineralisation processes in bone and cartilage (1-4).

Cartilage mineralisation is central for maintaining bone mechanical integrity and remodelling. However, the mechanism by which chondrocytes regulate this process remains poorly understood. I will describe how mineralizing chondrocytes accumulate calcium phosphate within mitochondria, and in intracellular and extracellular matrix vesicles (MVs) at the growth plate and subsequently utilize these structures to mineralise the collagen fibrils. [1].

Further, I will demonstrate how these electron microscopy techniques can be integrated with x-ray scattering and atom probe microscopy techniques, to study mineralisation in both healthy and pathological bone [3-4]. I will highlight the role of osteopontin—a non-collagenous protein—in determining the structure and organisation of both the organic and inorganic phases of bone [3].

### References

1. Boonrungsiman S. et al. PNAS 109, 14170-14175 (2012)
2. Boonrungsiman S, Acta Biomaterialia 191:149-157 (2025)
3. Depalle, B, Acta Biomaterialia 120, 194–202 (2021)
4. Schwarz TM , Acta Biomaterialia, 319-333 (2025)

### S4.2

### Clinical manifestations of hypophosphatasia in adults

Dr Katie Moss

St George's Hospital, London, UK

### Abstract

Hypophosphatasia (HPP) is an inherited metabolic disorder caused by variants in the ALPL gene leading to reduced activity of tissue non-specific alkaline phosphatase (TNAP).

Classic symptoms of HPP presenting in adults include pseudo fractures and long bone pain due to under mineralised bone and a history of premature loss of deciduous teeth.

Over the last 2 decades, there has been increasing recognition that people with HPP can experience a range of other symptoms including widespread musculoskeletal pain, gait disturbance, weakness and fatigue, as well as neuropsychiatric and gastrointestinal problems.

In my practice of more than 50 patients with HPP, some have had significant musculoskeletal symptoms (in the absence of fracture) leading to reduced mobility and quality of life. Widespread musculoskeletal pain is common, particularly affecting women. My clinical impression is that this can be due to a combination of long bone pain, joint pain due to early onset calcium pyrophosphate deposition disease (CPPD) or early onset osteoarthritis, and proximal muscle weakness is also a contributary factor. CPPD is usually diagnosed due to the characteristic pattern of joints affected by arthritis, and the description of the arthritis flare ups. Although it can sometimes be challenging to confirm CPPD with currently available investigations. Further research is needed to characterise these non-fracture aspects of HPP.

In my talk I will discuss the clinical phenotype of some of the patients with HPP in my clinic as well as what the literature has taught us so far.

### S5.1

### Subchondral bone a source of osteoarthritis pain

Dr Daniel McWilliams

University of Nottingham, Nottingham, UK

### Abstract

The joint is a complex structure with many different tissues and cell types. Chronic pain is a large burden on individuals and societies, especially as populations age. Osteoarthritis (OA) is a common chronic joint disorder, and musculoskeletal pain is the major symptom.

This talk will focus on evidence for the subchondral bone as source of OA pain. Other tissue types have been linked to OA pain, including, synovium and cartilage. Additionally, extra-articular factors such as central pain sensitivity, are critical in shaping the pain experience. However, this talk will mostly focus on the subchondral bone.

Neoinnervation of the cartilage from the subchondral bone, through the osteochondral junction, is a strong candidate source of OA pain; and sensory nerves can be found invading the cartilage alongside vessels. Nerve growth factor, a neurotrophic, and sensitising, molecule is also found within the tissues in the channels entering the cartilage. Bone marrow lesions (BMLs) in areas of weight-bearing are associated with pain on movement. Studies have characterised the cellular content of BMLs, which are known to contain nerves, and might provide therapeutic targets for pain relief. Osteoclast actions are associated with OA pain, suggesting a role for bone remodelling. Recent studies have also highlighted the roles of immune cell infiltration within the dorsal root ganglia, opening avenues of research into peripheral mechanisms of OA pain that are localised remotely from the joint.

### S5.2

### Multimorbidity and musculoskeletal health in later life

Professor Elaine Dennison

MRC Lifecourse Epidemiology Centre, Southampton, UK

### Abstract

Multi-morbidity refers to the coexistence of two or more chronic disease conditions within an individual. Prevalence estimates for multimorbidity range from 20 to 30% in “all age” populations and may be as high as 55–98% in older populations. Prevalence estimates are expected to rise further due to ageing populations, and medical advances.

Multimorbidity is associated with musculoskeletal conditions of aging, specifically osteoporosis, osteoarthritis and sarcopenia. We have studied this issue in the Hertfordshire Cohort Study, a UK community-based sample of men and women born in 1931-9. Among 2997 individuals aged 59-73 at baseline a greater number of systems medicated was related to increased risk of falls ($P < 0.001$) and lower limb joint replacements ($P < 0.003$) over about 10 years of follow-up. More systems medicated was only related to increased risk of fracture among women however. When we studied associations between presence of sarcopenia or osteoporosis in a very well characterised subsample of the same group, sarcopenia and osteoporosis were both individually associated with having three or more comorbidities (OR 4.71, 95% CI 1.50, 14.76; $P = 0.008$ and OR 2.86, 95% CI 1.32, 6.22; $P = 0.008$, respectively). Results such as these highlight the need to consider associations between multimorbidity and musculoskeletal health in later life, as demographic change and health care advances will make this an increasingly common problem.

### S6.2

### Therapeutic biomaterials for osteoporotic bone regeneration

Dr Ciara Murphy

RCSI University of Medicine and Health Sciences, Dublin, Ireland

### Abstract

Osteoporosis is the most widespread metabolic bone disorder globally, contributing to a fracture every three seconds. Osteoporotic vertebral fractures (OVFs) are its most frequent complication, substantially increasing the risk of subsequent fragility fractures, chronic pain, and diminished quality of life. Current clinical interventions, such as vertebroplasty and kyphoplasty, involve injection of permanent bone cements that provide mechanical stabilisation but do not support bone regeneration. These treatments are associated with significant limitations, including cement leakage and an increased risk of adjacent vertebral fractures due to mechanical mismatch and lack of biological integration.

Our research addresses this clinical gap through the development of injectable biomaterials based on natural polymers, tailored to support both mechanical reinforcement and biological repair of osteoporotic bone. By incorporating nanoscale reinforcements and therapeutic ions such as strontium and lanthanides, we aim to restore the mechanical properties of compromised vertebrae while modulating pathological bone remodelling to enhance regeneration. These multifunctional materials are designed to promote osteogenesis and inhibit excessive bone resorption.

To evaluate structural performance, we employ finite element analysis (FEA) modelling of osteoporotic spinal segments, enabling simulation of load transfer and prediction of secondary fracture risk. This computational approach informs the optimisation of biomaterial design for site-specific application and long-term biomechanical compatibility.

This presentation will outline our progress in developing therapeutic, bioactive, and mechanically competent natural polymer-based biomaterials for OVF repair—integrating material science, biological function, and computational modelling to advance regenerative treatment strategies for osteoporotic spine injuries.

### S7.2

### Primary cilia in limb growth, health and disease

Dr Angus Wann

University of Southampton, Southampton, UK

### Abstract

Having spent more than a century in the relative shadows of cell biology, primary cilia have been brought centre stage in the last 25 years. Their biological influence is now well-described. A subset of the human diseases associated with disruption of this biological influence, includes the skeletal ciliopathies. As a result, the singular primary cilium, assembled by the majority of cells of the skeleton, has now received significant attention.

In cartilage and bone, cilia are proposed to play critical roles transducing a spectrum of extracellular signals. Perhaps the best described example of this is their role in growth factor signalling, another is a role in shaping the cellular and tissue response to mechanical force.

In bone, cilia have long been described as mechanosensors, potentially using analogous mechanisms to those described in the developing node where primary cilia 'sense' directed fluid flow. Research in cartilage, including our own, implies cilia play highly context-dependent roles.

In this talk, focussing on our recent work in the post-natal mouse growth plate, I'll describe why we have spent considerable time analysing the direction cilia are orientated in a 3-dimensional space. I'll describe some of our recent *in vivo* data concerning cilia position and orientation in the growing limb and how this is inspiring *in vitro* approaches seeking to define mechanisms for ciliary influence in the development and health of skeletal tissues.

### Oral Communications

### OC1.1

### Modelled impact of artificial intelligence algorithm to improve diagnosis of vertebral fractures embedded in fracture liaison services depends optimised FLS performance and estimated date of vertebral fracture

Professor Rafael Pinedo-Villanueva, Dr Gianluca Fabiano, Dr Franz Clemeno, Professor Muhammad Javaid

University of Oxford, Oxford, UK

**Abstract**

Objective

To estimate the patient benefit and economic impact of integrating an artificial intelligence (AI)-enabled vertebral fracture (VF) identification algorithm into optimally-run FLSs under various scenarios

Material and Methods

The Nanox-AI HealthVCF algorithm was implemented into the radiology workstream of three UK NHS hospitals. The AI analysed existing CT scans and flagged those with potential moderate/severe fractures for local clinical confirmation. Patients with confirmed scans were referred to the local FLS for further assessment and management. Using a microsimulation model, the impact of the AI on patient outcomes and health and social care costs was estimated for a cohort of 1000 male and 1000 female patients with confirmed VF over five years. We used observed FLS performance metrics before and after AI implementation from the FLS-Database of England and Wales and inputs from clinical experts to populate the model comparing results under pre-AI observed FLS to AI optimised-FLS settings and setting the date of vertebral fracture to 5 years prior to index date of CT scan.

Results

In the base case model comparing observed FLS pre-AI, the optimised FLS post-AI prevented 110 fractures over 5 years in the cohort (Table). This translated to a 5 year saving in hospital costs of £280,419, rehabilitation costs of £243,531 and social care costs of £1,938,711. The expected FLS staff, laboratory, DXA costs were £325,011 and medication costs £2,081,528. Over 5 years this equated to a £51,339 overall *saving* with 102 QALY gain. The cohort became cost-saving by year 3. Resetting the date of VF to 5 years prior to the CT scan date, reduced the number of re-fractures to 22 with a net cost of £1,662,717 for the cohort.

Conclusion

Despite differences in age and higher mortality, adding AI to flag potential vertebral fractures can lead to both substantial reductions in subsequent fractures and in health and social care costs. These benefits are sensitive to the assumed date of vertebral fracture and further work is needed to establish the baseline re-fracture rate for this presentation of VF.

Expected subsequent number of fractures, patients with subsequent fractures at QALYs at 5 years for 1,000 women and 1,000 men with a sentinel spinal fracture for FLS with no AI, AI with observed FLS and AI with optimised FLS.

| | Comparators | | | | Impact (fractures avoided) | | | ATTRIBUTION |
|---|---|---|---|---|---|---|---|---|
| **HEALTH OUTCOMES** | **Current FLS (No AI)** | **AI + FLS_obs** | **AI + FLS_optim** | | **AI + optimal FLS** | **AI** | **Optimal FLS +** | **Prop. of fractures avoided** |
| **Subsequent fractures at 5 years** | | | | | | | | (AI / Opt_FLS) |
| Subsequent-fractures: hips | 284 | 282 | 254 | | 30 | **2** | **28** | 7% / 93% |
| Subsequent-fractures: spines | 231 | 229 | 195 | | 36 | **2** | **34** | 6% / 94% |
| Subsequent-fractures: other | 531 | 528 | 487 | | 44 | **3** | **41** | 7% / 93% |
| **Total re-fractures** | **1,046** | **1,039** | **936** | | **110** | **7** | **103** | 6% / 94% |

### OC1.2

### Real-World Efficacy Analysis of 12-Month Romosozumab Followed by 12-Month Antiresorptive Treatment Compared to 24-Month Teriparatide

Ms Elizabeth Orhadje[1], Dr Pollie Ravji[2], Ms Nikita Bendstrup-Charlton[3], Ms Kathryn Berg[1], Prof Stuart H Ralston[1,2], Dr Barbara Hauser[1,2]

[1]Centre for Genomic and Experimental Medicine, Institute of Genetics and Cancer, University of Edinburgh, Edinburgh, UK. [2]Rheumatology Department, NHS Lothian, Edinburgh, UK. [3]University of Edinburgh, Edinburgh, UK

### Abstract

**Background:** Romosozumab (ROMO) and Teriparatide (TPTD) are established anabolic osteoporosis treatments for patients at very high fracture risk, with proven efficacy in reducing vertebral fractures[1,2]. However, no direct comparisons exist to guide treatment selection.

**Purpose:** This study aimed to evaluate bone mineral density (BMD) responses at 12 and 24 months in patients treated with ROMO followed by antiresorptive treatment (ART) versus TPTD.

**Methods:** A retrospective single-centre analysis included patients treated with ROMO for 12 months followed by ART or TPTD for 24 months between February 2021 and September 2024. Data were extracted from electronic health records.

**Results:** The ROMO group (n=111) was older (73.1 ± 9.0 vs. 68.1 ± 8.1 years, p<.001) and had a lower BMI (23.4 ± 5.9 vs. 25.6 ± 5.4 kg/m², p<.001) than the TPTD group (n=107). More TPTD patients were treatment-naïve (65.1% vs. 45.8%, p=.006). Baseline lumbar spine BMD was comparable (T-score: -3.86 ± 1.0), but femoral neck BMD was lower in the ROMO group (-3.15 ± 0.68 vs. -2.75 ± 0.76, p<.001). Most patients had a 1-year scan, however, due to various reasons (frailty, death, clinical decision), only 41 ROMO patients and 68 TPTD patients had a 2-year DEXA scan.

ROMO resulted in greater lumbar spine BMD gains at 12 months (p=.004), but no differences were observed between groups at 24 months (Fig 1A). Femoral neck BMD gains were superior in the ROMO group at both time points (p=.034)(Fig 1B). Treatment-naïve patients demonstrated significantly greater increases in lumbar spine BMD at 24 months (16.9 ± 10.0% vs.10.6 ± 8.2%, p=.001) compared to pre-treated patients, regardless of the treatment type

**Conclusion:** ROMO patients had more severe osteoporosis at baseline. ROMO demonstrated faster lumbar spine BMD improvement but similar results to TPTD at 24 months. Femoral neck BMD gains were greater with ROMO. Prior antiresorptive therapy blunted lumbar spine BMD responses, highlighting the importance of treatment sequencing.

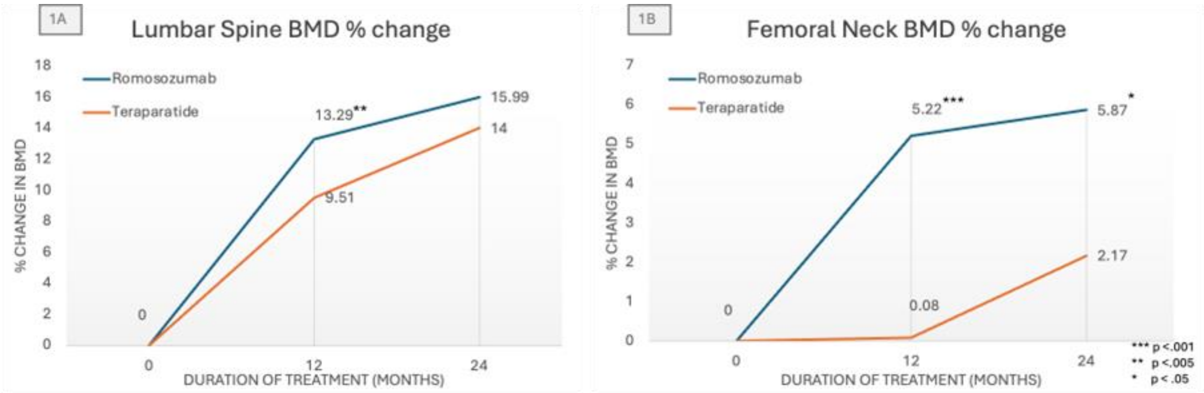

*Figure 1.*

*Lumbar spine and femoral neck BMD increase after 12 and 24 months.*

### OC2.1

### The adipokine resistin is increased in the myeloma-bone niche in vivo: a novel link between aging, obesity and myeloma progression

Danielle Whipp, Ms Katie Booth, Ms Magdalena Hutchins, PhD Claire Edwards, PhD Beatriz Gamez

University of Oxford, Oxford, UK

### Abstract

Ageing and obesity are associated with negative changes in bone homeostasis, including increased adiposity and a decrease in osteoblasts within the bone marrow (BM). Multiple myeloma (MM) is a malignancy of plasma cells which accumulate in the BM. Ageing and obesity are major risk factors for myeloma, however, the key mechanisms through which they alter the BM, promote myeloma progression, and might be targeted for patient benefit are poorly understood and remarkably under-studied.

We and others have shown that obesity promotes MM in humans and mice. Our recent data indicates a direct association between increased cholesterol and MM growth in vivo. The current study aimed to identify novel mechanisms underlying myeloma progression. Using an adipokine proteome profiler, we identified a significant increase in the adipokine resistin, in mice on a high cholesterol diet (vs normal diet), and myeloma-bearing mice (vs non-tumour) (>2-fold). Importantly, resistin was increased in BM plasma, but not in serum, suggesting a specificity for the bone microenvironment. Supporting this finding, in silico analysis of MM patients (GSE47552 dataset), showed increased resistin in the myeloma precursor condition MGUS (monoclonal gammopathy of undetermined significance), providing clinical evidence for a role in MM progression. In an in vitro cell panel representing the MM-marrow niche, resistin was predominantly secreted from BM adipocytes (BMAds), with BMAds expressing 1.5x105-fold more and secreting 50-fold more resistin than stromal and myeloma cells. Treatment of BMAds with cholesterol/LDL (low-density lipoprotein) increased adipogenic markers (1.5-fold, $p<0.05$), resistin expression (2-fold, $p<0.05$) and secretion (1.7-fold, $p<0.05$) vs control. Pre-osteoblasts (2T3) treated with LDL also show increased resistin expression (2-fold) suggesting cholesterol/LDL exposure corrupts bone cells to favour MM progression. Co-culture with either BMAds or ST2 stromal cells increased myeloma cell expression, while preliminary data showed recombinant resistin treatment promoted MM cell viability and migration (2-fold) and increased MM cell adhesion to pre-osteoblasts.

Furthermore, aged C57BL/6 mice (18-20 months) showed higher levels of resistin (1.5-fold) in BM plasma compared to young mice (8-14 weeks), and treatment of BM stromal cells with 500nM doxorubicin (to induce senescence, a characteristic of ageing BM) increased the expression of resistin (15-fold, $p<0.01$) compared to control.

We have identified resistin as not only elevated in myeloma, but also elevated in high cholesterol and ageing, with specificity for the BM. Taken together, our results suggest that resistin expression is increased in the BM niche and acts as a novel mediator of myeloma progression.

### OC2.2

### Feline oral squamous cell carcinoma cell lines derived factors stimulate osteoclastogenesis and bone resorption, facilitating bone invasion

Mr. Qaisar Tanveer, Dr. Lisa Pang, Prof. Colin Farquharson, Prof. Gurå Therese Bergkvist

The Roslin Institute, The Royal (Dick) School of Veterinary Studies, The University of Edinburgh, UK

### Abstract

Feline oral squamous cell carcinomas (FOSCC) are frequently diagnosed tumours and serve as a clinically relevant model for human head and neck cancers. Oral tumours account for about 10% of all tumours diagnosed in cats, with FOSCC accounting for approximately 70% to 80% of all feline oral tumours. These locally aggressive tumours exhibit bone invasion, characterised by excessive osteoclast-mediated bone resorption. Identifying biomarkers for bone invasion is crucial to improve prognostic strategies and clinical outcomes. Tumour secretomes offer a rich source of potential biomarkers that drive proliferation, metastasis, and invasion.

To investigate the osteolytic potential of FOSCC, we induced osteoclast formation and bone resorption from feline bone marrow precursors with conditioned media (CM) collected from *bone-invasive* and *bone non-invasive* FOSCC cell lines using co-culture system and *ex-vivo* explant model. Protein changes in FOSCC CM were analysed using single-shot label-free quantification (LFQ) proteomics on a timsTOF-HT mass spectrometer.

Conditioned media from *bone-invasive* FOSCC cell line in the presence of CSF and RANKL significantly increased osteoclast differentiation from bone marrow and osteoclast resorption activity compared to CM from *bone non-invasive* FOSCC cell line ($p<0.05$). Consistent with these results, co-culture of mouse calvaria with *bone-invasive* FOSCC cells resulted in a significant elevation of osteoclast numbers by day 7 compared to *bone non-invasive* FOSCC cells ($p<0.05$). Comparative proteomic analysis revealed 306 proteins uniquely secreted by *bone-invasive* cells, among which seven proteins (FGFR3, BMP2, NOTCH2, TFRC, PRXL2A, SFRP1, and IFNAR1) are directly implicated in osteoclast differentiation, and 43 are associated with bone biology, suggesting a complex interplay of signalling pathways driving bone invasion. While 2364 proteins were commonly expressed in both *bone-invasive* and *bone non-invasive* cell lines, 173 of these were differentially expressed proteins (DEPs). Among these DEPs were proteins such as FGF21, LTBP3, MECP2, LAMP2, IGFBP4 and HEXB which are associated with abnormal bone morphology. Furthermore, MST1, TMPRSS13, EPHB6, EPHA7, ITGβ1, PCDHB10, TRAP1 and CDH6 were identified as putatively novel secreted factors, warranting further investigation. Ingenuity Pathway Analysis predicted upstream regulators including TP53, CLpP, PTEN, ESR1, MAP4K4, TGFβ-1 and HNF4A which are known to drive proliferation, migration, and invasion in cancer, supporting the validity of our approach.

Together, these co-culture systems and proteomic analysis provide a robust approach for identifying the secreted factors within FOSCC cells conditioned media that potentially drive bone invasion.

### OC2.3

### High Cholesterol Diet Promotes Increased Tumour Burden, Bone Disease and Bortezomib Resistance in Multiple Myeloma

PhD Beatriz Gamez[1,2], MBiochem Danielle Whipp[1], PhD Srinivasa Rao[1,2], PhD Emma V. Morris[1], PhD Young Eun Park[1,2], PhD Zeynep Kaya[1], PhD Claire M. Edwards[1,2]

[1]University of Oxford, Oxford, UK. [2]Oxford Translational Myeloma Centre, Oxford, UK

### Abstract

Multiple myeloma (MM) is a B cell malignancy where plasma cells grow uncontrollably in the bone marrow (BM) leading to osteolytic bone lesions. Despite improvements in overall survival MM remains incurable in part due to drug resistance. We previously showed how diet-induced obesity induces a myeloma-like condition in mice however the exact contribution of cholesterol to myeloma progression is poorly understood.

To determine the effect of high cholesterol in vivo, a 2% cholesterol diet was used in C57Bl/KaLwRij mice prior to tumour inoculation. After 4 weeks mice had increased total serum cholesterol with high LDL (2-fold, $p<0.05$). Following inoculation with 5TGM1MM cells, mice on the cholesterol diet had increased BM tumour burden (2-fold, $p<0.01$), higher MM cell lipid content, increased osteolytic bone lesions ($p<0.01$) and a reduction in cortical bone density. No significant difference in tumour burden was detected when cholesterol treatment was halted at time of MM inoculation. A panel of MM cells were cultured with delipidated-FBS, with a significant reduction in viability ($p<0.001$). Rich cholesterol content lipoproteins (LDL) but not VLDL restored cell viability, implicating cholesterol in MM cell survival.

LDL addition under metabolic stress (no FBS) prior to treatment with the proteasome inhibitor bortezomib (bzb) induced resistance in a panel of MM cells. No effect was seen with other MM-drugs including other proteasome inhibitors. Whole BM containing 5TGM1-GFP from MM-bearing mice was isolated for ex vivo experiments, where LDL-mediated resistance to bzb was also seen ($p<0.001$). LDL-pretreated cells did not exhibit proteasome inhibition and were protected against bzb-induced apoptosis as seen by cPARP and MCL1 protein expression and cell cycle analysis. LDL-pretreated cells presented changes in membrane fluidity together with increased Cav-1 expression which might suggest modifications in the ability to incorporate bortezomib. RNA-seq demonstrated that LDL restored the gene signature of bortezomib-treated MM cells. Analysis of the MMRF-CoMMpass patient data demonstrated that HMGCR and HMGCS1 gene expression determines the first response probability only for patients with bzb-based therapies suggesting a key role for cholesterol in MM response.

Our results show that cholesterol has a direct pro-tumourigenic effect on MM pathogenesis increasing BM tumour burden and osteolytic lesions in vivo and bzb resistance ex vivo. Additionally, LDL protects MM cells from bortezomib, restoring the gene signature of bortezomib-treated MM cells and protecting against apoptosis. These results identify cholesterol as a mediator of myeloma pathogenesis and bzb resistance and provide new directions for dietary or pharmacological strategies.

**OC3.1**

Withdrawn

### OC3.2

### Progression of Multifocal Carpo-Tarsal Osteolysis in adulthood and potential response to 120mg denosumab 4 weekly

Dr Claudia Maushart[1], Dr Claire Holmes[2], Dr Paul Cook[2], Dr David Hunt[2], Professor Muhammad Javaid[1]

[1]University of Oxford, Oxford, UK. [2]University Hospital Southampton NHS Foundation Trust, Southampton, UK

### Abstract

Multicentric Carpo-Tarsal Osteolysis (MCTO) is a rare osteolysis disorder beginning in childhood caused by mutations in *MAFB*, which is essential for osteoclastogenesis and renal development. Pain management in MCTO presents a significant treatment challenge. We provide an update on a previously reported MCTO case (BRS annual meeting 2021), highlighting a potential response to denosumab.

A 27 year old white British woman was reviewed in the joint rare bone disease clinic. She was diagnosed at the age of 3 years due to a right distal radius fracture and carpal osteolysis/ Genetic testing confirmed heterozygosity for *p.Ser69Leu* mutation, inherited from her father, who had a similar phenotype and was initially treated as having an inflammatory arthritis and died from a pulmonary embolism.

In an attempt to arrest the osteolysis, the patient was treated with IV pamidronate (2005-2007, 20 months), calcitonin intranasally/sc (not tolerated), IV zoledronic acid (2008-2010, six doses), denosumab (from 2011: 40 mg every 6 months, later increased to 60 mg every 2 months, and subsequently monthly until June 2014). In 2014, the patient fell and sustained an atypical-like fracture of the right femur and the denosumab was switched to teriparatide for 18 months. Proteinuria remained mild and was managed with an ARB.

Despite these treatments, she had experienced severe osteolysis, including complete dissolution of the carpal bones and significant joint erosions (Figure). Given the progressive loss of upper limb function and pain, from August 2022, she was treated again with 120mg denosumab 4 weekly. During this time, there was no radiological progression of her wrist, elbow or shoulder joints and her symptoms remained stable.

In December 2023 she developed a large pulmonary embolism and then developed severe bilateral lower limb swelling. There was only mild proteinuria, no right heart failure on echo, normal venous doppler and CT abdomen/ pelvis. The patient suspected denosumab was the culprit and it was stopped in July 2023 with no improvement in swelling. Only after discontinuing fentanyl did her swelling resolve. Since 2023, however, there have been significant classical MCTO erosive changes in the hands, feet and right shoulder. The plan is to rechallenge with 120 mg denosumab and monitor symptoms and radiological changes.

2008 – age 10

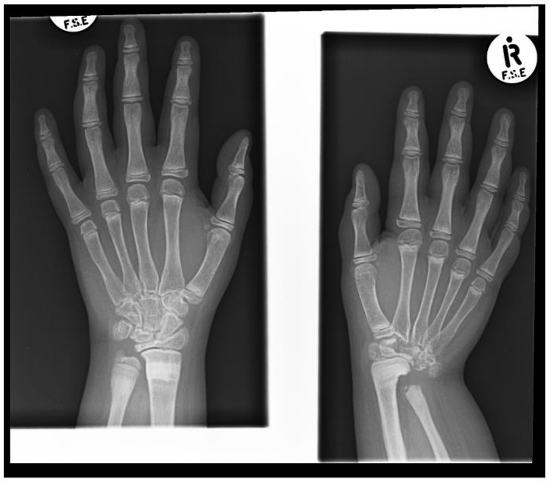

2021 – age 23

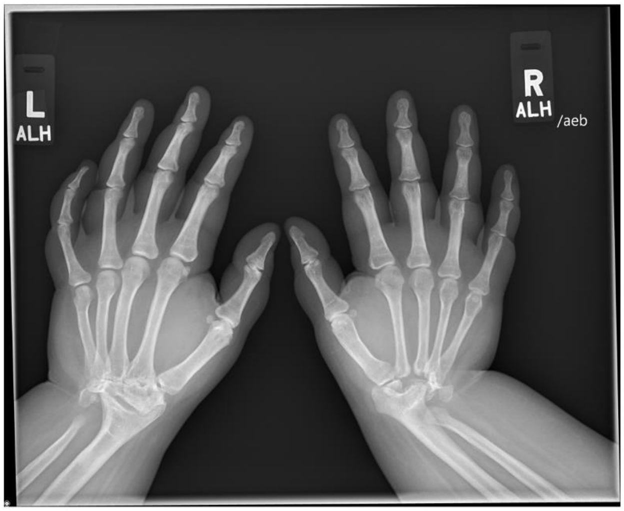

### OC4.1

### Accumulation of pyrophosphate in hypophosphatasia alters calcium phosphate phase transitions and bone mineral ultrastructure

Dr Scott Dillon[1], Ms Amelia Armiger[1], Dr Adrian Murgoci[1], Dr Fabiana Tabegna[1], Dr Linda Skingle[1], Professor Mark Garton[2], Professor Ken Poole[1], Professor Melinda Duer[1]

[1]University of Cambridge, Cambridge, UK. [2]Nuffield Health Hospital Shrewsbury, Shrewsbury, UK

**Abstract**

Hypophosphatasia (HPP) is a rare bone disease caused by inactivating mutations in tissue non-specific alkaline phosphatase (TNAP) responsible for hydrolysing the mineralisation inhibitor pyrophosphate (PPi). HPP patients present with a wide spectrum of clinical symptoms and severity, ranging from stillbirth to osteomalacic fractures and premature loss of deciduous teeth. A rare subset of compound heterozygous HPP patients develop brittle bilateral femoral fractures, phenotypically similar to atypical femoral fractures (ATFs), however the pathophysiology of these fractures remains unclear.

Transiliac crest bone biopsies were obtained from patients undergoing clinical examination for metabolic bone disease at Addenbrooke's Hospital and embedded in LR White resin. Biopsies were obtained from 2 compound heterozygous HPP patients, 3 patients with other forms of osteomalacia (OM), and 3 patients which were found to have no mineralisation defect. Ethical approval for storage and investigation of biopsies was approved by the NHS Health Research Authority and local Research Ethics Committee.

Bone ultrastructure in patient samples was investigated by backscattered scanning electron microscopy, revealing large accumulations of unmineralised osteoid on bone surfaces in both HPP and OM samples. However, energy dispersive X-ray spectroscopy (EDX) showed a significantly reduced ($p < 0.05$) Ca:P ratio in HPP compared to OM samples and controls, indicating the presence of a distinct calcium phosphate phase. High-resolution transmission electron microscopy (HR-TEM) revealed nanoscopic and tightly-packed mineral crystals in HPP bone, compared with stacks of mineral platelets (1-3nm in width) in OM and control samples.

The molecular structure of bone mineral in biopsy serial sections was investigated by solid state nuclear magnetic resonance (ssNMR) spectroscopy. $^{31}P\{^{1}H\}$ 1D cross polarisation spectra demonstrated a decrease in linewidth and a shift towards low frequency in HPP samples ($p < 0.05$) indicating increased mineral crystallinity and an altered bone mineral chemistry. 2D $^{1}H$-$^{31}P$ frequency-switched Lee-Goldburg experiments showed a loss of structural water and hydrated amorphous phases in HPP (Figure 1), indicating bone mineral is driven towards highly crystalline hydroxyapatite in these patients.

We therefore hypothesised that accumulation of PPi in compound heterozygous HPP patients alters calcium phosphate phase dynamics during biomineralisation. To confirm this we incubated α-tricalcium phosphate, which is known to transform to octacalcium phosphate in solution, in the presence sodium pyrophosphate (5%, 10% and 15% molar ratios) for 10 days at 37°C. HR-TEM and ssNMR confirmed the presence of extremely crystalline nanoscopic hydroxyapatite in the solid phase of PPi-containing reactions while control reactions retained amorphous hydrogen phosphate phases.

**Figure 1:**

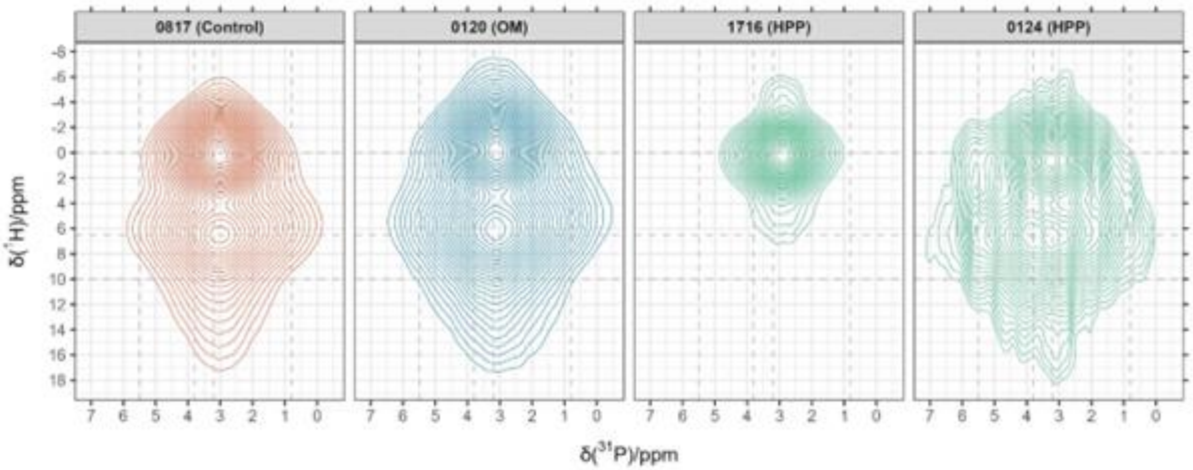

0817 (Control)
0120 (OM)
1716 (HPP)
0124 (HPP)
δ(¹H)/ppm
δ(³¹P)/ppm

### OC4.2

### Shedding light on Matrix Vesicles: Using fluorescent TNAP and PHOSPHO1 to study MV biogenesis

Miss Charlotte Clews[1], Dr Scott Dillon[2], Professor Fabio Nudelman[1], Professor Colin Farquharson[1], Dr Louise Stephen[1]

[1]University of Edinburgh, Edinburgh, UK. [2]University of Cambridge, Cambridge, UK

### Abstract

Biomineralisation is a crucial process undertaken by cells in mineralising tissue, whereby hydroxyapatite is deposited at the extracellular matrix (ECM), creating a tough, stiff organic-inorganic composite to meet bone's physiological needs. The balance of inorganic phosphate and pyrophosphate regulates ECM mineralisation which is controlled by key phosphatases, tissue non-specific alkaline phosphatase (TNAP) and PHOSPHO1. These phosphatases are present within matrix vesicles (MVs), small membrane-bound particles secreted by cells in mineralising tissue. MVs act to concentrate calcium and phosphate ions, initiating MV-driven biomineralisation. Despite their importance, the biogenesis, composition and exact role of MVs in mineral deposition remains unclear.

To address the remaining questions surrounding MV biogenesis we fluorescently tagged recombinant TNAP and PHOSPHO1 to produce an MV-reporter cell line (PHaNTAM). Colocalisation of these phosphatases was identified within the cell and in a subpopulation of vesicles extracted from mineralising ECM and isolated using small particle FACS. We therefore confirm that PHaNTAM cells are a novel method for studying MVs. Using super-resolution microscopy, we were able to follow MV-precursors through the cell, identifying a RAB8 and microtubule driven process, reminiscent of the exosome secretion pathway. Exosomes are small vesicles involved in intercellular communication and transport. They are formed within multivesicular bodies (MVBs), membrane bound compartments which fuse with the membrane, to release exosomes into the ECM. This is counter to classic studies of MV biogenesis, which suggest they are produced by ‘blebbing’ from the cell surface, but supports more recent hypotheses that there may be multiple MV populations, produced via different pathways.

To further improve our understanding of MV biogenesis, we visualised mineralising PHaNTAM osteoblasts *in-vitro*, utilising Correlative Light and Electron Microscopy (CLEM). This technique allows us to combine the detailed structural subcellular information afforded by EM with the specific protein analysis of the fluorescent imaging. We observed fluorescent PHOSPHO1 and TNAP localisation within the mineralising osteoblast, within 200 – 400 nm sized intracellular vesicles, corresponding with an MVB, exosome-like intracellular trafficking pathway. Furthermore, we identified PHOSPHO1 and TNAP localisation within and in close proximity to electron-dense mitochondria. This supports the mounting evidence that mitochondria play an important role in supplying phosphate and calcium ions destined to be used for mineralisation.

In conclusion, PHaNTAM cells offer a robust and exciting new tool for the continued study of MV-driven biogenesis, in particular opening up new avenues for the study of the early stages of MV biogenesis.

### OC5.1

### Vertebral Fracture Assessment and progression of vertebral fractures prior to bisphosphonate treatment in boys with Duchenne muscular dystrophy (DMD)

Dr Nicola Crabtree[1,2], Dr Sarah McCarrison[3,4], Dr Vrinda Saraff[1,2], Dr Sheila Shepherd[3,4], Dr Suma Uday[1,2], Dr Sze Choong Wong[3,4]

[1]BWC NHS Trust, Birmingham, UK. [2]University of Birmingham, Birmingham, UK. [3]Royal Hospital for Children, Glasgow, UK. [4]University of Glasgow, Glasgow, UK

### Abstract

**Objectives:** Current (2018) International Care Considerations for DMD recommend regular spine monitoring for vertebral fracture (VF) and initiation of bisphosphonates following identification of moderate or severe VF even without back-pain or VF of any grade with back-pain. Our objective was to investigate the progression in vertebral fractures and pain in the twelve months preceding treatment with intravenous bisphosphonate therapy.

**Methods:** A dual-centre retrospective study of boys with DMD initiated on IV bisphosphonates due to concerns about VF were identified from spine monitoring between 2015 and 2023. Inclusion criteria was spine imaging immediately prior to bisphosphonate treatment and at least one lateral spine image in the preceding 12 months. Annual spine monitoring was performed with lateral dual-energy X-ray absorptiometry or lateral spine radiographs. For purposes of this study, anonymized spine images were centrally reported by a single observer. Boys were categorised according to severity of VFs at time of treatment; spine deformity index (SDI) < 6 = MILD or SDI >= 6 = SEVERE.

**Results:** 39 boys with DMD on daily glucocorticoids were included. Mean age of first bisphosphonate treatment was 11.4 [7.6-17.6] years. Mean duration of glucocorticoid therapy was 5.4 [0.9-10.6] years. At initiation of bisphosphonates 22 (56%) had moderate or severe VFs of whom 16 (73%) had back pain, 9 (23%) had mild VFs of whom 4 (44%) had back pain and a further 8 (21%) had mild height loss (10-20%) of whom 6 (75%) had back pain. Categorising by SDI, 26 and 13 boys were allocated to the MILD and SEVERE groups, respectively. Boys in the SEVERE group had significantly higher SDI compared with those in the MILD group twelve months prior to treatment [5.6 (3.9) vs. 1.8 (2.3); $p<0.001$] and progressed to more severe, painful fractures at the time of their first infusion [SDI 14.0 (6.5) vs 3.7 (2.3); $p<0.001$].

**Conclusion:** VFs in boys with DMD, on daily glucocorticoids, deteriorate faster with more back pain before treatment is initiated, when 12-month preceding SDI scores are higher. Early treatment, for mild VFs or even mild height loss is likely to stem further VFs and more importantly significantly reduce future back pain. These results could be used as a basis for a review of the 2018 Care Considerations.

### OC5.2

### Bone Mineral Density and Cardiovascular Diseases: A Two-sample Mendelian Randomisation Study

Dr Dorina-Gabriela Condurache[1,2], Dr Ahmed M. Salih[1,3,4], Ms Stefania D'Angelo[5], Dr Elizabeth M. Curtis[5,6], Professor Steffen E. Petersen[1,2], Dr Andre Altmann[7], Professor Nicholas C. Harvey[5,6], Dr Zahra Raisi-Estabragh[1,2]

[1]William Harvey Research Institute, NIHR Barts Biomedical Research Centre, Queen Mary University of London, London, UK. [2]Barts Heart Centre, St Bartholomew's Hospital, Barts Health NHS Trust, London, UK. [3]Department of Population Health Sciences, University of Leicester, Leicester, UK. [4]PRIME Lab, Scientific Research Center, University of Zakho, Zakho, Iraq. [5]MRC Lifecourse Epidemiology Centre, University of Southampton, Southampton, UK. [6]NIHR Southampton Biomedical Research Centre, University of Southampton and University Hospital Southampton NHS Foundation Trust, Southampton, UK. [7]The UCL Hawkes Institute, Department of Medical Physics and Biomedical Engineering, University College London, London, UK

### Abstract

Cardiovascular disease (CVD) and bone health are significant contributors to global mortality, morbidity, and healthcare costs. While strong associations between these conditions have been established, it remains unclear whether these links are causal or driven by shared risk factors. To address this, we performed a comprehensive two-sample Mendelian Randomisation (MR) analysis to assess the causal relationship between BMD and seven CVDs, leveraging summary statistics from extensive genome-wide association studies (GWAS).

Genetic instruments were selected based on stringent quality control criteria, ensuring genome-wide significance ($p < 5 \times 10^{-8}$) and independence (linkage disequilibrium $r^2 < 0.001$ within a 10,000 kb genomic window). Summary-level GWAS data for total-body BMD were sourced from a European GWAS meta-analysis of 66,628 participants. CVD-related summary statistics, including data for atrial fibrillation, angina, ischaemic heart disease, heart failure, hypertension, myocardial infarction and non-ischaemic cardiomyopathy, were extracted from European-descent individuals in UK Biobank, using data from three published GWASs. To validate these findings, an independent analysis was conducted using GWAS data from the FinnGen consortium, comprising 224,737 participants of European ancestry. Causal estimates were calculated primarily using inverse variance weighted (IVW), with additional sensitivity analyses including weighted median, weighted mode and MR-Egger regression (MR-Egger) and MR pleiotropy residual sum and outlier (MR-PRESSO).

The analysis included 85 lead single nucleotide polymorphisms (SNPs) derived from GWAS data of total-body BMD. No causal association was found between genetically predicted BMD and the seven CVDs, including atrial fibrillation (IVW-estimated β: 0.011, SE: 0.03, p=0.73), angina (β: 0.04, SE: 0.03, p=0.17), ischaemic heart disease (β: 0.009, SE: 0.03, p=0.74), myocardial infarction (β: 0.02, SE: 0.03, p=0.36), heart failure (β: 0.004, SE: 0.04, p=0.91), hypertension (β: -0.01, SE: 0.01, p=0.44) and non-ischaemic cardiomyopathy (β: 0.1, SE=0.08, p=0.20). Furthermore, validation analysis using an independent Finnish dataset yielded null associations across all methods, reinforcing the absence of a causal link (Figure 1).

This study found no evidence supporting a causal relationship between genetically predicted BMD and multiple CVDs. Previously reported associations may be attributable to shared risk factors rather than causative mechanisms. Therefore, public health efforts should focus on modifying shared risk factors to reduce the burden of bone-related and cardiovascular conditions.

Figure 1. Impact of SNPs on BMD and cardiac diseases

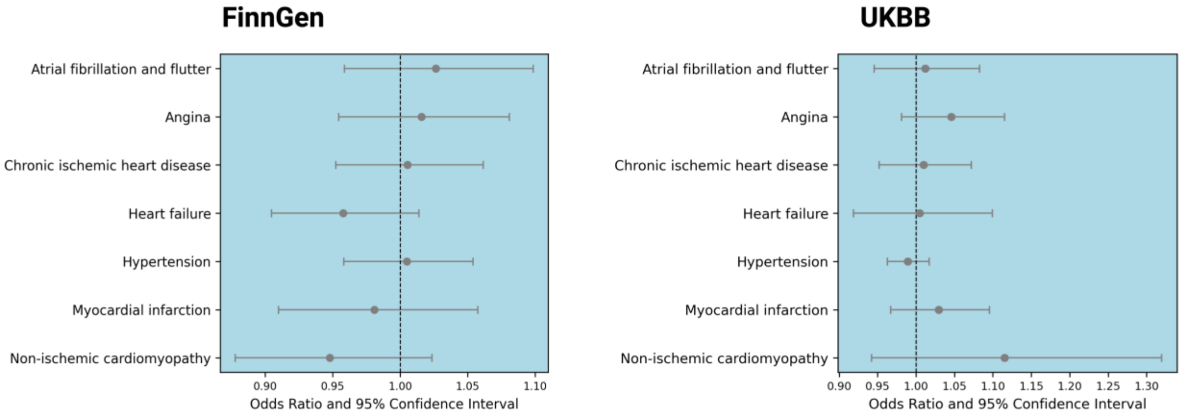

**Footnote:** The scatter plots from the Mendelian Randomisation (MR) analysis illustrate the statistical relationship between genetically predicted BMD and various cardiac diseases. The x-axis represents the odds ratio and 95% confidence intervals for the genetic association between BMD and outcomes, derived from both the UK Biobank (UKBB) and FinnGen cohorts. The y-axis lists the cardiac outcomes analysed, including atrial fibrillation and flutter, chronic ischaemic heart disease, heart failure, hypertension, myocardial infarction, and non-ischaemic cardiomyopathy. The MR methods used include inverse variance weighted (IVW), MR-Egger, simple mode, weighted median, and weighted mode. Results are presented separately for each cohort to validate findings across independent datasets.

### OC6.1

### Recapitulation of developmental bone formation pathways using lineage-specified pluripotent stem cells

Mr. Jiadong Liu[1], Dr. Hongqiang Yu[1], Dr. Suzanne Eldridge[2], Prof. Francesco Dell'Accio[2], Prof. Eileen Gentleman[1], Prof. Agamemnon Grigoriadis[1]

[1]King's College London, London, UK. [2]Queen Mary University of London, London, UK

### Abstract

The ability to regenerate functional bone tissue for treatment of bone defects and tissue engineering strategies requires a thorough understanding of the embryonic programmes that drive the two different ossification pathways, specifically, intramembranous ossification where bone is formed directly from osteogenic precursors, and endochondral ossification, where bone is formed via a cartilage intermediate from chondro/osteogenic precursors. In this study, we have used a directed differentiation approach of pluripotent stem cells (PSCs) combined with 3D hydrogel encapsulation to investigate the different growth factor requirements that drive intramembranous and endochondral ossification.

We have derived a population of chondro/osteo precursors from mouse embryonic stem cells (mESCs) cultured in defined media through a step-wise protocol involving (1)primitive streak/mesoderm specification, (2)mesoderm enrichment and (3)further terminal differentiation. Addition of GDF5 to the PSC-derived mesodermal precursors induced a chondro/osteogenic population that can produce hypertrophic chondrocytes and osteoblasts as confirmed by histology and increased expression of chondro/osteo gene markers(*Sox9*, *Col2*, *Col10*, *Runx2*, *Osx*). The GDF5-induced population displayed self-forming 3D structures with osteoblasts surrounding chondrocytes in multilayered nodules. Replating GDF5-induced nodules enriched the osteogenic potential at the expense of the chondrocyte phenotype in a seeding density-dependent manner with mineralisation potential (von Kossa). Thus, differentiation of this GDF5-induced population appeared to recapitulate an endochondral ossification-like developmental sequence. Interestingly, manipulating the mesoderm enrichment phase of mESC differentiation using time-dependent BMP4 addition yielded a population of precursors that had osteogenic potential with no chondro-differentiation. This was confirmed by osteoblast marker gene expression(*Runx2, ALP, BSP, OCN)* and *in-vitro* mineralisation. This BMP4-induced population therefore seemed to recapitulate an intramembranous ossification-like developmental pathway.

For 3D hydrogel studies, GDF5 and/or BMP4-treated dissociated cells showed good survival/proliferation and formed colonies with cartilage/bone matrix deposition in either hyaluronic acid-based or viscoelastic alginate hydrogels, and qPCR analysis confirmed that encapsulated cells expressed the appropriate chondrogenic and osteogenic marker genes. The influence of mechanical cues/stiffness on 3D-encapsulated cell differentiation also showed differential responsiveness of chondro/osteogenesis.

Finally, we have begun to explore the *in-vivo* potential of GDF5-induced chondro/osteogenic cells and performed xenografts on chick chorioallantoic membrane(CAM), which showed endochondral bone-like tissue developing from grafted GDF5-treated cells. Further mouse intramuscular transplantation of GDF5-induced chondro/osteogenic cells encapsulated in a hyaluronic acid hydrogel showed formation of mineralised bone-like tissues (microCT and histology).

Overall, our results show that differential and temporal addition of GDF5 and BMP4 in PSC-derived mesodermal populations can produce chondrogenic and osteogenic lineages that recapitulate endochondral and intramembranous developmental ossification pathways *in-vitro* and *in-vivo*.

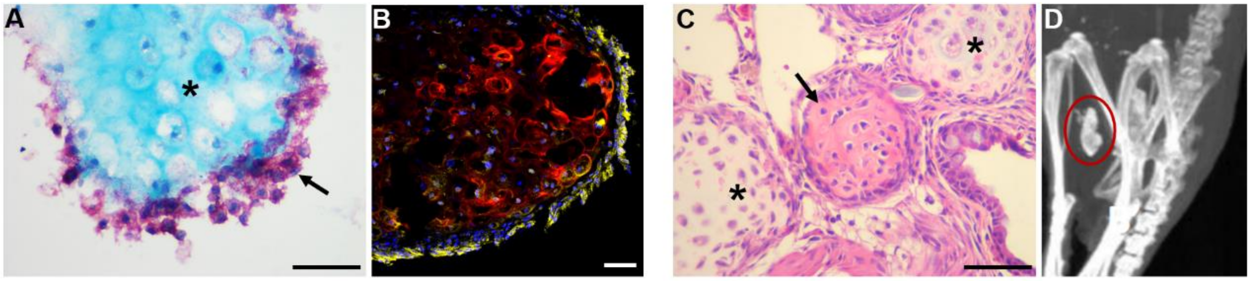

(A) Alkaline phosphatase (arrow) and Alcian blue (*) co-staining of GDF5-induced cells *in vitro*. (B) Collagen X (red) and osteocalcin (yellow) immunofluorescence co-staining of GDF5-induced chondro/osteo nodules. (C) H&E staining of GDF5-induced nodules after grafting onto a CAM showing hypertrophic chondrocyte (*) and bone (arrow) tissue formation. (D) MicroCT image of mineralised bone-like tissue formed from i/m grafting of GDF5-induced PSCs encapsulated in a hyaluronic acid hydrogel. Scale bar: 50μm.

### OC6.2

### Scaffold-free biphasic and triphasic spheroid co-culture for 3D in-vitro enthesis model development

Dr Vinothini Prabhakaran, Dr Jennifer Paxton

Edinburgh medical school: Biomedical sciences, University of Edinburgh, Edinburgh, UK

### Abstract

**Introduction:**

The tendon-to-bone enthesis is an anatomically complex structure with four structurally continuous yet compositionally distinct zones: tendon, uncalcified fibrocartilage, calcified fibrocartilage and bone. Injury to this site is challenging to repair, often resulting in the formation of mechanically weak fibrovascular scar tissue. This study aims to develop a scaffold-free 3D in-vitro model of enthesis to understand its development and repair processes. Spheroids were used in this study due to their self-assembling and self-organising property, enhancing biomimetic relevance. We hypothesise that stem cell (BMSC) spheroids co-cultured between tendon and bone cell spheroids will allow the production of a fibrocartilaginous interface.

**Materials and methods:**

Spheroids of differentiated rat osteoblasts (dROb;day 15) and rat tendon fibroblasts (RTF;day 2) were deposited together as **biphasic** culture in 96-well cell repellent plates. Furthermore, bone marrow stem cell spheroids (BMSC;day 2) were deposited in between dROb and RTF spheroids as **triphasic** culture. The fusion kinetics such as total length, contact length and inter-spheroid angles were evaluated in both biphasic and triphasic co-culture in growth media (GM) and chondrogenic media (ChM) on different days i.e., day 2,4,6,8,10,15&20 using ImageJ/Fiji. The fused spheroids were subjected to H&E, picrosirius red, toluidine blue, alizarin red staining and collagen II immunohistochemistry to evaluate the cellular organisation and tissue-specific extracellular matrix (ECM) synthesis.

**Results and discussion:**

Complete fusion between spheroids occurred within 6-8 days in biphasic co-culture and 15-20 days in triphasic co-culture. Both biphasic and triphasic co-cultures in GM exhibited sparse cell distribution, necrotic cores and lack of ECM over time. In contrast, ChM maintained compact cells with fewer necrotic cores and increased ECM (collagen fibres and sulphated proteoglycans). This indicates that cell interactions alone do not induce BMSC differentiation into fibrocartilage; chondrogenic media is essential for this process.

Compared to biphasic co-culture, the triphasic co-culture in ChM exhibited a continuous BMSC interface connecting the tendon and bone regions, more closely resembling the structural anatomy of the enthesis (Fig.1). Compositionally, the BMSC region in triphasic co-culture showed the presence of collagen I, sulphated proteoglycans and some collagen type II indicating fibrocartilage production (Fig.1). However, chondrogenic media also influenced tendon and bone regions promoting fibrocartilaginous ECM and reducing calcium deposits by dRObs over time.

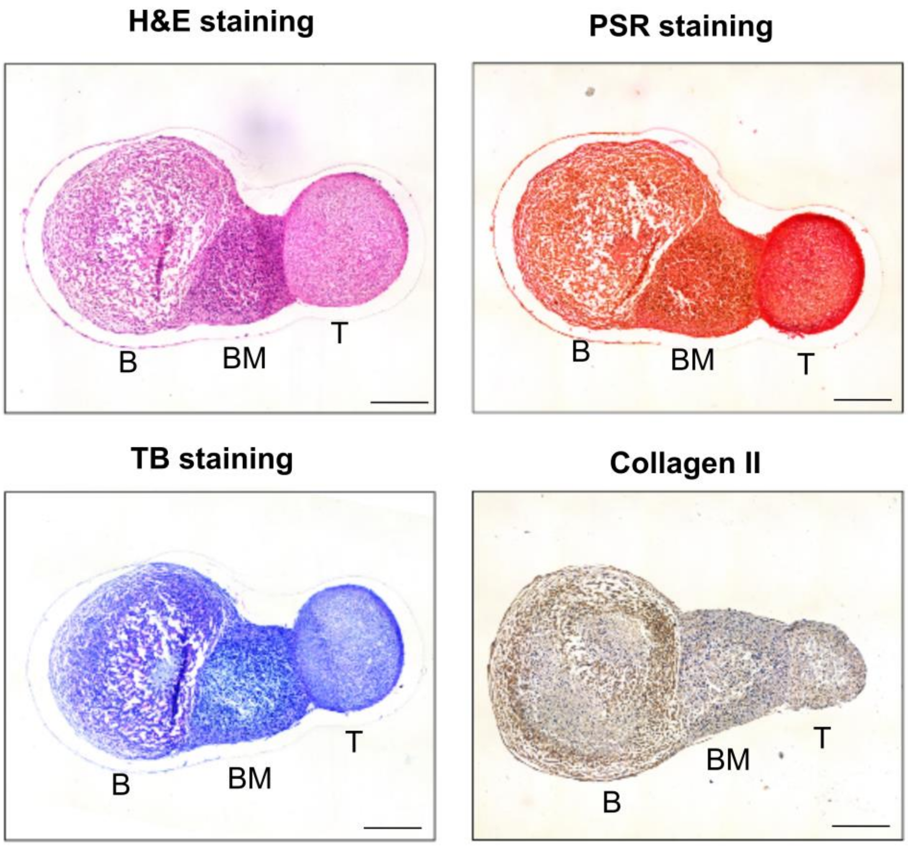

**Figure 1:** Triphasic co-culture of dRObs, BMSC and RTF spheroids stained by H&E, picrosirius red, toluidine blue and collagen II immunohistochemistry. 'B' indicates bone region, 'BM' indicates BMSC interface region and 'T' indicates tendon region. Scale bar: 500 μm.

**Conclusion:**

The study findings support the research hypothesis that triphasic co-cultures can produce structurally continuous fibrocartilaginous interface, but also highlight the importance of optimising media composition to selectively support distinct cell types in this co-culture.

### OC7.1

### PAR2 deletion in the osteoblast lineage affords long-term cartilage protection in experimental osteoarthritis

Dr. Carmen Huesa[1], Dr. Sarah McGrath[2], Ms. Lynette Dunning[1], Ms. Maria Laura Vieri[1], Dr. Kendal McCulloch[3], Dr. Kathryn A. McKintosh[4], Prof. Robin Plevin[4], Prof. Andrew D. Rowan[5], Prof. Rob van 't Hof[6], Prof. Willam R. Ferrell[1], Prof. John C. Lockhart[3], Prof Carl S. Goodyear[1]

[1]University of Glasgow, Glasgow, UK. [2]Ludwig Maximilian University of Munich, Munich, Germany. [3]University of the West of Scotland, Paisley, UK. [4]University of Strathclyde, Glasgow, UK. [5]University of Newcastle, Newcastle, UK. [6]University of Liverpool, Liverpool, UK

### Abstract

Research has identified a bone-specific role for protease-activated receptor 2 (PAR2) in murine osteoarthritis development. The absence of PAR2 reduces osteosclerosis and osteophyte formation, crucial in early surgery-induced murine osteoarthritis (OA). However, it remains unclear if PAR2 inhibition offers sustained, long-term protection against OA progression. Additionally, the specific cellular compartments driving these pathological changes are yet to be determined.

The primary objective of this study was to characterize OA pathology in global, chondrocyte-specific, and osteoblast-specific PAR2 knockout mice up to 12 months after OA induction. By doing so, we aimed to elucidate the role of PAR2 in the later stages of OA, when clinical presentation of pain and lack of joint mobility ensue. We also sought to identify whether PAR2 drives OA from the osteoblast or the chondrocyte compartment.

We utilised a murine model of OA, induced through surgical destabilization of the medial meniscus (DMM). PAR2 knockout mice were generated using the Cre-loxP system, allowing for specific deletion of PAR2 in chondrocytes (Col2cre) and osteoblasts (OCNcre). Mice were monitored for up to 12 months post-surgery and assessed for cartilage damage and subchondral bone changes. Histological analyses were performed to evaluate cartilage integrity, while immunohistochemistry and molecular techniques were used to investigate PAR2 expression and signalling pathways in different cellular compartments.

Our findings revealed that wild-type mice exhibited a gradual increase in cartilage damage and loss over time, consistent with progressive OA pathology. In contrast, PAR2 knockout mice showed significantly reduced cartilage pathology (P=0.001), indicating a protective effect of PAR2 deletion, that was maintained with time. Notably, the removal of PAR2 specifically in osteoblasts, but not in chondrocytes, resulted in decreased cartilage damage (Figure 1). This suggests that PAR2 expression in osteoblasts plays a critical role in driving joint deterioration during the later stages of OA. Further interrogation of the osteoblast compartment revealed that PAR2 has a divergent role during osteoblast development and maturation, with bone marrow cultures and knock out neonatal calvarial pre-osteoblasts showed significantly lower alkaline phosphatase (WT=86.6±18, PAR2-/-=47.6±4, P=0.02) and mineralisation (WT=5.3±2.7, PAR2-/-=12.2±5, P=0.02) compared to its function in already differentiated cells, which had a 2 fold increase in mineral formation (P=0.03).

Our study highlights the importance of bone turnover as a mechanism which influences joint degradation, where a change in bone turnover dynamics can lead to a slower cartilage degradation. The study also explores the complexity of PAR2-mediated effects, with age-dependent and cell-specific roles.

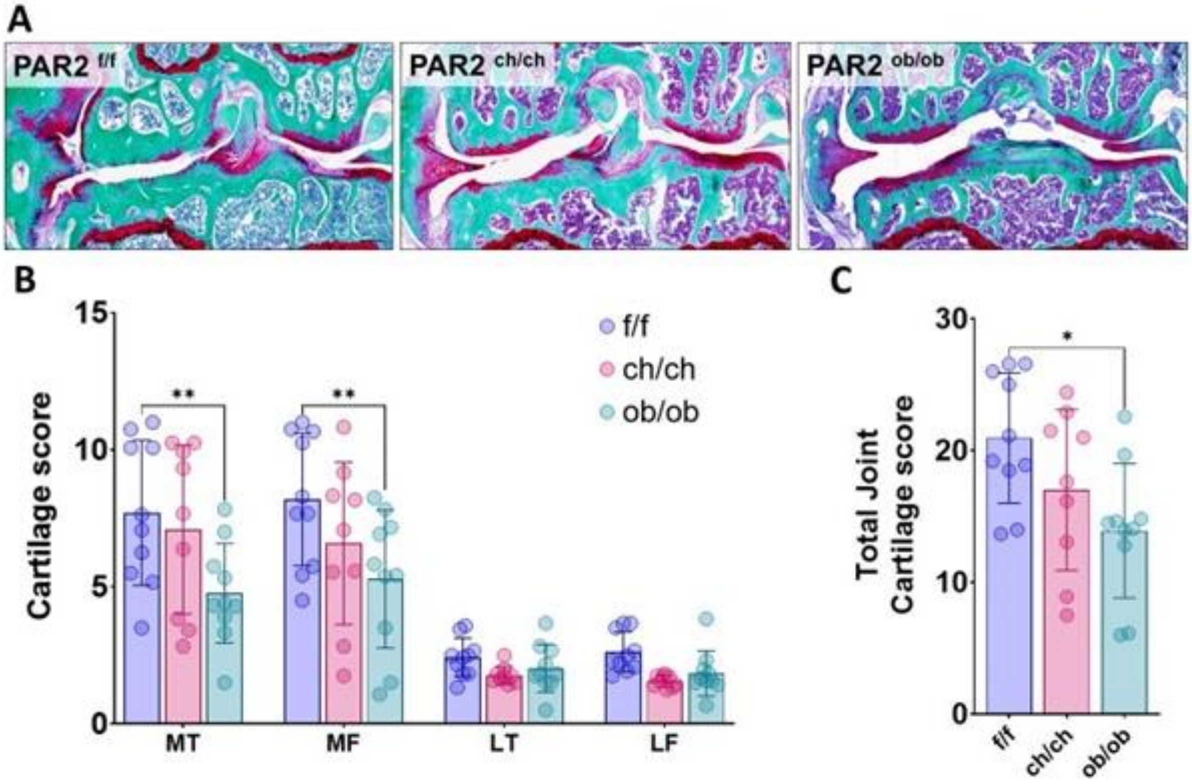

***Figure 1**. Deletion of PAR2 in osteoblasts protects against cartilage damage.*

(A) Representative histological sections stained with SafraninO/Fast green of wild type control PAR2$^{f/f}$ (f/f), chondrocyte driven (PAR2$^{ch/ch}$ , ch/ch) and osteoblast driven (PAR2$^{ob/ob}$ , ob/ob) PAR2 knock out mice 12 months after induction of OA. (B) Quantification of each quadrant of the joint cartilage damage in conditional PAR2 knockouts. MT = Medial Tibial, MF = Medial Femoral, LT = Lateral tibial, LF = Lateral femoral. Data were analysed by 2-way ANOVA with Tukey correction. C) The overall joint damage expressed as the sum of damage in all joint compartments. Data were analysed by one-way ANOVA with Bonferroni correction. Tissue-specific knockouts are compared to the PAR2$^{f/f}$ control. * $P< 0.05$ ** $P< 0.01$.

### OC7.2

### Bisphosphonates Protect Against Oxidative Stress Induced Apoptosis via an FPPS-independent pathway

Dr Helen Knowles[1], Dr Jinsen Lu[1], Dr Srinivasa Rao[1], Dr Anne Horne[2], Prof Ian Reid[2], Prof Graham Russell[1], Prof James Edwards[1]

[1]University of Oxford, Oxford, UK. [2]University of Auckland, Auckland, New Zealand

### Abstract

Bisphosphonates (BP) are the leading medicines targeting disorders of excessive bone loss (eg. osteoporosis, cancer-induced bone disease). BPs inhibit farnesyl pyrophosphate synthase (FPPS) within the mevalonate pathway, impairing protein prenylation. Two placebo-controlled trials have shown Zoledronate reduces mortality whilst several observational studies indicate BPs confer beneficial effects in diverse age-related disorders (cardiovascular disease, cancers, and respiratory infections). Increased accumulation of reactive oxygen species in tissues of older individuals is a key 'hallmark of ageing' and contributing factor to age-related disease. To test whether BPs alter the cellular response to increased oxidative stress seen in ageing organs, we challenged monocytes (THP1), T cells (Jurkat), fibroblast (MRC-5), kidney (HEK) and cardiomyocyte (HL-1) cell lines in an oxidative stress environment ($H_2O_2$) +/- pre-treatment with a panel of BPs, and assessed cellular responses by live cell imaging and involvement of new and established mechanisms of action. Levels of antioxidant proteins following BP treatment in osteopenic patients was also assessed.

High dose BP treatment (>10uM, up to 7d) led to consistent cell death ($p<0.0001$), which was blocked by a key downstream metabolite in the mevalonate pathway, geranylgeraniol (GGOH, 10mM, 3d, $p<0.01$), confirming the role of FPPS-inhibition. Low dose BP treatment (0.1nM-100nM) protected against basal cell death and prolonged lifespan/proliferation in monocytes, fibroblasts, and T cells (up to 67% in 0.1nM ZOL-treated fibroblasts, $p<0.05$). A correlation between basal level of cell death and BP protection was observed ($R^2=0.7679$) where cells with lower levels of basal apoptosis have this reduced even further by 0.1nM BP. Treatment with GGOH did not alter this suggesting a FPPS-independent effect. Oxidative stress-induced apoptosis (50-300uM $H_2O_2$, 24h) was reduced by pre-treatment with BPs (48h) in monocytes, and cardiomyoblasts (0.1nM-1uM, $p<0.001$), with up to 99% protection by 100nM ALN in monocytes ($p<0.001$). Addition of GGOH showed no effect. Osteopenic women treated with ZOL showed significantly increased levels of the plasma antioxidant catalase known to accelerate the decomposition of $H_2O_2$ (log2FC=0.67, $p<0.001$), and reduced levels of pro-oxidants ROCK2, STAT3 and MAPK14. These data suggest treatment with BPs protect against oxidative stress through mechanisms independent of mevalonate pathway inhibition.

### Printed Poster Abstracts

### P01

### Discovery of the core genes of osteoarthritis: a functional evaluation using zebrafish

Mr Rory Stewart[1], Dr Andrii Iakovliev[1], Dr Fraser Collins[1], Dr Athina Spiliopoulou[1], Professor Cosimo De Bari[1], Professor Paul McKeigue[1,2], Dr Erika Kague[1]

[1]Institute of Genetics and Cancer, Edinburgh, UK. [2]Usher Institute, Edinburgh, UK

### Abstract

Osteoarthritis (OA) is a debilitating joint disease, affecting over 500 million people worldwide. Genome-Wide Association Studies (GWAS) have identified over 100 loci associated with OA, however, they have not led to therapeutic breakthroughs. A new genetic idea, the omnigenic hypothesis, suggests that common single nucleotide polymorphisms (SNPs), such as those found in GWAS, are typically of small effect size. It suggests that they influence complex traits/diseases by acting on a sparse set of core genes, through *trans*-effects. Core genes are expected to have a direct effect on disease and phenotype making them attractive therapeutic targets. The objective of the study was therefore to identify OA core genes through genome-wide aggregated *trans*-effects (GATE) analysis, with validation in zebrafish.

We performed GATE analysis on 25,013 hip-OA cases and 459,105 controls from UK Biobank. GATE analysis computes trans-score by aggregating the effects of common SNPs on expression of faraway genes, using large datasets from quantitative trait loci (QTL) studies of gene expression in whole blood (eQTLGen Phase 1) or circulating proteins (Somalogic platform in Iceland, Olink platform in UK Biobank). Association between trans-scores and the trait of interest (OA) was completed using a logistic regression model, while excluding cis-effects and regions such as HLA due its poor 'druggability'. Core genes are those with >5 effective number of trans-QTLs and a p-value<$10^{-6}$.

We analysed single-cell transcriptomic data from synovial fibroblasts and immune cells of humans and mice to prioritise genes for functional validation in zebrafish. Zebrafish orthologs of four putative core genes were targeted using CRISPR/CAS9 to generate zebrafish G0 'crispants' carrying mosaic mutations. Crispants were analysed at 5 days-post-fertilisation to study gene function during joint development, using cartilage staining and linear measurements in ImageJ.

GATE analysis identified 14 putative core genes of OA, including CHAD (LogOR=-0.034, P=1×10-6), SORT1 (LogOR=0.029, P=2×10-5), SPARCL1 (LogOR=0.045, P=2×10-10), and UCMA (LogOR=0.032, P=1×10-6). Our results show that mutations in core genes result in altered cartilage element and joint morphology (Figure).

*Figure: A) Key measurements taken from alcian blue imaging. B) Imaging from alcian blue protocol, 5 days post-fertilisation zebrafish control (wild-type) and crispants. C) Measurement data taken from controls and crispants. i) Ratio of average big length to average small length. ii) Average big width.*

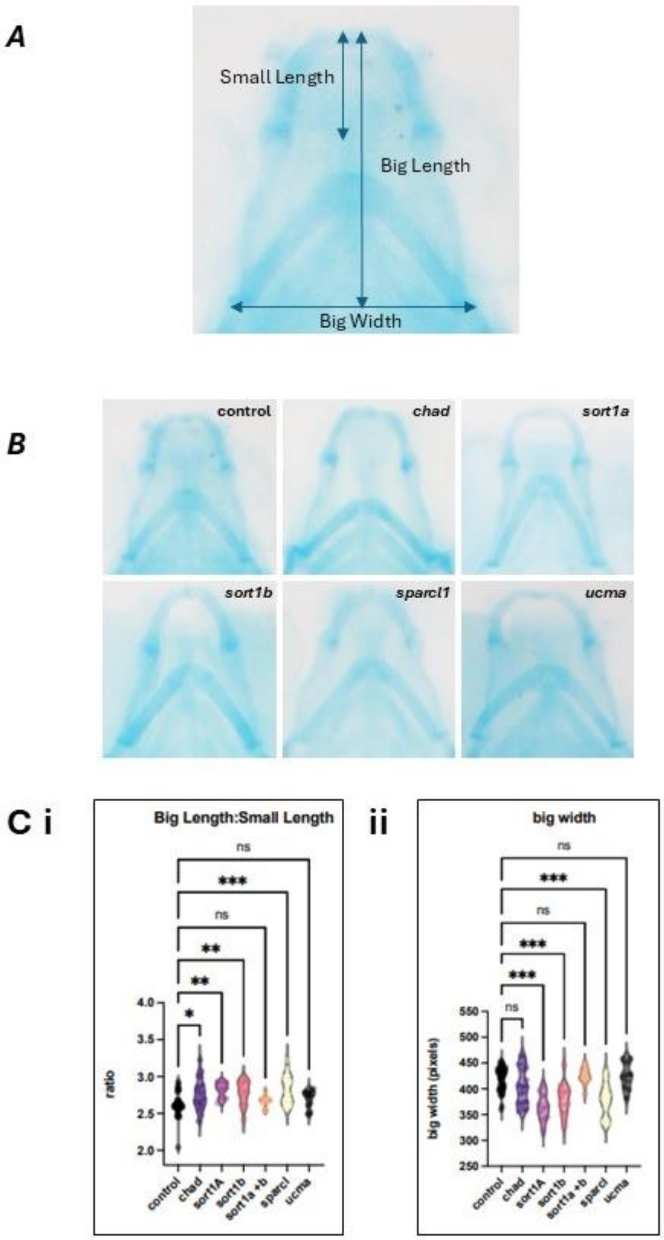

In conclusion, our study identified core genes that cluster into distinct pathways implicated in OA, including bone/cartilage development, neuronal signalling, and inflammation. Our data suggests that changes in core genes of OA may lead to significant impacts on joint health, offering potential targets for future therapeutic interventions.

**P02**

Withdrawn

### P03

### Spleen-derived circulating intercellular adhesion molecule 2 promotes osteosclerosis in chronic myelofibrosis

Miss Sirion Aksornthong[1,2], Dr. Kerstin Tiedemann[1,3], Dr. Mélanie Welman[4], Dr. Alexandra Mazharian[5], Dr. Marie Lordkipanidzé[4,6], Dr. Yotis A Senis[5], Dr. Svetlana V Komarova[1,2,3,7]

[1]Shriners Hospital for Children, Montreal, Canada. [2]Department of Experimental Surgery, McGill University, Montreal, Canada. [3]Faculty of Dental Medicine and Oral Health Sciences, McGill University, Montreal, Canada. [4]Research Center, Montreal Heart Institute, Montreal, Canada. [5]Université de Strasbourg, Institut National de la Santé et de la Recherche Médicale (INSERM), Etablissement Français du Sang Grand Est, Unité Mixte de Recherche (UMR)-S 1255, Fédération de Médecine Translationnelle de Strasbourg, Strasbourg, France. [6]Faculty of Pharmacy, University of Montreal, Montreal, Canada. [7]Department of Biomedical Engineering, University of Alberta, Edmonton, Canada

### Abstract

Mutations in the gene coding G6b-B, *Mpig6b* in mice, and *MPIG6B* in humans lead to myelofibrosis, progressive splenomegaly, and osteosclerosis. Abnormal megakaryocytes in bone marrow and spleen may affect bone cells through cell-cell contact or soluble factors. We aimed to assess the role of circulating factors produced by splenic megakaryocytes in mediating osteosclerosis in $Mpig6b^{-/-}$ mice using a clinically relevant procedure of splenectomy. Splenectomy or sham surgery was performed on 32-week-old female $Mpig6b^{-/-}$ and littermate wild type (WT) mice, when splenomegaly and long bone osteosclerosis were established. Twenty weeks later, the hematopoietic parameters and bone structure of the femur and lumbar vertebra were analyzed. Splenectomy significantly improved platelet count but the larger platelet size persisted in $Mpig6b^{-/-}$ mice. The $Mpig6b^{-/-}$ femurs with established osteosclerosis were not affected by splenectomy. In contrast, splenectomy prevented the development of osteosclerosis in vertebrae of $Mpig6b^{-/-}$mice, which demonstrated significant osteosclerosis in sham-operated $Mpig6b^{-/-}$ mice compared to WT. Moreover, significant bone loss in splenectomized $Mpig6b^{-/-}$ mice compared to WT demonstrated increased bone resorption. Using quantitative plasma proteomics, we identified intercellular adhesion molecule 2 (ICAM2) to be significantly increased in $Mpig6b^{-/-}$ mice compared to WT and reversed to normal levels after splenectomy. The binding of membrane-bound ICAM2 and MAC1 was reported to be critical for osteoclast fusion. In vitro study of osteoclastogenesis using bone marrow-derived macrophages (BMM) demonstrated that soluble recombinant ICAM2 dose-dependently inhibited osteoclast fusion, suggesting that competitive binding of MAC1 with soluble ICAM2 prevented its interaction with membrane-bound ICAM2. Thus, we identified the soluble ICAM2 as a novel inhibitor of osteoclast fusion and mediator of osteosclerosis in $Mpig6b^{-/-}$ mice potentially via the megakaryocyte-spleen-bone axis.

WT mouse
Spleen histology
Spleen
Blood
Bone
Fusion
Mature osteoclast
Bone resorption
Healthy bone
$Mpig6b^{-/-}$ mouse
Abnormal splenic MKS
Splenomegaly
Soluble ICAM2 &
Thrombocytopenia
Fusion
Mature osteoclast
Osteoclerosis
Bone resorption
Megakaryocyte
Platelet
Osteoclast precursor
Mature osteoclast
Soluble ICAM 2
Membrane-bound ICAM 2
Membrane-bound MAC 1

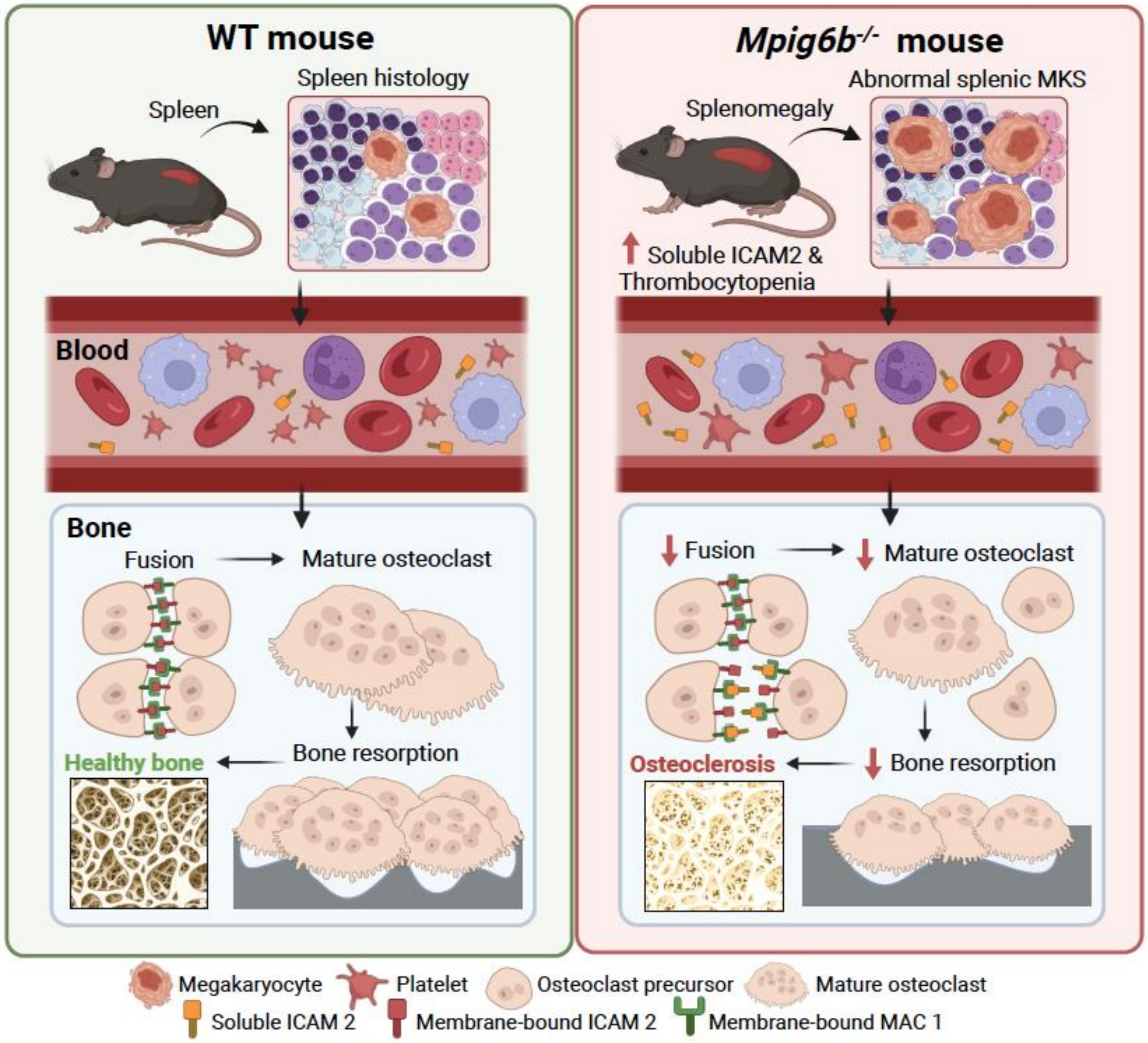

### P04

### Bispecific antibody for sclerostin and DKK1 improves bone health in chronic kidney disease mouse model

Mr. WORACHET PROMRUK[1,2], Dr William P Cawthorn[3], Prof Katherine A Staines[4], Mr. Hua Zhu Ke[5,6], Dr Xiaofeng Liu[5,6], Dr Louise Stephen[1], Prof Colin Farquharson[1]

[1]The Roslin Institute, University of Edinburgh, Edinburgh, UK. [2]Chulabhorn Royal Academy, Bangkok, Thailand. [3]Centre for Cardiovascular Science, The Queen's Medical Research Institute, University of Edinburgh, Edinburgh, UK. [4]School of Pharmacy & Biomolecular Sciences, University of Brighton, Brighton, UK. [5]Angitia Biopharmaceuticals, Woodland Hills, California, USA. [6]Angitia Biopharmaceuticals, Guangzhou, China

### Abstract

Chronic kidney disease (CKD) is a progressive chronic disease characterised by a gradual loss of kidney function and/or structure, leading to an alteration of calcium and phosphate homeostasis and bone loss, commonly referred to as renal osteodystrophy. Sclerostin and DKK1 are secreted by osteocytes and inhibit bone formation and induce bone marrow adipogenesis through their ability to antagonize Wnt signalling. Systemic concentrations of sclerostin and DKK1 are increased in CKD patients and animal models but sclerostin antibodies alone are unable to improve bone mass in CKD mice. Therefore, herein we used a bispecific antibody to sclerostin and DKK1 in an experimental model of CKD to determine whether the inhibition of both Wnt inhibitors can improve bone health and decrease bone marrow adipose tissue (BMAT) accumulation.

To induce CKD, eight-week-old male C57BL/6J mice were fed a diet supplemented with 0.2% adenine for 6-weeks. Each week the adenine diet was offered for 5-days and replaced by a normal diet for 2 days; a protocol that induced CKD but avoided pathological weight loss. Control mice received the same diet without adenine. The bispecific antibody or vehicle was administered to control and CKD mice once a week for 6 weeks (n = 10/group). Plasma was analysed using a biochemistry analyser. Tibia structure and architecture were assessed by micro-CT and BMAT accumulation quantified by micro-CT after osmium staining.

CKD mice treated with vehicle or bispecific antibody had elevated plasma levels of creatinine, blood urea nitrogen, calcium and phosphate compared to their responsive controls. In addition, plasma phosphate concentrations in the antibody-treated groups (CKD and control) were higher than their respective vehicle group. Circulating sclerostin and DKK1 in vehicle-treated CKD were 1-and 5-time higher, respectively than their responsive control and were diminished with bispecific antibody. CKD mice (without the antibody) had osteopenia compared with control mice, indicated by decreased trabecular thickness ($p<0.001$) and increased trabecular separation ($p<0.001$). The bispecific antibody improved trabecular bone parameters in both control and CKD mice when compared to their respective vehicle-treated group. Notably, the bispecific antibody increased trabecular bone fraction ($p<0.001$), thickness ($p<0.001$), and number ($p<0.001$) and decreased trabecular separation ($p<0.001$). Moreover, these anabolic effects of antibody treatment were greater in CKD vs control mice. Quantification of BMAT accumulation is ongoing.

In conclusion, bone health of CKD mice was improved by treatment with the bispecific antibody to sclerostin and DKK1, opening-up new therapeutic options for renal osteodystrophy patients.

**P05**

Withdrawn

### P06

### *In Vitro* Bioactivity and Osteoinductive Potential of Fluorine-Coated WZM211 Magnesium Alloys on Osteoblasts

Dr Roxane Bonithon, Dr Arianna De Mori, Dr Tosca Roncada, Dr Marta Roldo, Pr Gordon Blunn

University of Portsmouth, Portsmouth, UK

### Abstract

INTRODUCTION:

Magnesium (Mg) alloys are increasingly used in orthopaedics due to their mechanical properties and biodegradation closely matching bone properties, reducing stress shielding and eliminating the need for a second surgery. Mg ion release during degradation has been linked to osteogenic differentiation of human bone marrow stromal cells (hBMSCs), but its direct impact on osteoblasts and bone mineralization (i.e. bioactivity) remains unclear. This study quantifies the bioactivity and osteoinductive properties of aluminum-free porous Mg alloys in contact with osteoblasts to assess their influence on bone growth and mineralization.

METHODS:

Primary ovine osteoblasts (N=100,000) from three animals (three replicates each) were cultured on Mg alloys and in 6-well plates (control) for 1, 3, and 7 days in DMEM/F12 with 1% p/s, 10% FBS, and 20 mM HEPES at 37°C, 5% $CO_2$. Viability, proliferation and differentiation were analyzed using Live/Dead staining, Presto Blue, LDH assay and RT-qPCR. Bioactivity was assessed with ImageJ using SEM images after immersion in simulated body fluid (SBF) for 1, 7 and 14 days. Additional characterization was conducted using SEM-EDS to verify the formation of CaP deposits and elucidate the interactions between osteoblasts and the mineralized surface. Statistical analyses were conducted using two-way ANOVA with Tukey's post-hoc test or Kruskal-Wallis test (GraphPad Prism 8.0.2), with significance at $p<0.05$.

RESULTS:

Cell viability and proliferation significantly increased from 1 to 7 days, as shown by Live/Dead (90.2 ± 7.1% to 99.1 ± 0.6%, $p<0.05$) and Presto Blue (2.7 ± 1.2 RFU to 8.1 ± 2.5 RFU, $p<0.005$) assays. RUNX2 gene expression significantly rose ($p<0.001$) on Mg alloys. SBF immersion resulted in a significant ($p<0.005$) increase in CaP deposition from 1.6 ± 0.3% at 1 day to 3.9 ± 0.4% at 14 days. CaP deposits in the form of spherulitic structures formed on contact with osteoblasts and allowed them to anchor to the biomaterial surface (Figure 1).

DISCUSSION:

Mg alloys enhance osteoblast viability, proliferation and differentiation *in vitro*, supporting the potential for substantial bone growth and osteointegration *in vivo*. This study provides the first quantification of Mg alloy bioactivity for up to 2 weeks, demonstrating that Mg alloys biodegradation participates in the formation of CaP-based particle in the vicinity of osteoblasts *in vitro* which favors the mineralization process *in vivo*. These findings suggest that Mg alloys promote bone growth, as well as mineralization potentially improving fracture healing after injury. Further research should explore in vivo effects and long-term degradation to confirm clinical viability.

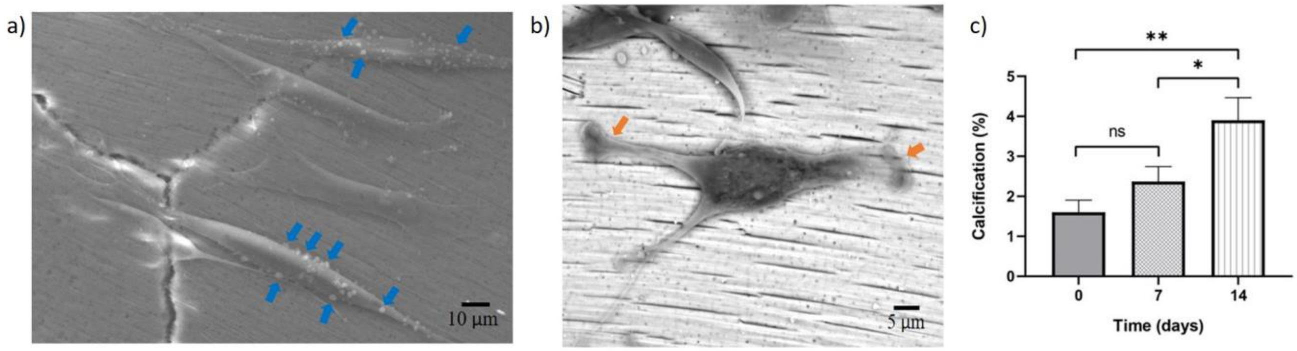

**Figure 1**: a) Secondary electron image (SE) of CaP-based particles deposition (blue arrows) on top of osteoblasts at 2 kX magnification; (b) Backscattered electron image (BSE) of CaP-based particles as cells filopodia anchor (red arrows) at 3.5 kX magnification; (c) Bioactivity with time. *$p<0.05$ and**$p<0.005$ significant difference.

### P07

### High resolution X-ray imaging during whole joint loading reveals focal concentrations of mechanical strains that predict osteoarthritis emergence

Dr Aikta Sharma[1], Dr Lucinda AE Evans[2,3], Dr Lucie E Bourne[2], Miss Alissa L Parmenter[1], Dr Joseph Brunet[1], Dr Kamel Madi[4], Professor Andrew A Pitsillides[3], Professor Peter D Lee[1], Professor Katherine A Staines[2]

[1]University College London, London, UK. [2]University of Brighton, Brighton, UK. [3]Royal Veterinary College, London, UK. [4]3Dmagination, Didcot, UK

### Abstract

Mechanical loading is essential for maintaining joint health and plays a key role in the pathogenesis of osteoarthritis (OA). How loads are transmitted through distinct anatomical regions of the joint during healthy ageing, compared to those that develop OA with age, however, remains unclear[1]. Herein, we use high-resolution synchrotron X-ray tomography (sCT) to characterise the magnitude and 3D distribution of mechanical strain in response to physiological loading across anatomical zones of the tibial epiphyses in intact knees from healthy and OA-prone mice.

Hindlimbs from male OA-prone STR/Ort and healthy parental CBA mice (N=4) at ages prior OA-onset (10-weeks) and advanced-OA (40-weeks) were mounted in bespoke cups[2] on a uniaxial loading rig and subjected to physiological compression in-situ. sCT images of each knee joint were acquired (ESRF; 1.45µm/voxel) following stepwise application of displacement-controlled loads. Following tomographic reconstruction[3], 3D strain magnitudes across tibial epiphyseal zones were quantified by digital volume correlation (DVC, Avizo 3D). Whole hindlimbs were also imaged by microCT (4.98µm/voxel) for growth plate (GP) bridge number and areal density quantification.

In CBA joints, load-induced compression and tension were low and efficiently transferred from articular to metaphyseal regions in both ages. In STR/Ort joints, high compressive and tensile strain foci were localised to articular and GP regions with magnitudes increasing with age. Quantification revealed higher average tension and compression ($p \leq 0.002$) with wider distributions in STR/Ort than CBA, at 10- and 40-weeks (both $p<0.0001$), independent of differences in epiphyseal volume. Regionalised measurements showed laterally-dominant load-induced condylar strains in 10-week-old STR/Ort; where tension and compression were higher in articular calcified cartilage (ACC, $p=0.02$), subchondral bone (SCB, $p \leq 0.03$) and trabecular bone (TB, $p \leq 0.008$). In contrast, strains were symmetrical in 10-week-old CBA tibial epiphyses and remained latero-medially balanced with age, while age-matched STR/Ort sustained tensional imbalances in the ACC ($p<0.04$) and greater compression in lateral ACC and SCB ($p \leq 0.05$). GP bridge number and areal density did not differ in CBA and STR/Ort but increased in both with age ($p<0.0001$).

Our findings reveal regionalised, load-induced strain concentrations appear in OA-prone joints prior to any overt pathology, which may promote early mechanically-driven OA. These early pre-OA strain changes may predetermine pathological architectural changes that collectively contribute to joint failure in advanced OA. In healthy joints, load-related strains are efficiently transferred to remote epiphyseal regions, suggesting that the preservation of epiphyseal architecture with age may mitigate high forces generated at the articular surface to protect against load-induced trauma.

**P08**

Withdrawn

**P09**

Withdrawn

**P10**

Withdrawn

### P11

### Improved accuracy of trabecular bone shape measurement with GPU-accelerated ellipsoid factor

Dr Michael Doube

BoneJ, Tannay, Switzerland

### Abstract

General-purpose graphical processing units (GPUs) have emerged as core technology for performing large numbers of similar calculations in parallel. I implemented Ellipsoid Factor (CPU-EF) [1], part of the BoneJ plugin suite for ImageJ/Fiji [2], in OpenCL GPU device code using the JOCL Java library to interface with host code in BoneJ (GPU-EF). GPU-EF uses Java threads to delegate ellipsoid fitting jobs to all available GPUs on the host machine. Each ellipsoid is optimised while constrained within a cloud of pixels representing the bone boundary. Parallel GPU processing allows a near-total sampling of thousands of boundary pixels, in contrast to CPU-EF which tests a fixed number of samples on the ellipsoid surface (default, 100) to detect ellipsoid-boundary collisions with a time cost that scales with increasing number of sample points. These 100 sample points become more sparse as the ellipsoid grows, leading to decreasing accuracy in large structures and a tendency to fail to be constrained by image features. The large number of boundary points permitted by parallel processing on GPU leads to a more detailed manifold with higher frequency spatial sampling. The optimisation procedure is otherwise the same between the two implementations, consisting of an iterating sequence of ellipsoid dilatation, rotation and small amounts of translation from a seed point, until no increase in volume has been made after a user-set number of iterations. Using 25 no-improvement iterations and similar numbers of seed points results in an improvement in the amount of filled foreground from 99.3 % on CPU-EF to 100.0% on GPU-EF, along with sharper EF histogram peaks, and greater respect for boundaries formed by small image features such as intratrabecular canals (Fig1, arrowhead). On a Latitude 7370 laptop (Dell UK) with a single external GPU (NVIDIA RTX A4000), CPU-EF fit each ellipsoid 3.1 – 3.7 times faster than GPU-EF, taking 8.8 – 9.8 versus 27.0 – 36.2 ms per ellipsoid. However, GPU-EF calculated ellipsoid-boundary collisions using on the order of 100× more pixels per ellipsoid than CPU-EF (>10,000 versus 100), indicating that GPU-EF performs calculations about 30× faster than CPU-EF. Further optimisation will determine whether higher information density due to increased number of boundary pixels allows a similar goodness of fit to be achieved with fewer ellipsoids or optimisation loop iterations.

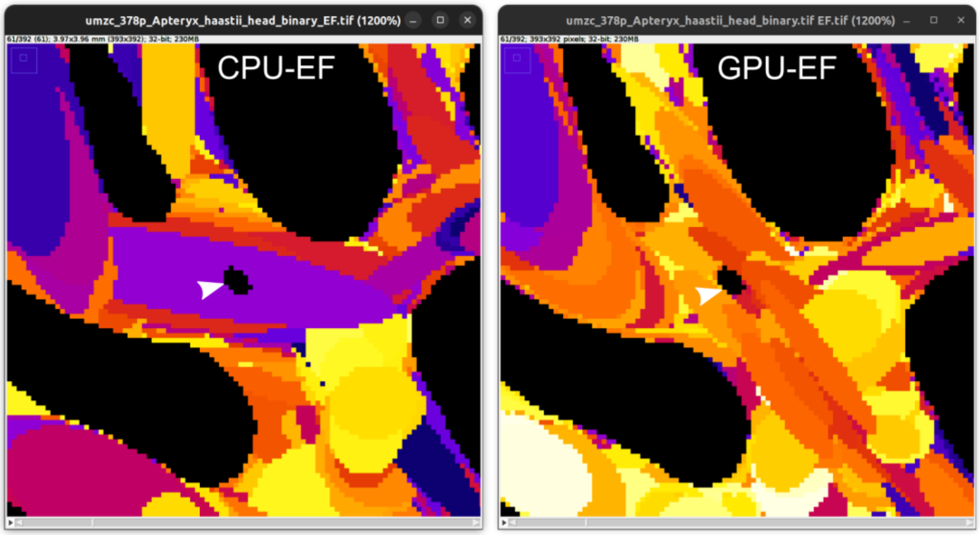

1.Doube M. 2013 The ellipsoid factor. *Bone Research Society Abstracts*, p. 63. (doi:10.3389/978-2-88919-174-1)

2.Domander R, Felder AA, Doube M. 2021 BoneJ2 - refactoring established research software. *Wellcome Open Res.* **6**. (doi:10.12688/wellcomeopenres.16619.2)

### P12

### Modifying bone anchor morphologies in an *in vitro* model of the calcaneofibular ligament

Ms Alissa Lui, Dr Asad Ali, Ms Sophie Mok, Dr Jennifer Paxton

University of Edinburgh, Edinburgh, UK

### Abstract

The calcaneofibular ligament (CFL) is one of the three lateral stabilising ligaments of the ankle joint. The CFL is frequently injured, and attempts to repair have limited success, contributing to long-term ankle instability and reduced quality of life for those affected. To address options for a tissue-engineered CFL replacement, our previous work has identified the size, shape and overall morphologies of the CFL from cadaveric dissection morphometrics. These data have allowed for the production of an anatomically-accurate model of the CFL to be produced *in vitro* as a potential option for CFL repair.

The *in vitro* model is based on existing approaches and contains artificial bone 'anchors', produced to the dimensions of the proximal and distal footprints of the CFL at the enthesis region, and a soft-tissue analogue, manufactured from fibroblast-seeded fibrin hydrogel, to represent the ligament portion of the construct (Figure 1A). 3D printing and molding has been used to create a bespoke tissue culture dish to allow for construct formation and maturation *in vitro* (Figure 1A)

This study has examined the morphologies of the artificial bone anchors used in the model, to improve tissue infiltration and establish a more anatomically accurate connection at the bone-ligament (enthesis) junctions. While previous studies have focussed on the use of brushite cement (Figure 1Aii) low macroporosity and poor adhesion has led to the investigation of bone anchors manufactured from 3D printed biocompatible materials.

Proximal (9.25 × 6.25 × 8 x 5mm) and distal (14.90 x  11.04 x 8 x 5mm) CFL footprints, determined from anatomical dissection morphometrics, were designed at varying strut spacings (solid, low spacing and high spacing, Figure 1B) to be compared to the solid brushite cement anchors used previously. Both polylactic acid (PLA) and polycaprolactone (PCL) have been trialled as 3D printed biocompatible materials in the *in vitro* CFL model. Our current work is focussed on assessing cellular growth and tissue infiltration in the anchor designs and evaluating overall material suitability in the anatomically accurate *in vitro* CFL model.

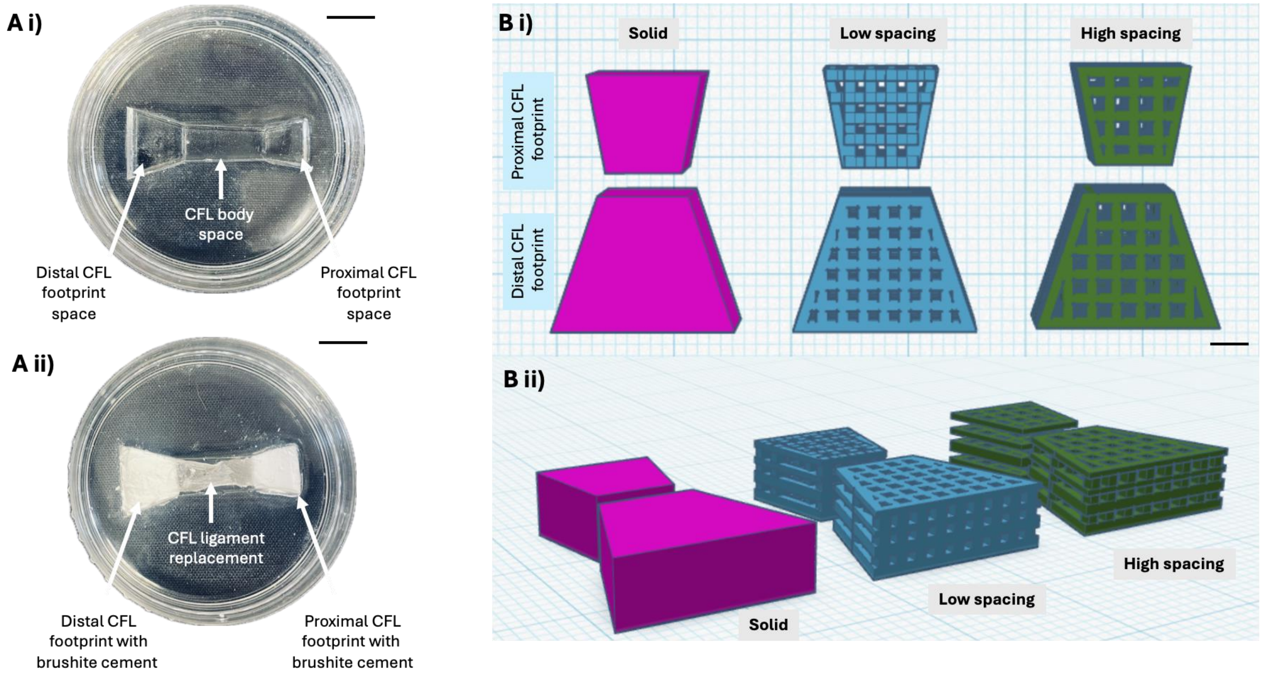

**Figure 1A i)** Bespoke culture plate for CFL construct formation **ii)** Original CFL ligament model using brushite cement as bone anchors and a CFL ligament replacement between the proximal and distal footprints. Scale bar = 1cm **B)** Stereolithography files and designs for the solid, low spacing and high spacing bone anchor designs for 3D printed biocompatible materials. **i)** superior view, Scale bar = 3mm ii) posterolateral view

### P13

### An Axis of Wnt and Proinflammatory Signals Underlies Mechanically Driven Osteogenesis

Dr Hussain Jaffery

University of Glasgow, Glasgow, UK

### Abstract

Mesenchymal stem cells (MSCs), naturally found in diverse bodily reservoirs, serve as multipotent progenitors for osteoblasts, the cells responsible for bone formation. The process of osteogenic differentiation, leading to the formation of bone, is influenced by a myriad of factors, including the response of cells and the entire organism to biomechanical forces. In our research, we have innovated a nanovibrational bioreactor, a cutting-edge biomechanical approach for directing MSCs towards bone formation. Our current research explores the impact of nanovibrational stimulation on the osteogenic Wnt signaling pathway, a critical component in bone development.

Adipose-derived human MSCs were cultured on our novel bioreactor, delivering nanovibrational stimulation of 30 nm vertical displacement, at 1000 Hz in basal medium ("nano-kicked"; NK) compared to controls stimulated with the osteogenic metabolites (OGM) L-ascorbic acid, b-glycerophosphate and dexamethasone, or controls kept in basal conditions with no stimulus (Ctrl), for 1 to 21 days. Parsing of various pathways involved incorporating Wnt inhibitors, including LGK974, XAV939, AMBMP hydrochloride, CHIR99021, alongside Rock inhibitor Y-27632 and BCL3 mimetic peptide BDP2.

(**A**) An axis of proinflammatory, non-canonical Wnt pathway and osteogenic genes all have higher transcriptional expression in nanovibrated stem cells, compared to metabolite-induced cells. (**B**) Treating nanovibrated cells with Wnt pathway inhibitors XAV939 (targeting canonical-Wnt via Tankyrase) and LGK974 (targeting pan-Wnt secretion via Porcupine) reveals divergent gene transcription signatures – generally, LGK974 reduces expression of genes in the axis, while XAV939 increases axis gene expression. (**C**) In the presence of nanovibration, a BCL3 mimetic peptide (inhibitor of NF-kB signalling, associated with non-canonical Wnt) inverted the transcriptional signatures produced by XAV939 and LGK974 inhibition. (**D**) Further studies inhibiting the Wnt pathway with AMBMP and CHIR99021 recapitulated earlier observations that the non-canonical Wnt pathway is integral to forming the axis of osteogenic differentiation. (**E**) Transcriptional observations throughout were validated with protein expression and use of Rock inhibitor Y-27632 demonstrated functional reversal of nanovibrational signal. (**F**) In complement to hMSCs, BCL3 knockout murine monocytes abrogated the increase in axis genes observed in wild-type cells during nanovibration-driven osteoclastogenesis. While nanovibration itself did not increase osteoclast number or size, XAV939 treatment of BCL3 knockout cells had a dramatic increase in osteoclastogenesis – exemplary of the axis of proinflammatory, non-canonical Wnt in bone cell differentiation.

Broadly, a cell signalling axis comprised of non-canonical Wnt signalling, proinflammatory cytokine signalling and NF-kB regulation determines stem cell osteogenic and osteoclastogenic differentiation in response to nanovibrational biomechanical force.

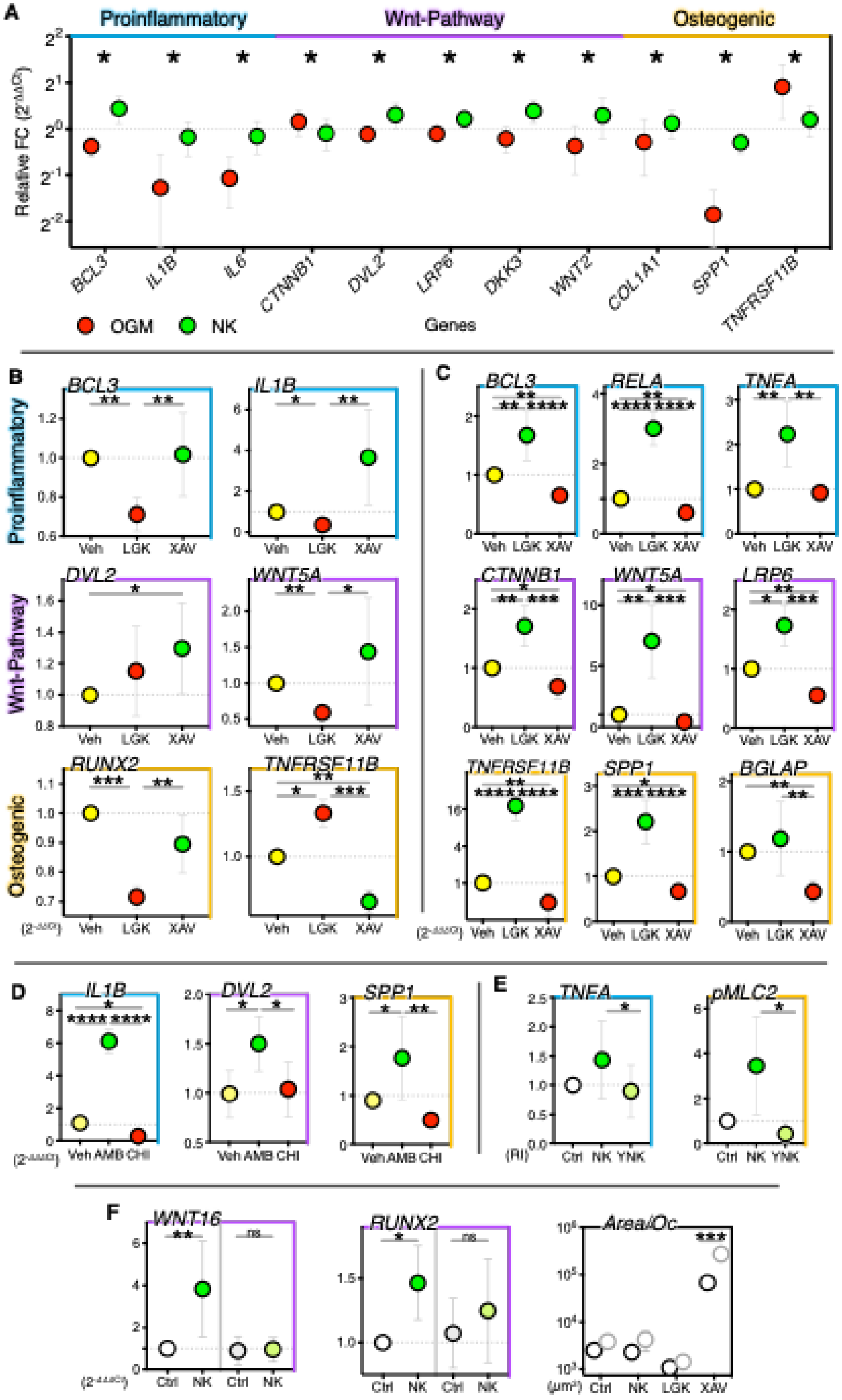

A
Proinflammatory
Wnt-Pathway
Osteogenic
Relative FC (2^-ΔΔCt)
BCL3 IL1B IL6 CTNNB1 DVL2 LRP6 DKK3 WNT2 COL1A1 SPP1 TNFRSF11B
OGM NK
Genes
B
Proinflammatory
Wnt-Pathway
Osteogenic
BCL3 IL1B DVL2 WNT5A RUNX2 TNFRSF11B
Veh LGK XAV
C
BCL3 RELA TNFA CTNNB1 WNT5A LRP6 TNFRSF11B SPP1 BGLAP
Veh LGK XAV
D
IL1B DVL2 SPP1
Veh AMB CHI
E
TNFA pMLC2
(RI)
Ctrl NK YNK
F
WNT16 RUNX2 Area/Oc
Ctrl NK Ctrl NK
(µm²) Ctrl NK LGK XAV

### P14

### Bone marrow adiposity disrupts the dormant niche in multiple myeloma

Dr Young-Eun Park[1,2], Dr Beatriz Gamez[1,2], Professor Claire Edwards[1,2,3]

[1]Nuffield Department of Surgical Sciences, University of Oxford, Oxford, UK. [2]Oxford Centre for Translational Myeloma Research, University of Oxford, Oxford, UK. [3]Nuffield Department of Orthopaedics, Rheumatology and Musculoskeletal Sciences, University of Oxford, Oxford, UK

### Abstract

An increase in bone marrow (BM) adiposity is observed with ageing and obesity, which are major risk factors for multiple myeloma (MM). Our previous studies demonstrated that obesity and BM adiposity are associated with MM pathogenesis, however, their impact on MM dormancy is unknown. AXL is a marker of dormant MM cells, and binding of GAS6 to AXL supports cancer cell survival. Our aim was to determine how changes in adiposity impact expression of AXL, GAS6 and MM dormancy.

Vybrant$^{TM}$-DiI/DiD (DiI/DiD)-labelled 5TGM1-GFP cells (5TGM1s) were cultured with ST2 mouse BM stromal cells or 2T3 pre-osteoblasts treated with mouse adiponectin (5 µg/ml) or GAS6 (250 µg/ml). BM adipocytes (BMAds) were differentiated from primary mouse BM (BM-BMAds) or ST2 (ST2-BMAds). C57BL/KaLwRij mice were fed with either normal or high-fat diet (HFD) for 5 weeks before intravenous 5TGM1-DiI injection. Mice were culled after 21 days.

Differentiation to BMAds increased secretion of AXL and GAS6, however, *axl* and *gas6* mRNA levels were reduced, while *adipoq* level was increased. Co-culture of 5TGM1s with 2T3s reduced the loss of DiI expression and increased AXL expression demonstrating that 2T3s support 5TGM1 dormancy. Co-culture also increased AXL expression in 2T3s. Adiponectin pre-treatment increased AXL expression in co-cultured 2T3 and 5TGM1 cells. Co-culture of 5TGM1s with ST2s induced loss of DiI expression, which was greater when co-cultured with ST2-BMAds. Co-culture increased AXL expression in 5TGM1s but not ST2s or ST2-BMAds. Gene expression analysis showed no change in *axl* in 5TGM1s, ST2s or ST2-BMAds regardless of co-culture. GAS6 treatment in co-culture reduces AXL expression in both ST2 and 5TGM1s and induces 5TGM1 proliferation, but not in monoculture. GFP+DiD+ 5TGM1s were AXL+, and GFP+DiD- 5TGM1s were AXL-. In vivo, mice treated with the HFD had fewer GFP+DiI+ cells ($p=0.0254$), indicative of a reduction in dormant MM cells. GFP+DiI+ 5TGM1s were AXL+, and GFP+DiI- 5TGM1s were AXL-. HFD increased serum GAS6 ($p=0.0328$), which was decreased after tumour inoculation. Gene expression analysis of BM of MM-bearing mice revealed a significant increase in *axl* ($p=0.0469$) and *il6* ($p=0.0044$) but not in *gas6, adipoq, runx2* or *spp1* in the HFD group. Circulating AXL was significantly increased in MM-bearing mice, but not in mice receiving the HFD.

This study shows the complex regulation of dormancy-crosstalk in MM, with bidirectional AXL regulation in osteoblasts and MM cells and a change in MM dormancy in response to elevated adiposity, suggesting that adiposity may disrupt the dormant niche in MM.

### P15

### Single-cell functional biology in the tumour-bone microenvironment: Identification of individual prostate cancer cells with high metastatic potential

Mr Daniele Cotton, Mr Joseph Morgan, Mr Ahmed Alkhateeb, Dr Beatriz Gamez, Ms Danielle Whipp, Mrs Magdalena Hutchins, Professor James Edwards, Dr Srinivasa Rao, Professor Edmond Walsh, Professor Claire Edwards

University of Oxford, Oxford, UK

### Abstract

Bone is a frequent site of prostate cancer (PCa) metastasis. Understanding which cellular traits drive PCa bone metastasis is crucial for treatment. This study used microfluidic technology to (1) develop miniaturised multiplexed migration assays to identify rare, functionally unique cancer cell subpopulations and (2) assess individual PCa cell metastatic potential in bone microenvironment models. Cellular crosstalk was further examined in vivo using metastatic-niche labelling of the PCa-bone microenvironment.

Microfluidic assays assessed chemotactic PCa cell migration in monoculture and co-cultures, revealing subpopulations of ultra-migratory ‘super-spreader’ cells occurring in 9.4±3.3% of replicates, reflecting 0.02% of cells per assay. A subset of super-spreaders exhibited ‘polyaneuploid cancer cell state’ characteristics (a transient state implicated in treatment resistance), including senescence markers (β-galactosidase, γH2Ax foci) and increases in cell and nuclear size and perinuclear granularity. Individual super-spreader cells were isolated for continued analysis. Although initially non-proliferative, a PCa feeder bed restored superspreader proliferation after a 3–7-day quiescent period, reminiscent of the metastatic cascade.

Microfluidic single-cell picking revealed heterogeneity in PCa cell survival and proliferation when cultured with bone marrow stromal cells (BMSCs, HS-5 & HS-27A) and whole-murine marrow. Bone cells reduced heterogeneity in survival and proliferation relative to PCa controls ($p<0.001$-$0.0001$), suggesting microenvironment-clone interactions drive differential cell fate. PCa proliferation was higher in aged marrow than young.

To extend single-cell studies in vivo, we generated stable PC3 and C4-2b PCa cell lines expressing a secreted mCherry protein containing a modified lipid-permeable transactivator of transcription (TATk) peptide. In vitro, PCa-derived mCherry protein was taken up by surrounding BMSCs. SCID mice were inoculated intratibially with niche-labelling C4-2b or PC3 cells, and the proportion of mCherry-positive niche-labelled vs mCherry-negative distal stromal cells was quantified by flow cytometry.

C4-2b cells showed similar tumour burdens in central marrow and endosteal niches (11.1% vs. 10.1%), but niche-labelled stromal cells were more frequent in marrow (21.8% vs. 3.3%). In contrast, PC3 cells had higher tumour burdens in the endosteum than marrow (1.9% vs. 0.3%), with more niche-labelled cells in the endosteum (2.9% vs. 0.5%). These contrasting patterns indicate that the extent and nature of tumour-stromal cell communication differ between PCa cell lines, influencing how they establish and remodel their metastatic microenvironments in bone.

In conclusion, this study leveraged cutting-edge microfluidic technology to interrogate single-cell-level function in the bone microenvironment, identifying distinct PCa cells with high bone metastatic potential and highlighting the importance and complexity of tumour-bone crosstalk in bone metastatic prostate cancer.

### P16

### The pentose phosphate pathway increases prostate cancer tumour burden and osteolytic/osteoblastic bone disease in vivo

Dr Renee Ormsby, Miss Danielle Whipp, Mr Ahmed Alkhateeb, Mrs Magdalena Hutchins, Prof. Claire Edwards

University of Oxford, Oxford, UK

### Abstract

Bone metastatic prostate cancer (PCa) has a low survival rate of only 25%. Importantly, bone marrow stromal cells (BMSCs) support prostate cancer metastasis and bone disease. Altered metabolism is a key hallmark of cancer, and we have previously identified dysregulation of the pentose phosphate pathway (PPP) as a key player in PCa bone metastasis. The goal of the current study was to investigate the role of the PPP in the tumour-bone microenvironment by determining the effect of (i) different osteogenic lineage cells on expression of the rate-limiting enzyme G6PD, (ii) G6PD overexpression on PCa growth and associated bone disease in vivo and (iii) pharmacological inhibition of G6PD.

Here we show human HS5 and hFOB cells, but not mouse MC3T3 pre-osteoblast cells, upregulate G6PD and osteoprotegerin (OPG) in non-bone metastatic LNCaP cells. PCa cells and BMSCs were treated with polydatin (PD), a non-specific inhibitor of G6PD, or the specific inhibitor, G6PDi-1. Both drugs inhibited G6PD enzymatic activity in PC3 cells and decreased viability and proliferation (PD IC50:133 µM, G6PDi-1 IC50:104 µM, $p \leq 0.05$). PD induced apoptosis in PC3 cells and induced senescence in a subset of cells, as detected by morphological changes and increased γH2A.X and p21 expression. In comparison, G6PDi-1, decreased cell viability and proliferation in PC3 cells but did not induce apoptosis. G6PDi-1 also increased p21 expression, suggesting cell cycle arrest. HS5 cells showed no change in cell viability, and only a decrease in proliferation with the highest dose. Interestingly, G6PDi-1 decreased OPG expression in PCa cells, suggesting direct regulation of the RANKL/OPG signalling pathway by the PPP.

The ability of G6PD overexpression to drive tumour growth in bone remains unknown. G6PD was overexpressed (G6PD-OE) in non-bone metastatic LNCaP PCa cells. Following intratibial inoculation, increased PSA was detected, indicative of increased tumour growth in G6PD-OE LNCaP cells vs. control. MicroCT analysis showed altered morphology in mice inoculated with G6PD-OE LNCaP, with increased cortical bone compared to LNCaP control (n=6/group, $p<0.02$). The cortex also showed increased porosity, suggesting G6PD-OE LNCaP PCa cells induce a combined osteolytic and osteoblastic phenotype.

In summary, BMSCs support PCa metabolism by upregulating the PPP. Overexpression of G6PD alone is sufficient to promote tumour growth in bone in non-metastatic PCa cells, highlighting the importance of the PPP in bone metastasis. Furthermore, our studies with pharmacological inhibitors demonstrate the potential for therapeutic targeting of the PPP to combat PCa bone metastasis.

**P17**

Withdrawn

### P18

### Developing and validating *in vitro* and *ex vivo* models to study hormone regulation of osteocytes

Miss Rachel L. Wade[1], Dr. Donald R. Dunbar[1], Prof. Gurå T. Bergkvist[1,2], Prof. Colin Farquharson[1], Dr. Jennifer A. Fraser[1,2]

[1]Roslin Institute, Edinburgh, UK. [2]Royal (Dick) School of Veterinary Sciences, Edinburgh, UK

### Abstract

Osteocytes are the most abundant cells in the bone and orchestrate bone (re)modelling. Due to challenges in their isolation from bone, investigators have relied on the osteocyte-like cell lines, MLO-A5 and MLO-Y4, which represent post-osteoblast/pre-osteocyte and early osteocyte phenotypes, respectively. Despite both cell lines representing different stages in osteocyte cell differentiation, a comprehensive transcriptomic comparison has never been performed. RNA sequencing (Illumina, 150 paired end) generated >35 million read pairs per sample, and data were processed by the nf-core 'rnaseq' and 'differential abundance' workflows. Transcriptomic analysis identified 4,451 differentially expressed genes between MLO-A5 and MLO-Y4 cells. Late osteoblastic markers, alkaline phosphatase (*Alpl*) and collagen type 1 alpha 1 (*Col1a1*), were highly expressed in MLO-A5, whilst the early osteocyte markers, podoplanin (*Pdpn*) and gap junction alpha 1 (*Gja1*), were highly expressed in MLO-Y4 cells. Notably, early osteocyte marker, dentin matrix protein 1 (*Dmp1*), and late osteocyte markers, *Sost* and fibroblast growth factor receptor 23 (*Fgf23*) were not detected by RNA sequencing or qRT-PCR in MLO-A5 and MLO-Y4 cells. We are interested in the impact of hormone therapy on bone health and turnover; therefore, we assessed the expression of androgen and oestrogen receptors. Surprisingly, *Ar* and *Esr1&2* were lowly expressed in MLO-A5 and MLO-Y4 cells, suggesting they have limited utility to investigate hormone dependent control of osteocytes.

Isolated primary osteocytes cultured in 2D monolayers can lose features of mature osteocytes due to dedifferentiation. This could be driven by the absence of the mineralised matrix and the lacuna-canaliculi network, thus, an *ex vivo* osteocyte culture model was established. Murine long bones from 3.5-week-old mice were isolated; following the removal of the epiphyses and bone marrow, cortical bone was fragmented into 1-2 mm pieces (bone fragments) and serially digested with collagenase and EDTA to remove contaminating cells, leaving an enriched population of osteocytes within their mineralised matrix. After 96 hr in culture, qRT-PCR analysis indicated the osteocyte-containing bone fragments expressed late osteocyte markers, *Sost* and *Fgf23,* whilst immunohistochemical staining localised podoplanin and sclerostin to the osteocytes of the bone fragments. These preliminary results indicate that *ex vivo* culture of murine bone fragments provide a robust and physiologically relevant model to study mature osteocytes. Ongoing studies will characterise sex hormone receptor expression in the bone fragments to assess the suitability of this model to study hormone regulation of osteocytes and the potential role of osteocytes in prostate cancer metastases to the bone.

**P19**

Withdrawn

### P20

### Hypoxia increases tumour dormancy in the prostate-osteoblast niche

Miss Emma McGregor, Dr Renee Ormsby, Dr Young-Eun Park, Dr Edward O'Neill, Professor Claire Edwards

Nuffield Department of Surgical Sciences, University of Oxford, Oxford, UK

### Abstract

Prostate cancer (PCa) is the most common cancer in men in the UK, with a 5 year survival of ~33% in metastatic patients. Of these cases, 90% metastasise to the bone which leads to higher recurrence and poor survival. Disseminated PCa cells can survive in the bone marrow of patients for years without proliferating and causing any symptoms; they become dormant and evade treatment before eventually causing relapse and advanced cancer progression. The crucial mechanisms triggering tumour cells to become dormant upon encountering bone cells and importantly how dormant cells might be re-activated within the skeletal microenvironment remains unclear. Here, we investigate the effect of the bone microenvironment and hypoxia on PCa dormancy.

A co-culture model of PCa in bone has been used to assess dormancy through flow cytometric analysis of Vybrant DiD cell dye, allowing relative DiD intensity to be used to determine a dormant cell population. The PCa cell lines PC3 and LNCaP were labelled with DiD and directly co-cultured with HS5s, bone marrow stromal cells (BMSC), or MC3T3, pre-osteoblast cells. PC3 cells were found to have a 2-fold increase in the DiD-high dormant (or non-dividing) population in MC3T3 co-culture compared to alone - whilst LNCaP cells retained less DiD indicative of more proliferation when in MC3T3 co-culture and suggesting less dormancy potential in the presence of bone cells. To mimic the hypoxic environment of the intra-osseous niche, cells were co-cultured in 1% oxygen. Here, the percentage of DiD-high cells increased in MC3T3 co-culture, with PC3 showing a 19-fold increase and LNCaP a 4.34-fold increase (vs monoculture in 1% oxygen). Furthermore, increasing MC3T3 cell number prior to culture with PC3 and LNCaP cells increased the DiD-high dormant population under hypoxic conditions.

In contrast to MC3T3 pre-osteoblasts, co-culture with HS5 BMSCs induced a more dormant phenotype in LNCaPs, with a 100% increase in DiD-high population independent of hypoxia. PC3s were more proliferative when cultured with HS5 BMSCs.

In conclusion, BMSCs and pre-osteoblasts interact with PCa cells resulting in differential effects on dormancy, with hypoxic conditions seemingly increasing tumour dormancy in pre-osteoblast co-culture. Understanding this crosstalk in the PCa-bone dormancy niche will reveal new approaches to target PCa dormancy and ultimately reduce the incidence of PCa recurrence.

### P21

### Development of subject-specific finite element (FE) models of human vertebrae with and without lesion

Mr Eddie Shearman[1,2], Dr Rajdeep Ghosh[1,2], Prof Enrico Dall'Ara[1,3], Prof Damien Lacroix[1,2]

[1]Insigneo Institute, University of Sheffield, Sheffield, UK. [2]School of Mechanical, Aerospace and Civil Engineering, Sheffield, UK. [3]School of Medicine and Population Health, Sheffield, UK

### Abstract

### Introduction

Patients with vertebral metastases face a higher risk of spinal mechanical failure due to altered bone mineral density (BMD). Lytic lesions reduce BMD, blastic lesions increase it, and mixed lesions show both [1]. Currently, clinicians assess spinal stability using the Spinal Instability Neoplastic Score (SINS), but this qualitative approach lacks clear treatment recommendations [2]. Finite element (FE) models offer a more detailed understanding of spinal stability, helping clinicians advise patients.

### Method

Lytic, blastic, and mixed lesions were analysed alongside an adjacent control vertebra from ex vivo specimens. CT scans of each vertebra were segmented to create anatomical models, which were imported into Ansys and meshed with 1mm quadratic tetrahedron elements [3]. An apparent uniaxial, compressive strain of 1.9% was applied to the superior endplate, while the inferior endplate was fixed [3]. Heterogeneous, isotropic, non-linear (bilinear isotropic hardening) material properties were assigned to each vertebra. The evaluation included normalised failure load (reaction force of metastatic vertebra by control vertebra), equivalent stress, and total principal strains.

### Results

Table 1 shows the results for all three cases. The lytic vertebra had the highest stress (2.04 MPa). In the blastic case, the healthy vertebra experienced the higher stress, 1.82 times higher than the corresponding blastic vertebra. The highest strains were found in the lytic case. The highest normalised failure load was found in the blastic case. The highest strains were typically distributed in areas near the lesions and around the endplate of the vertebrae (Figure 1).

***Table 1:*** Normalised failure load, median minimum principal strains, and median equivalent stress.

| Donor | Lesion | Normalised Failure Load | Strain (με) | Stress (MPa) |
|---|---|---|---|---|
| A | Healthy | 1.1 | 9850 | 1.73 |
| | Lytic | | 9540 | 2.04 |
| B | Healthy | 1.99 | 7610 | 0.14 |
| | Blastic | | 4170 | 0.42 |

| | | | | |
|---|---|---|---|---|
| C | Healthy | 0.87 | 3970 | 1.07 |
| | Mixed | | 5900 | 0.87 |

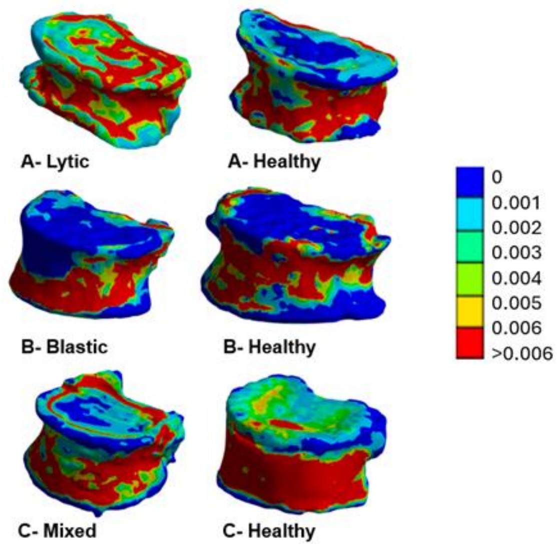

***Figure 1:*** Distribution of total principal strains.

**Conclusion**

These FE models are a good insight into how the mechanical stability of metastatic vertebrae is affected. However the complexity of the problem with each case having different size, severity and location of lesions means it's important that more simulations are performed to get more refined results.

**Acknowledgements**

METASTRA (EU H2022 grant ID 101080135; UK Horizon Europe Guarantee Extension ID: 10075325) and Metaspine (MSCA-IF-EF-ST, 832430/2018) projects.

**References**

[1]- Lee, S. (2014). Bone metastases. Canadian Cancer Society.
[2]- Palanca, et al. (2023). 173, p.116814.
[3]- Costa et al. (2019). JMBBM, 98, pp.268–290.

### P22

### Osteoblasts increase APOBEC3B and DNA damage in prostate cancer cells: a new mechanism underlying genomic instability in prostate cancer bone metastasis

Mr Ahmed Alkhateeb[1], Dr Renee Ormsby[2], Dr Ninu Poulose[2], Mr Robert Crickley[1], Prof Joanna Hester[2], Prof Fadi Issa[2], Prof Ian G. Mills[2], Dr Srinivasa R. Rao[2], Prof Colin R. Goding[1], Prof Claire M. Edwards[3]

[1]Nuffield Department of Medicine, University of Oxford, Oxford, UK. [2]Nuffield Department of Surgical Sciences, University of Oxford, Oxford, UK. [3]Nuffield Department of Orthopaedics, Rheumatology and Musculoskeletal Sciences, Nuffield Department of Surgical Sciences, University of Oxford, Oxford, UK

### Abstract

Metastatic prostate cancer (mPCa) is a highly heterogeneous disease with a strong propensity to metastasize to bone, where the tumour microenvironment plays a crucial role in progression, therapy resistance and development of the associated bone disease. APOBEC3B (A3B), a DNA cytosine deaminase, drives mutagenesis and genomic instability in multiple cancers, including mPCa. Elevated A3B expression is associated with increased tumor heterogeneity and resistance to androgen deprivation therapy (ADT) and other systemic treatments, suggesting a role in PCa progression. However, the regulatory mechanisms controlling A3B expression in bone-metastatic PCa remain poorly understood. RANKL, a key mediator of osteoclast activation and bone remodelling, is highly expressed in the mPCa bone microenvironment, driving cancer-induced bone disease. NFkB is known to regulate A3B, raising the possibility that RANKL may induce A3B expression in PCa cells, and so impact DNA damage and genomic instability. Here, we investigate the effects of A3B overexpression in mPCa and explore its regulation by RANKL and osteoblast co-culture. A doxycycline (Dox)-inducible A3B-HA expression system was established in mPCa cell lines to evaluate A3B-induced DNA damage. To investigate A3B-driven molecular changes, bulk RNA sequencing (RNA-seq) was performed on LNCaP cells expressing Dox-inducible A3B-HA, followed by differential expression analysis. A3B regulation was assessed by treating PCa cells with recombinant RANKL or co-culturing LNCaP cells with HfOB osteoblasts ± osteoprotegerin (OPG). Dox-induction of A3B resulted in a 2-fold increase in the DNA damage marker γH2AX in DU145 and LNCaP cells ($p < 0.05$) cells, as measured by western blot and immunofluorescence. RNA-seq analysis of A3B-overexpressing LNCaP cells revealed significant transcriptomic changes, including upregulation of DNA damage response (DDR) pathways, suggesting that A3B contributes to genomic instability in mPCa. Activation of NFkB by PMA resulted in an increase in A3B expression. RANKL treatment or coculture with osteoblasts significantly upregulated A3B expression and γH2AX in LNCaP cells ($p < 0.05$). The addition of OPG to PCa/osteoblast cocultures reduced the increase in A3B. ATF4 is known to regulate the RANKL signalling pathway. Inducible expression of ATF4 in PCa cells was found to increase A3B expression and DNA damage. Our findings demonstrate that A3B overexpression induces DNA damage and alters transcriptomic profiles linked to genomic instability in metastatic prostate cancer. We show that the bone microenvironment, specifically osteoblast-derived RANKL, is a key driver of A3B expression and DNA damage, with ATF4 potentially mediating this effect. These results demonstrate novel regulatory crosstalk in which the bone microenvironment can increase DNA damage in PCa, highlighting new fundamental mechanisms underlying disease progression and bone metastasis.

### P23

### Osteoclastic resorption pit depth, volume and roughness are substrate-dependent in articular calcified cartilage and bone

Mr. Khizar Hayat[1,2], Dr. Aimee Colbath[2], Dr. Mason Dean[1], Dr. Michael Doube[1]

[1]City University of Hong Kong, Hong Kong, Hong Kong. [2]Cornell University, Ithaca, New York, USA

### Abstract

**Introduction:** Subchondral bone advancement occurs via remodelling of articular calcified cartilage (ACC) into bone, by osteo(chondro)clastic resorption and osteoblastic deposition. Although resorption has been studied extensively on bone and dentine as substrates, to our knowledge, osteoclastic activity on calcified cartilage has not been investigated in vitro.

**Objective:** We investigated osteoclastic activity on ACC to document the pattern of resorption (volume, shape, depth and roughness) compared to cortical bone.

**Methodology:** Haematopoietic stem cells from equine bone marrow were differentiated into osteoclasts and seeded onto thin mineralised slices (~200 um) comprising ACC and bone. Osteochondral slices were prepared in two different orientations so that osteoclasts could work parallel (ACC-H) or orthogonal (ACC-S) to collagen-II fibrils. Osteoclast differentiation media (α-MEM, 10% FBS, 50 ng/ mL M-CSF and RANKL) was used (changed every three days), and the presence of osteoclasts was validated with tartrate-resistant acid phosphatase (TRAP) stain. After 21 days, slices were imaged with reflective confocal microscopy. A Kruskal-Wallis test was used to compare different groups with a 95% confidence interval.

**Results:** The volume of resorption pits was significantly greater ($p=0.012$) in ACC-H (2295 ± 1456 $\mu m^3$) than bone (859 ± 1399 $\mu m^3$). Resorption pits were significantly deeper in ACC-H (2.5 ± 0.18 µm) and ACC-S (2.1 ± 0.14 µm) than bone (1.2 ± 0.15 µm) ($p<0.0001$, $p=0.023$, respectively). The aspect ratio (depth/width) was significantly greater ($p=0.0022$) in ACC-H (0.94 ± 0.05) than ACC-S (0.67 ± 0.07). Average pit roughness (Ra = 1 ℓr ∫0 |Z(x)|dx) was greatest in ACC-H (2.4 ± 0.17 µm), followed by ACC-S (1.7 ± 0.11 µm) and was significantly lower in bone (1.1 ± 0.11 µm) ($p<0.0412$).

**Discussion:** The orientation and type of the substrate significantly affect osteoclastic activity. The rate of osteoclastic resorption is significantly higher in ACC than in bone, leaving behind a rougher surface. Resorption pit roughness might be due to the presence of unresorbed collagen fibrils. Collagen fibrils retained in the resorption pit might affect the ACC-to-bone transition and bone-cartilage bonding at the osteochondral cement line. Further, the difference in the shape and depth of the resorption pits suggests that osteoclastic activity is dependent upon collagen-II fibril directionality (end-on vs side-on).

**Significance:** Osteoclastic activity is dependent upon tissue type, and orientation of collagen in ACC, consistent with other mineralised matrices. ACC resorption pit roughness could be instrumental to the formation of the strong bond between articular cartilage and bone.

### P24

### The Role of Hypergastrinemia in Proton Pump Inhibitor-Induced Bone Fractures

Miss Aine Pears[1], Dr Malcolm Boyce[2], Dr Joanna Seiffert[2], Dr Emily Corlett[2], Dr Alan J. Stewart[3], Dr Gerry McLachlan[1], Professor Colin Farquharson[1]

[1]The Roslin Institute, University of Edinburgh, Edinburgh, UK. [2]Trio Medicines Ltd, London, UK. [3]School of Medicine, St Andrews, St Andrews, Fife, UK

### Abstract

Proton pump inhibitors (PPIs) are amongst the most prescribed medicines in the world with more than 70 million prescriptions issued during 2023 in the UK alone. The safety records of PPIs are generally favourable but epidemiological studies suggest an increase in fracture risk associated with chronic PPI use. The underlying mechanisms of this association have not yet been elucidated. The most commonly accepted explanation is that PPIs cause calcium malabsorption by reducing stomach acid, leading to hyperparathyroidism and increased osteoclastic bone resorption. However, these data are inconsistent and often disputed. In this study, alternative mechanisms for the increased fracture risk were explored, with the overall aim of ascertaining whether this may be mediated by hypergastrinemia; a consequence of chronic PPI use.

It is unknown if osteoblasts express gastrin receptors. Therefore, as an initial step in this investigation, primary human osteoblasts were evaluated for functional gastrin receptors. Primary human osteoblasts were analysed by RT-qPCR, western blot, and immunocytochemistry, revealing the presence of the gastrin/cholecystokinin-2 (CCK-2) receptor. Further to this, the functionality of the receptor was assessed, where gastrin induced phosphorylation of ERK 1/2 and JNK but not p38 or AKT, suggesting gastrin utilises the MAPK intracellular signalling pathway. Gastrin treatment reduced the expression of genes associated with osteoblast differentiation and mineralisation; collagen type 1 (*COL1A1*), $p<0.01$; osteopontin (*SPP1*), $p<0.001$; osteocalcin (*BGLAP*), $p<0.05$; PHOSPHO1, $p<0.001$ and osteonectin (*SPARC*), $p<0.05$. Additionally, gastrin inhibited proliferation (0.1 – 10nM, $p<0.05$) and reduced alizarin red staining (0.01 – 10nM, $p<0.01$). In contrast, alkaline phosphatase activity was increased by gastrin (control media: 53.6 µmol/L/min), 10µM gastrin; 87.7 µmol/L/min), $p<0.001$) and was inversely correlated with matrix mineralisation.

These data establish the presence and functionality of osteoblast gastrin receptors raising the possibility that the elevated gastrin levels that occur as a consequence of PPI use may act directly on osteoblasts to impair their function and ultimately lead to deficits in bone formation.

I*n vitro* studies will assess the ability of a gastrin receptor antagonist to block the adverse effects of gastrin on human osteoblast gene expression, proliferation, differentiation and matrix mineralisation. Also, a pre-clinical rat model of PPI-induced hypergastrinemia on bone has been developed to examine the potential of a gastrin receptor antagonist to prevent PPI induced bone loss.

### P25

### mTOR signalling is an intrinsic driver of osteoblast regulation in bone development during adolescent idiopathic scoliosis (AIS)

Miss Ellie Northall[1], Dr Thomas Nicholson[1], Dr Jon Hazeldine[1], Professor Liam Grover[2], Professor Helen McGettrick[1], Mr Matt Newton Ede[3], Dr Amy Naylor[1], Professor Simon Jones[1]

[1]Department of Inflammation and Ageing, MRC Centre for Musculoskeletal Ageing Research, University of Birmingham, Birmingham, UK. [2]School of Chemical Engineering, University of Birmingham, Birmingham, UK. [3]Royal Orthopaedic Hospital NHS Trust, Birmingham, UK, Birmingham, UK

### Abstract

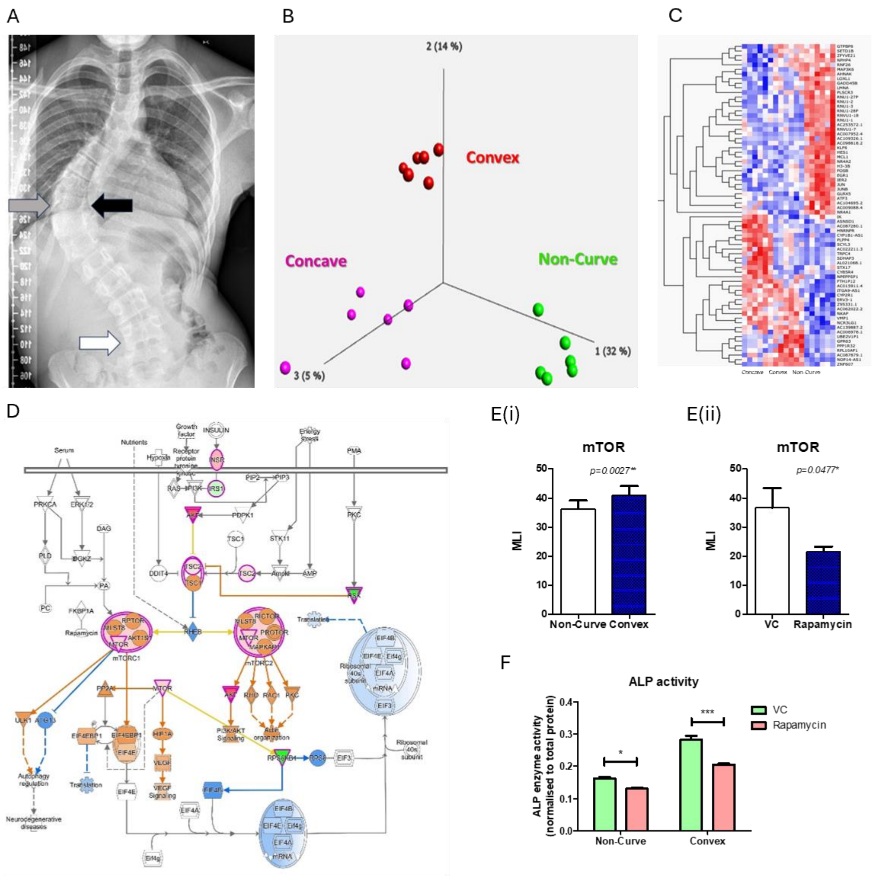

**(A)** Tissue collection sites of AIS patients (n=22) including at the curve apex (grey/black arrows), and non-curve (white arrow) control tissue. **(B-C)** Principal component analysis of bulk RNA-Seq reveals distinct clustering by tissue site, independent of other patient characteristics. Dysregulated transcriptome between sites evidenced in the heat map. **(D)** The mTOR pathway is dysregulated between non-curve and convex cells (IPA analysis). **(E) i.** mTOR protein expression is significantly increased in convex osteoblasts compared to non-curve. **ii.** Rapamycin treatment decreases mTOR protein expression of convex osteoblasts compared to vehicle control (VC) (MLI: Median luminescence intensity). **(F)** An mTOR inhibitor (rapamycin) inhibits alkaline phosphatase activity compared to vehicle control.

**BACKGROUND:** Adolescent Idiopathic Scoliosis (AIS) affects 2–3% of children aged 10–18 and is characterized by lateral spinal curvature without a known cause. Severity varies, with mild cases requiring observation and more pronounced curvatures necessitating bracing or surgery, such as spinal fusion, vertebral body tethering, or growing rod implantation. Previously, we reported that AIS spinal osteoblasts at the curve apex exhibit dysregulated metabolic and transcriptomic phenotypes, compared to osteoblasts from non-curve spinal tissue from the same patients[1].

**AIMS:** To identify dysregulated signalling pathways in spinal osteoblasts at the curve apex in AIS patients and evaluate the potential of pharmacological agents to restore their function and phenotype to resemble non-curve osteoblasts.

**METHODS:** Displaced spinal bone tissue was collected intraoperatively from the curve apex (concave and convex) and non-curve regions of 22 AIS patient during elective orthopaedic rod implantation corrective surgery (19/WM/0083). Bulk RNA sequencing (n=6 patients, paired samples) identified candidate pathways and targets using Ingenuity Pathway Analysis (IPA, Qiagen). Osteoblast function was assessed by alkaline phosphatase (ALP) activity, osteoprotegrin (OPG) secretion, and mineral deposition (alizarin red staining). The mTOR pathway was identified as a key target, and its modulation was tested using rapamycin (1 nM). Intrinsic mTOR pathway differences between curve and non-curve osteoblasts were evaluated at transcriptomic (qPCR) and protein (Luminex) levels.

**RESULTS:** RNA-Seq revealed distinct transcriptomic profiles between curve apex and non-curve osteoblasts. IPA identified dysregulated canonical pathways including mTOR signalling and identified candidate upstream regulators as potential therapeutic targets. Rapamycin, an mTOR inhibitor, normalized osteoblast phenotype by reducing ALP activity, OPG secretion, and mineralization in cells from curved regions, aligning them with non-curve osteoblast function.

**CONCLUSION:** While surgical intervention remains the primary treatment for severe AIS, it carries significant physical and psychological burdens, including the risk of surgical complications, prolonged recovery, and uncertain long-term outcomes. A pharmaceutical approach offers a non-invasive alternative, reducing procedural risks and psychological distress while improving accessibility. Osteoblasts from the curve apex show dysregulated skeletal development pathways, notably mTOR. Modulating these pathways with repurposed drugs like rapamycin presents a promising avenue for regulating abnormal bone development in scoliosis.

**REFERENCES**: 1. Pearson, M. J., Philp, A. M., Haq, H., Cooke, M. E., Nicholson, T., Grover, L. M., Newton Ede, M., & Jones, S. W. (2019). Evidence of Intrinsic Impairment of Osteoblast Phenotype at the Curve Apex in Girls With Adolescent Idiopathic Scoliosis. Spine deformity, 7(4), 533–542. https://doi.org/10.1016/j.jspd.2018.11.016

### P26

### FGFR1 and ERK1/2 Signalling Mediate Extracellular Phosphate Sensing in Osteoblasts to Regulate Matrix Mineralisation

Dr Soher Jayash, Mr Thomas Duff, Mr Qaisar Tanveer, Prof Colin Farquharson

The Roslin Institute, University of Edinburgh, Edinburgh, UK

### Abstract

The regulation of phosphate (Pi) availability is essential for skeletal biomineralisation, involving both systemic and local mechanisms. However, the relative contributions of extracellular Pi and locally generated Pi remain unclear. Osteoblasts may act as Pi sensors, modulating phosphatase activity to fine-tune the mineralisation process. This study examined how extracellular Pi influences the expression of tissue-nonspecific alkaline phosphatase (TNAP) and PHOSPHO1, two key enzymes in bone matrix mineralisation. Human primary osteoblasts were cultured in varying Pi concentrations (0, 1 mM, 3 mM, and 5 mM). Matrix mineralisation was assessed using alizarin red staining, while the expression of PHOSPHO1, ALPL, SLC20A1/PiT1, and SLC20A2/PiT2 was analysed by RT-qPCR and immunoblotting. Intracellular signalling pathways were evaluated through immunoblotting for total and phosphorylated ERK1/2, AKT, p38, and JNK1/2. Increased extracellular Pi levels downregulated PHOSPHO1 and TNAP expression at both mRNA and protein levels, alongside reduced expression of PiT1 and PiT2. Pi exposure selectively enhanced ERK1/2 phosphorylation, with no significant effects on AKT, JNK1/2, or p38. Inhibition of PiT1/PiT2 using Foscarnet or MEK1/2 using UO126 prevented Pi-induced suppression of PHOSPHO1 and ALPL expression. Additionally, extracellular Pi activated FGFR1 signalling by phosphorylating FRS2α, an effect blocked by Foscarnet. Inhibition of FGFR signalling with PD173074 abolished ERK1/2 phosphorylation and reversed the Pi-induced downregulation of ALPL and PHOSPHO1 expression. Finally, the promotion of osteoblast matrix mineralisation by extracellular Pi required both type III Na-Pi co-transporters and FGFR signalling. These findings reveal a critical interplay between FGFR1 and Pi transporters in osteoblasts, highlighting a complex regulatory mechanism underlying physiological bone mineralisation. Understanding this crosstalk provides new insights into the molecular control of skeletal health and disease.

### P27

### PAR2 Deletion Alters Gene Expression Linked to Energy Metabolism in Female Mice Bone Cells

Mr Alexandros Lamprou, Prof. Carl Goodyear, Dr. Carmen Huesa

University of Glasgow, Glasgow, UK

### Abstract

Osteoarthritis (OA), the most prevalent form of arthritis, affects millions worldwide, primarily driven by aging, obesity, and genetic predisposition. Within the joint, bone has a central role in driving osteoarthritic disease. Proteinase activated receptor 2 (PAR2) is central to osteoblast biology, cartilage health and OA progression. PAR2 is a promising therapeutic target. This study explored PAR2's role in osteoblast lineage, focusing on gene expression changes influenced by PAR2 deletion in young adult and aged mice. Long bones were isolated from wild-type (WT) and PAR2-knockout (PAR2−/−), 3 (young) and 12 (old) month-old female mice . (WT-young (n=3), WT-old (n=6), KO-young (n=4), and KO-old (n=5)). Bones were homogenised to extract RNA after flushing the bone marrow. RNA was sent for sequencing using Illumina's NovaSeq-X series. Libraries were prepared through poly(A)-tail enrichment, and RNA quality was assessed using the Falcon II system. Differential expression (DE) analysis with DESeq2 identified genes and pathways affected by PAR2 deletion (log2FC > 1, p.adj < 0.05). Key findings were validated by qPCR. Principal Component Analysis (PCA) revealed clustering patterns between WT and PAR2−/−, with clustering influenced by age. The PAR2 gene expression was significantly higher in WT-old vs WT-young mice ($p < 0.05$). The DE analysis between all the WT and PAR2-/- identified 15 genes impacted by PAR2 deletion, with 14 genes being significant downregulated, such as PCK1 (p.adj = 0.02), crucial for glucose homeostasis, and PLIN1 (p.adj = 0.002), involved in adipocyte differentiation. These were of greater significance in the older mice. Specific analysis of older mice identified additional significantly different genes, such as KLB (*p*.adj = 0.048), which is involved in fibroblast growth factor binding activity. Pathway analysis of the downregulated genes revealed the "Regulation of Lipolysis in Adipocytes" (p.adj = 0.01) and "PPAR Signalling Pathway" (p.adj = 0.01) as the most significantly affected pathways. A closely connected network of interactions was identified among key downregulated genes involved in energy metabolism functions and pathways. Results indicated PAR2 is not detectable in young adult bone but shows increased expression in old adult bone. This study underscores the pivotal role of PAR2 in modulating key metabolic pathways within bone cells, such as lipolysis and gluconeogenesis, which can have an important impact in the osteoblast lineage's phenotype. Further research into PAR2's regulation and its effects on osteoblast differentiation and function based on energy metabolism, could provide valuable insights into novel treatments for OA and related conditions.

### P28

### Charting Tissue-specific Ageing-related Transcriptomic Changes Across the Lifecourse

Dr Jinsen Lu[1], Dr Srinivasa Rao[1], Miss Eleanor Platt[2], Dr Lucy Frost[2], Dr Tiffany-Jane Allen[2], Dr Gayle Marshall[2], Prof James Edwards[1]

[1]Univeristy of Oxford, Oxford, UK. [2]Medicines Discovery Catapult, Alderly Edge, UK

**Abstract**

Ageing is inextricably linked to changes in normal physiology. These present as recognisable features of 'getting old', in worst cases as a collection of multimorbidities including frailty, bone loss, neurodegeneration. An improved understanding of key ageing drivers and their role in disease pathogenesis, is essential to better predict and pre-empt disease occurrence, characterise and develop new treatment targets and preserve healthspan.

We used the Nanostring GeoMX platform to assess local transcriptomic changes across the healthy murine lifecourse (F, 3 or 22mth), assessing and validating different regions of interest (ROI) within multiple tissues (eg. brain, lung, heart).

A significant tissue-specific alteration in gene expression was seen between maturity and old age. Interestingly, not all organs aged at the same (transcriptomic) rate, where different tissues display vastly different numbers of gene alterations to others. The largest change was observed in gastrointestinal tissues (ROI=small intestine, epithelia and crypts) where 841 differentially expressed genes (DEGs) were recorded, spleen (616 DEGs), and liver (468 DEGs). However, far fewer significant DEGs were recorded between young and old brain (16), and lung (12). Principal component analysis (PCA) revealed the most variable genes across tissues with ageing. This showed commonality in DEGs across certain sites, where similar gene signatures (Arf5, Vamp8, Ogdh, Glrx) were elevated in brain, kidney, stroma, lung, skin ($p<0.05$). DEGs at small intestine, spleen and liver showed a far greater degree of tissue-specificity. A pathway analysis using top 100 DEGs, GO and KEGG platforms, showed significant changes (<p adj 3e-04) in gene groups linked to biological processes (NAD-processing, cellular respiration/metabolism), molecular function (cytoskeletal activity), and cellular components (actin projections, microvilli, myelin). When cross-referenced with the SenMayo gene panel (developed to identify transcriptomic signatures of senescence across multiple organ systems), 20 significant gene changes were seen, but only at ROI from liver, intestine, kidney (increased Mif, Egfr, Vegfa, Ctsb; decreased Cd9, Jun, Igfbp4, Angptl4 in liver, with opposite effects in intestine, kidney). Our analysis was validated against the Mouse Ageing Cell atlas (Zhang et al), with 100% positive mapping ratio in neuronal tissues, >80% in liver, kidney, and >60% in muscle, skin.

These findings demonstrate shared ageing-linked genes common to several organs, alter expression congruently with increasing age, and impact key 'hallmarks of ageing' mechanisms (eg VAMP8-autophagy, Ogdh-NAD metabolism, Glrx-mitochondrial biogenesis), and to a lesser degree, in an organ-specific manner where such sites might be more sensitive to the accumulation of senescent cells with increasing age.

### Graphic Abstract

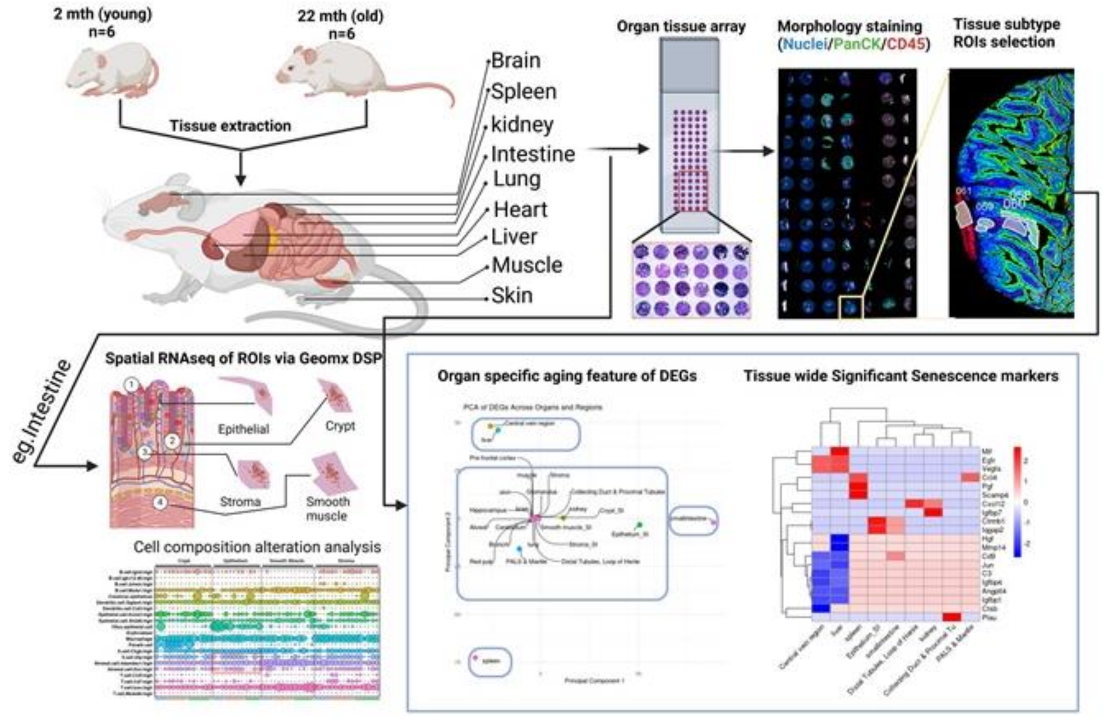

**P29** (Also presented as an Oral Communication in Clinical Cases Session)

### Autosomal Dominant Hypophosphatemic Rickets (ADHR) and Its Management in Pregnancy

Dr Fiona Vaz[1], Professor Emma Duncan[1,2]

[1]Guy's and St Thomas' NHS Foundation Trust, London, UK. [2]King's College London, London, UK

### Abstract

**Autosomal Dominant Hypophosphatemic Rickets (ADHR)** is a rare disorder caused by heterozygous mutations in *FGF23*, making the protein resistant to cleavage. Excess FGF23 causes renal phosphate wasting, hypophosphatemia, low 1,25$(OH)_2D_3$, secondary hyperparathyroidism, and osteomalacia, ADHR manifests clinically with bone pain, muscle weakness, skeletal deformities and fractures. Iron deficiency worsens ADHR by upregulating *FGF23* expression, particularly evident during periods of increased iron demand including pregnancy. Conversely, iron infusions (particularly iron carboxymaltose) can further lower phosphate levels.

We describe the pregnancies of two sisters with ADHR living out of area. The eldest, currently pregnant with her third child, has ADHR alongside an eating disorder, poor medication compliance and chronic iron deficiency. During her first pregnancy, managed elsewhere, an iron infusion resulted severe hypophosphataemia (0.19mmol/L) requiring emergency intravenous replacement. An emergency caesarean was performed at 36 weeks for foetal growth restriction.

Her second pregnancy, managed at our clinic, was also complicated. Stress fractures in her tibia and metatarsals were diagnosed in the second trimester (noting here that ALP was elevated pre-pregnancy; placental-ALP production complicates interpretation during pregnancy). She required high doses of phosphate (6-8 Sandoz tablets/day), alfacalcidol (8 mcg/day), and oral iron (400 mg ferrous sulphate/day), yet serum phosphate, adjusted-calcium and ferritin remained low (0.3-0.4 mmol/L, 2.1 mmol/L, 5.3 µg/L respectively). Nonetheless, the baby grew well and was delivered close to term, again by caesarean.

She recently represented at four weeks' gestation, with phosphate 0.35mmol/L, ferritin 17µg/L, and 25(OH)D 22nmol/L and acknowledged recent poor compliance with replacement. Treatment has been restarted.

The younger sister, also affected, was recently pregnant. She first presented at 11 weeks' gestation with hypophosphatemia (0.6 mmol/L), low ferritin (16 µg/L), and vitamin D of 41 nmol/L. She was advised to take phosphate, alfacalcidol, cholecalciferol and a combined iron/folate preparation (Fefol). However, by 27 weeks, she had not started Fefol and other compliance was tenuous. She reported unexplained 'blackouts' and with phosphate levels 0.3mmol/L, was hospitalised for intravenous replacement. Her baby was delivered at term.

These cases illustrate the challenges of managing ADHR in pregnancy, particularly in the context of nutritional deficiencies, eating disorders and iron deficiency, all of which exacerbate phosphate wasting. They also highlight the complexities of coordinating care across multiple clinical teams.

Burosumab a monoclonal antibody targeting FGF23, is licensed for X-linked hypophosphatemic rickets in (non-pregnant) adults but not ADHR. The long-term bone health and future pregnancy outcomes for this family remain uncertain.

**P30** (Also presented as an Oral Communication in Clinical Cases Session)

### Chronic Nonbacterial Osteitis with Clavicular Expansion and Thrombotic Complications: A Case Report

Ms Nina Thomson[1], Dr Jack Leese[2], Dr James Bott[3], Dr Barbara Hauser[2]

[1]Edinburgh Medical School, Edinburgh, UK. [2]NHS Lothian, Rheumatology, Edinburgh, UK. [3]NHS Lothian, Radiology, Edinburgh, UK

### Abstract

### Background

Chronic Nonbacterial Osteitis (CNO) is a rare autoinflammatory bone disorder characterized by sterile bone inflammation. Chronic inflammation can lead to sclerosis, hyperostosis and soft tissue ossification. Beyond bone involvement, patients may present with inflammatory arthritis, spondyloarthritis, palmoplantar pustulosis (PPP) and psoriasis.

### Case Presentation

A 30-year-old Scandinavian male initially presented with a rash affecting the palms and soles, diagnosed as PPP, and was treated with topical emollients and intermittent NSAIDs. Over the following years, he developed progressive sternoclavicular discomfort, back pain, and stiffness, though these symptoms were not formally evaluated.

At age 40, he presented with left upper arm swelling. Investigations revealed an upper limb deep vein thrombosis (DVT) due to thoracic outlet obstruction caused by left-sided clavicular expansion. Computed tomography (CT) and venography confirmed subclavian vein compression due to extensive clavicular cortical overgrowth (Fig 1a). A chest CT demonstrated clavicular sclerosis and cortical expansion consistent with CNO (Fig 1b), as well as thoracic spine syndesmophytes suggestive of axial spondyloarthritis (axSpA). Laboratory investigations showed persistently elevated inflammatory markers (CRP: 30–52 mg/L) and a negative HLA-B27 status. The patient was started on long-term anticoagulation (Apixaban 5mg bd) for DVT management. Rheumatology assessment confirmed a diagnosis of CNO/SAPHO, with further evaluation planned for potential anti-TNF therapy to control osteitis, PPP and possible axSpA.

### Discussion

The presence of PPP, sternoclavicular discomfort and swelling, and inflammatory back stiffness, combined with chronic inflammation should have facilitated an earlier diagnosis of CNO. Delayed recognition likely contributed to progressive bone overgrowth, vascular compression, and thrombotic complications. Current CNO guidelines[1] recommend parenteral bisphosphonates as first-line therapy after an inadequate NSAID response. However, this case highlights the need for individualized treatment, as TNF inhibitors (TNFi) may be more effective due to their potential benefit in both PPP and axSpA. The ability of TNFi or bisphosphonates to prevent clavicular expansion remains uncertain. TNFi may also reduce DVT risk by controlling systemic inflammation.

**Conclusion**

This case underscores the morbidity associated with delayed CNO diagnosis, including rare complications such as thoracic outlet obstruction and DVT. Early recognition of hallmark clinical features is essential to prevent irreversible complications.

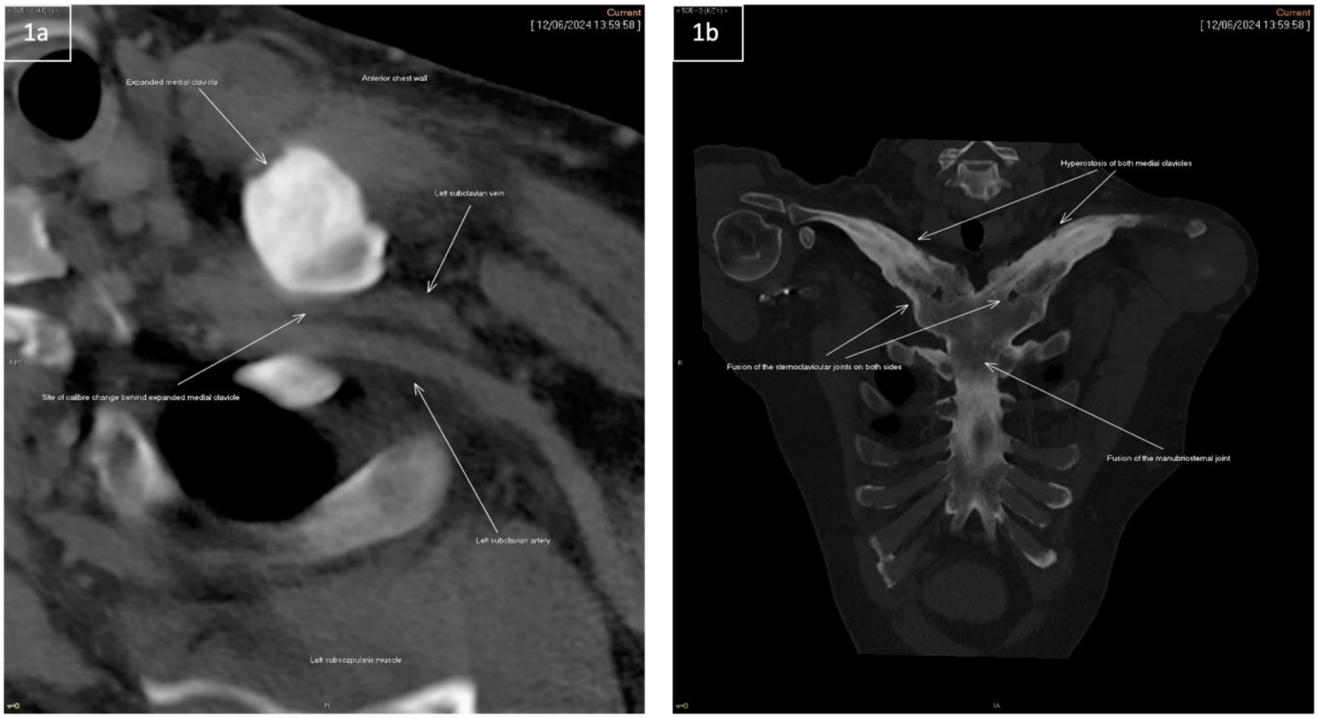

**Figure 1a:** Axial Contrast-Enhanced CT of the Thoracic Outlet shows expansile cortical overgrowth of the left medial clavicle, compressing the left subclavian vein, resulting in a caliber change indicative of venous obstruction. Figure 1b Coronal CT image reveals bilateral clavicular hyperostosis, with fusion of the sternoclavicular joints and sclerosis of the manubriosternal joint, indicative of CNO.

References:

1. Winter EM *et al* ARD Published Online First: 28 November 2024. doi: 10.1136/ard-2024-226446

### P31

### A Rare Case of Skeletal Fluorosis with Increased Bone Density in an Indian Man from Punjab Presenting at a Tertiary Care Center in the UK

Dr Thejashwini Mahadevaswamy, Mr Mateusz Wawaczyk, Dr Faizanur Rahman, Dr Prashanth Patel, Dr Mohamed Saeed

University Hospitals of Leicester, Leicester, UK

### Abstract

Fluoride is essential for bone formation, but prolonged high exposure can cause dental and skeletal fluorosis. Skeletal fluorosis leads to brittle bones and fractures, common in regions like India, China, Africa, and Mexico due to contaminated groundwater. In developed countries like the UK, it is rare and usually linked to chronic exposure from industrial sources or excessive fluoride-containing products.

We report a case of a 66-year-old Indian male referred after a pelvic X-ray, following a fall, which showed increased bone density. He had mild low back pain, lower limb weakness for a year, and a history of hypertension, coronary artery disease, uncontrolled type 2 diabetes, and hypothyroidism. He also had discoloration of his front two teeth. He used Listerine mouthwash, drank three cups of black tea daily, and migrated from Punjab, a fluorosis-endemic region, at age seven.

Upon further evaluation, the patient's muscle weakness was severe, and he described it as having "lost his buttocks in a year." Although diabetic amyotrophy was considered as a differential diagnosis, nerve conduction studies showed no peripheral nerve disorder and MRI spine revealed slight suspicion of S1 nerve root irritability but no other features to suggest radiculopathy.

He presented during the COVID-19 pandemic, and investigations were conducted irregularly. Alkaline phosphatase was 144 IU/L (30–130) while calcium, phosphate, magnesium, and PTH were normal. Vitamin D was 40 nmol/L, HbA1c was 11.2%, and TSH was 10 mIU/L (0.30–5.00). His gender hormones, VitaminA and Mast cell tryptase levels were normal. Hep C was negative. 24-hour urine fluoride was elevated at 2.93 mg/24hrs (0.00–1.64), improving over time. Serum fluoride was 156 µg/L (0–50) three years later.

His isotope bone scan showed diffuse increased uptake with no focal lesions. A bone density scan three years later showed Z-scores: L1-L4 +10.9, total left femur +3.1, total right femur +2.8, and left and right femoral necks +2.2 and +2.5 respectively. Radial bone density is pending.

Fluorosis is known to increase trabecular bone density as seen here. Although he migrated at age 7, being from Punjab (endemic region), there is possibility he may have been exposed during visits. He stopped using fluoridated mouthwash, toothpaste and his urine excretion of fluoride look better over subsequent years. This case highlights the importance of considering historical fluoride exposure in the diagnosis and management of musculoskeletal conditions particularly in migrants, with ongoing evaluations to better understand the long-term impact on bone health.

### P32

### Getting Weaker and Breaking Up, Followed by Cure. An Interesting Case of Oncogenic Osteomalacia and a Patient Safety Audit on Hypophosphatemia

Dr Kapil Kumar Garg[1], Ms Sukhjinder Moore[2], Dr Paul Byrne[2]

[1]Basildon University Hospital, Mid & South Essex NHS Foundation Trust, Basildon, UK. [2]Colchester General Hospital, East Suffolk and North Essex NHS Foundation Trust, Colchester, UK

### Abstract

### Background:

Oncogenic Osteomalacia or Tumour Induced Osteomalacia (TIO) is a paraneoplastic-syndrome characterised by bone pain, fractures and muscle weakness. It is caused by tumoral-overproduction of fibroblast growth factor 23 (FGF-23) producing hypophosphatemia and Osteomalacia. The tumour is usually benign and runs an indolent course.

### Case Presentation:

A 39 years previously fit male presented with heel pain and declining general strength for 2-years. There was no family history of metabolic bone disease. He sustained three fragility fractures of metatarsals over 2-years. Bloods showed raised ALP 209(30-130), normal calcium, normal PTH and persistently low phosphate of 0.29mmol/L(0.8-1.5). Fasting TMP/GFR (Tubular Maximum Phosphate Reabsorption per litre GFR) was low 0.36mmol/L (0.9-1.35), and fractional excretion of phosphate (random urine) at 28.8%. This was suggestive of renal phosphate wasting. Patient had low 1,25 (OH)2 Vit D at 27pmol/L (43-144) and optimal 25-(OH)-Vitamin D levels (reduced 1-alpha hydroxylase activity). Fibroblast Growth Factor-23 (FGF-23) was high 399RU/ml (<100). Patient noticed left thigh lump which was 3.5cmx3.2cmx2cm in subcutaneous plane. Excision-biopsy confirmed benign lesion. Diagnosis of TIO confirmed. Patient had dramatic improvement in muscle strength as phosphate levels normalised within days. 1,25(OH)2 Vit D and ALP normalised in few weeks. Oral phosphate and vit D treatment stopped. He had normal phosphate and FGF-23 on follow-up signifying no recurrence.

Average time between symptom-onset and diagnosis of TIO is 2.5 years and further 2.5 years for tumour-localisation. The main differential is X-linked hypophosphatemia. Main findings are low serum phosphate, low/normal 1,25-(OH)2-Vit D, reduced TMP/GFR, elevated FGF-23, normal serum calcium and 25-(OH)-Vit D and elevated ALP. Non-localised TIO can be treated with phosphate, vitamin D and Burosumab.

Following the case, we carried our audit on hypophosphatemia management in our hospital. There are no national guidelines for hypophosphatemia. Our trust guidelines recommend treating symptomatic mild-moderate hypophosphatemia (0.3-0.59mmol/L) with oral Phosphate-Sandoz (1-2TDS) and Severe hypophosphatemia (<0.3mmol/L) with Phosphate-Polyfuser infusion. We collected six-months data of inpatient phosphate tests.

### Results:

We identified 361 patients with phosphate <0.5mmol/L (Moderate hypophosphatemia). 31 patients had Severe Hypophosphatemia (<0.3mmol/L), 20 discharged and 11 patients passed away. 12 patients (38%) did not receive any phosphate correction and rest were not compliant with local protocol.

**Conclusion:**

We presented the findings in Grand rounds and educated clinicians. Hypophosphatemia is commonly overlooked entity and is mostly secondary to underlying causes. National guidelines for hypophosphatemia management would help standardise care.

**Table 1: Phosphate replacement and outcome in patients with Severe hypophosphatemia**

| Diagnosis | Phosphate Replacement given | Outcome |
|---|---|---|
| DKA | No | Home |
| DKA | No | Home |
| DKA | No | Home |
| SBO | No | Home |
| Sepsis COVID | No | Home |
| LBO Post op | No | Home |
| Paraparesis/Vertebral lesion | No | Home |
| Cholangitis | No | Home |
| DKA | Yes | Home |
| DKA | Yes | Home |
| DKA | Yes | Home |
| DKA | Yes | Home |
| Myeloma/Sepsis | Yes | Home |
| DKA | Yes | Home |
| GI Ulcer/Vomiting | Yes | Home |
| Burns/Intubation | Yes | Home |
| ALD | Yes | Home |
| Chemo/Diarrhoea | Yes | home |
| UGI Bleed | Yes | Home |
| Indapamide | Yes (Oral) | Home |
| | | **20** |

| Diagnosis | Phosphate Replacement given | Outcome |
|---|---|---|
| Sepsis | No | RIP |
| Lymphoma/Sepsis | No | RIP |
| DKA | No | RIP |
| Met Bladder CA/Unwell | No | RIP |
| DKA | Yes | RIP |
| HCC/Hepatic failure | Yes | RIP |
| Met CA/C. Diff diarrhoea | Yes | RIP |
| Met Bladder CA/Liver met | Yes | RIP |
| Sepsis Multi-organ | Yes | RIP |
| Cirrhosis | Yes (Oral) | RIP |
| Lung CA/Metabolic acidosis | Yes (Oral) | RIP |
| | | **11** |

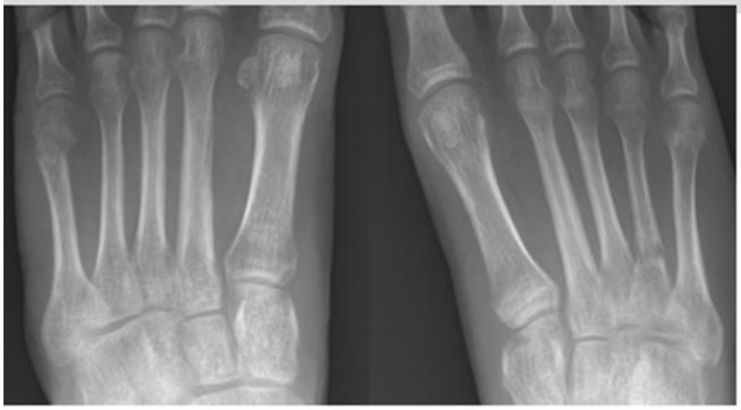

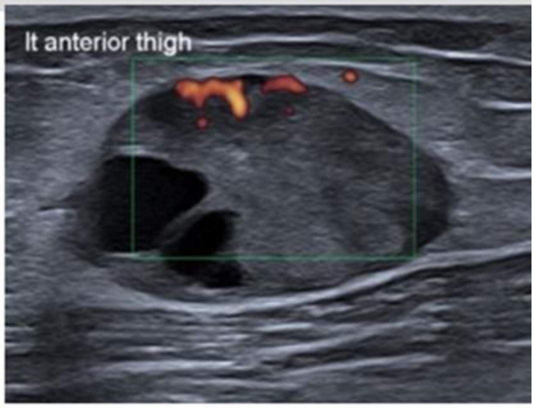

**P33**

Withdrawn

### P34

### Weekly vitamin D and daily calcium carbonate increase bone density over 48-weeks in adolescents with HIV and 25(OH)D <75nmol/L: a placebo-controlled trial in Southern Africa

Prof Celia Gregson[1,2], Mr Tafadzwa Madanhire[1,2], Dr Nyasha Dzavakwa[2,3], Prof Lackson Kasonka[4], Ms Hildah Banda-Mabuda[4], Ms Tsitsi Bandason[2], Ms Molly Chisenga[4], Prof Suzanne Filteau[3], Prof Katharina Kranzer[3,2], Prof Hilda Mujuru[5], Prof Ulrich Schaible[6], Prof Sarah Rowland-Jones[7], Dr Victoria Simms[3], Prof Rashida Ferrand[3]

[1]University of Bristol, Bristol, UK. [2]Biomedical Research and Training Institute, Harare, Zimbabwe. [3]London School of Hygiene and Tropical Medicine, London, UK. [4]University Teaching Hospital, Lusaka, Zambia. [5]University of Zimbabwe, Harare, Zimbabwe. [6]Research Centre Borstel, Leibniz Lung Centre, Borstel, Germany. [7]University of Oxford, Oxford, UK

### Abstract

**Background**: Despite antiretroviral therapy (ART), perinatally acquired Human Immunodeficiency Virus (HIV) infection adversely affects adolescent skeletal development. Furthermore, vitamin D insufficiency is common among people living with HIV.

**Aim:** To determine whether vitamin D and calcium supplementation improves bone density in adolescents living with HIV.

**Methods**: A multi-country individually randomised, double-blinded placebo-controlled trial of weekly vitamin D (20,000IU) plus daily calcium carbonate (500mg) for 48 weeks was conducted. Adolescents with HIV age 11-19 years, taking ART for ≥6 months were recruited from HIV clinics in Harare, Zimbabwe and Lusaka, Zambia. The primary outcome was DXA-measured total body less-head bone mineral density (TBLH-BMD) Z-score, derived using UK reference population data, the secondary outcome was lumbar spine bone mineral apparent density (LS-BMAD) Z-score. Linear regression compared arms adjusting for study site and the baseline bone density value. Sub-group analyses by baseline vitamin D insufficiency (defined in this region as 25(OH)D <75nmol/l) were pre-specified.

**Results**: In total 842 adolescents [53.2% female] were enrolled; most (75.9%) had a 25(OH)D concentration <75nmol/l. HIV had been diagnosed at a median age of 5 [IQR 2-9] years. At 48-week follow-up 751 (89.2%) had a DXA performed. Overall, there was no difference by arm in mean 48-week TBLH-BMD Z-score (-1.56 [SD 1.12] in the intervention vs -1.53 [1.18] the control arm; adjusted mean difference -0.03 (95%CI -0.08, 0.02)). Findings were similar for LS-BMAD Z-score. However, in those with baseline 25(OH)D <75nmol/l both TBLH-BMD and LS-BMAD Z-scores were higher in those randomized to supplementation (Table 1). No evidence of an interaction by age, sex or puberty was detected. No drug-related severe adverse events occurred.

**Conclusions:** High-dose vitamin D and low-dose calcium are safe and cheap interventions to give during adolescence. In adolescents growing up with HIV in Africa, this supplementation may promote bone accrual towards maximizing peak bone mass, which may reduce future fracture risk.

| Baseline 25(OH)D concentration | N | Supplemented Mean (SD) | Placebo Mean (SD) | Adjusted Mean Difference (95%CI) | P-value | Interaction P-value |
|---|---|---|---|---|---|---|
| **TBLH-BMD Z-score** | | | | | | |
| <75nmol/L | 562 | -1.53 (1.22) | -1.61 (1.13) | 0.04 (0.00, 0.08) | 0.027 | 0.078 |
| ≥75nmol/l | 189 | -1.52 (1.03) | -1.45 (1.12) | -0.05 (-0.16, 0.07) | 0.44 | |
| **LS-BMAD Z-score** | | | | | | |
| <75nmol/L | 558 | -0.64 (1.19) | -0.71 (1.13) | 0.04 (0.02, 0.11) | 0.016 | 0.013 |
| ≥75nmol/l | 188 | -0.51 (1.08) | -0.70 (1.24) | -0.10 (-0.23, 0.03) | 0.13 | |

### P35

### 3D imaging of murine subchondral bone in situ reveals an altered osteocyte phenotype in osteoarthritis

Dr Lucinda Evans[1,2], Dr Aikta Sharma[3], Ms Alissa Parmenter[3], Dr Sebastian Marussi[3], Dr Catherine Disney[4], Dr Kamel Madi[5], Professor Peter Lee[3], Professor Andrew Pitsillides[1], Professor Katherine Staines[2]

[1]Royal Veterinary College, London, UK. [2]University of Brighton, Brighton, UK. [3]University College London, London, UK. [4]Diamond Light Source, Oxfordshire, UK. [5]3Dmagination, Oxford, UK

### Abstract

### Objectives:

Condylar microarchitecture is a likely risk factor for osteoarthritis vulnerability. We aimed to anatomically characterise and compare the tibial subchondral bone (SCB) of male osteoarthritic (OA, STR/Ort) and healthy-ageing (CBA) mouse models, in high-resolution, at both pre-OA (10 weeks) and OA (40+ weeks) timepoints.

### Methods:

Whole intact mouse knees (n=4 per strain/age group) were synchrotron-CT scanned at the European Synchrotron Radiation Facility (1.45µm/voxel). Samples were secured in situ by the application of a 2N load using a mechanical loading rig. Following tomographic reconstruction, proximal tibial epiphyseal SCB and its resident osteocyte lacunae and vasculature were semi-automatically segmented out from the subchondral plate in 3D using Avizo (Fig. A-B) and analysed in CTAn.

### Results:

Linear mixed model analysis confirmed that osteocyte lacunar number was greater on the medial (most severely OA-affected) tibial condyle in STR/Ort mice than controls ($p<0.01$, Fig. C) even at pre-OA ages. These OA-associated osteocyte lacunae were, additionally, smaller than healthy CBA lacunae in both volume and diameter ($p<0.001$, Fig. C). STR/Ort mice also had greater subchondral invasion by vasculature of greater diameter on the OA-prone medial condyle.

### Conclusions:

Our findings show that bony sclerosis and vascular invasion of the medial tibia, well-documented in both human and STR/Ort OA, is here accompanied by an altered resident osteocyte phenotype. Pre-OA and OA medial tibial condyles both contained an increased number of significantly smaller osteocyte lacunae than healthy controls. These data suggest a causal mechanism underlying the onset of bone symptoms in OA, as osteocytes encased within smaller lacunae could be predicted to experience mechanical loads travelling through the knee joint differently, due to stress-shielding. High-resolution scanning and semi-automated segmentation have enabled the SCB contents to be analysed accurately and independently without inclusion of overlying calcified cartilage or underlying trabecular bone (Fig. A). Our findings highlight the value of synchrotron-CT imaging to facilitate major changes in our current understanding of osteoarthritis pathobiology.

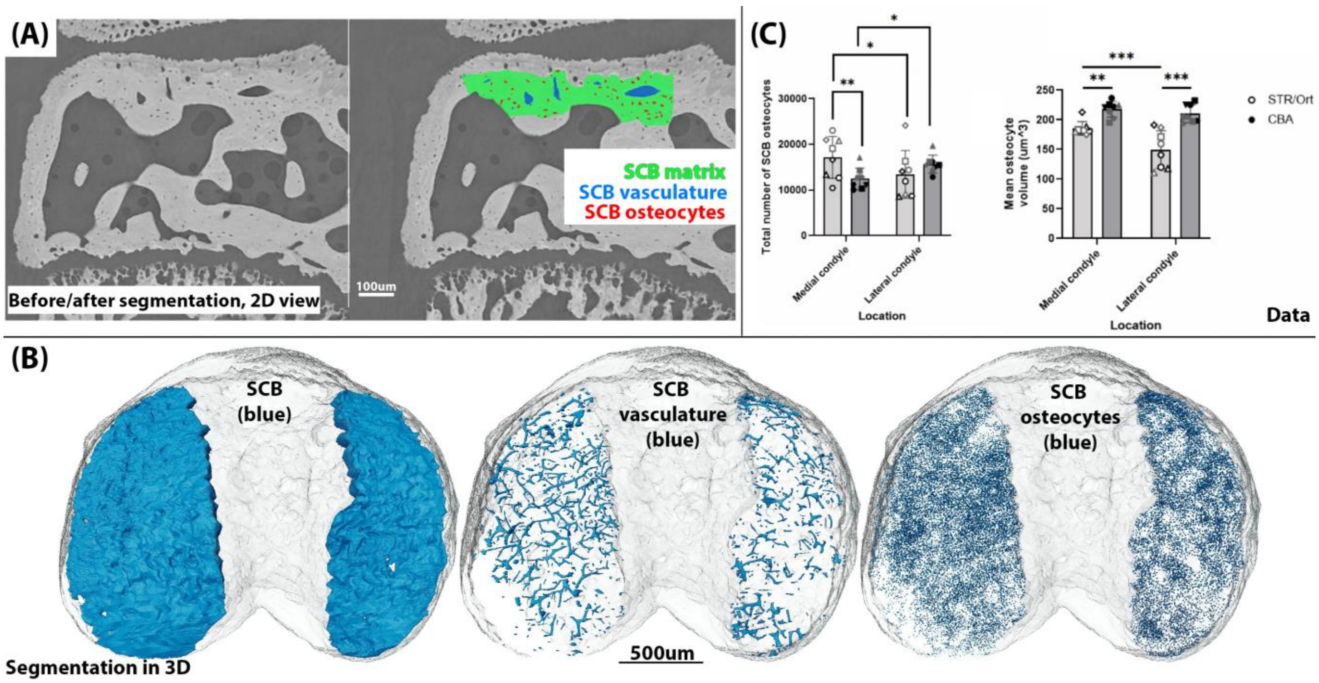

(A)
SCB matrix
SCB vasculature
SCB osteocytes
100um
Before/after segmentation, 2D view
(C)
Total number of SCB osteocytes
30000
20000
10000
0
Medial condyle
Lateral condyle
Location
Mean osteocyte volume (um^3)
250
200
150
100
50
0
STR/Ort
CBA
Data
(B)
SCB (blue)
SCB vasculature (blue)
SCB osteocytes (blue)
500um
Segmentation in 3D

### P36

### Investigating the molecular mechanisms underpinning post-traumatic osteoarthritis in non-invasive mechanically loaded mouse models

Miss Kamakshi Jani, Miss Aisha Mohamed, Mr Shadi Amor, Dr Hasmik Jasmine Samvelyan

Anglia Ruskin University, Chelmsford, UK

### Abstract

### Introduction
Osteoarthritis, a leading cause of disability, has a significant socioeconomic burden and major healthcare costs worldwide. However, currently there are no treatments for osteoarthritis, patients are offered symptomatic cures for symptom alleviation, pain reduction and improvement of joint function.

The aim of this study was to investigate mechanoadaptive and biomechanical properties of the epiphyseal growth plate underpinning osteoarthritis pathology in non-invasive mechanically loaded murine models of post-traumatic osteoarthritis.

### Methods
Right knee joints of 17-week-old C57BL/6 male mice ($n$ = 7) were subjected to non-invasive axial loading with 8N or 11N forces 3 times per week on alternating days for 2 weeks under isoflurane-induced anaesthesia. Left knee joints were non-loaded controls. High-resolution micro-CT scans (5µm slices at 50kV, 200µA, 0.5mm aluminium filter, 0.6° rotation angle) were acquired, reconstructed, and realigned using DataViewer. Scans were then segmented using a region growing python algorithm within Avizo software. Analysis of lateral and medial tibiae bridge number and areal densities were performed using Avizo software. The study received Research Ethics Committee approval and 3Rs principle followed as the ethical framework for conducting all the experiments.

### Results
Growth plate bridge number and areal densities were increased in medial compared to lateral tibiae of all the mice loaded with 8N or 11N force compared with non-loaded left tibiae respectively (bridge number; loaded medial versus lateral 8N 102 ± 25 vs 64 ± 17 ($p$ = 0.004), 11N 101 ± 28.8 vs 73 ± 24.5 ($p$ = 0.03); bridge areal density; loaded medial versus lateral 8N 2.90 ± 0.46 vs 2.23 ± 0.57($p$ = 0.03), 11N 3.17 ± 0.55 vs 2.97 ± 0.57 ($p$ = 0.34)), but there were no differences between those of loaded knee joints.

### Conclusions
Formation of epiphyseal growth plate bony bridges has been accelerated in both cyclic articular cartilage tibial compression models. The non-invasive dynamic mechanical loading with either 8N or 11N force regimens applied to mouse tibiae through knee and ankle joints has modified joint structure locally through a homeostatic mechanoadaptive response advancing our understanding of growth plate dynamics, joint tissue molecular mechanisms and how these may contribute to the osteoarthritis development. Future studies will further determine these associations, establish biomechanical and morphological properties across articular and calcified cartilage, subchondral bone properties underpinning post-traumatic osteoarthritis development and examine relevance to human osteoarthritis.

### P37

### Obese adipose-derived extracellular vesicles drive skeletal muscle atrophy: Implications for obesity and age-related muscle loss

Mr Michael Macleod[1], Dr Joshua Price[1], Miss Caitlin Ditchfield[1], Prof Kostas Tsintzas[2], Prof Simon Jones[1]

[1]University of Birmingham, Birmingham, UK. [2]University of Nottingham, Nottingham, UK

### Abstract

Sarcopenic obesity, characterised by excess adiposity and diminished skeletal muscle mass and function, is associated with frailty and inflammation. Previous work from our group implicated dysregulated crosstalk between adipose tissue (AT) and skeletal muscle (SkM) in driving sarcopenic obesity, and increasing evidence demonstrates that extracellular vesicles (EVs) facilitate intercellular communication. We therefore aimed to characterise AT-EV profiles across lean and obese subjects, map these to parameters of inflammation and adiposity, explore their effects on SkM, and identify mechanisms of action.

Adipose conditioned media (ACM) was generated from ex vivo tissue explants, and EVs were extracted by ultracentrifugation. EV profiles were analysed using ExoView and nanoparticle tracking analysis. Human primary SkM cells were treated with ACM and AT-EVs for 24h (post-differentiation) or 8-days (during differentiation), with myotube thickness and nuclear fusion as functional readouts. RNA was extracted from treated myotubes to assess gene expression.

ExoView indicated classical tetraspanin EV marker expression within the population. Obese AT released fewer EVs than lean AT, with consistently higher EV yield from visceral AT compared to subcutaneous AT, supported by EV-associated protein concentration trends. Obese AT-EVs significantly reduced myotube thickness and increased expression of atrophic and inflammatory genes compared to untreated controls, suggesting modulation of catabolic and anabolic pathways mediating SkM hypertrophy. Interrogating these molecular pathways in the contexts of adiposity, exercise-like stimulation, and age-related muscle loss will advance understanding of mechanistic drivers of sarcopenic obesity.

Obese AT-EVs drive atrophic signatures in SkM, inviting further research into EV-mediated crosstalk in sarcopenic obesity.

### P38

### Identification of the genetic determinants and clinical implications of bone marrow adiposity in the UK Biobank: a meta-GWAS and MR-PheWAS study

Dr Wei Xu[1], Dr Mesa-Eguiagaray Ines[1], Dr David M Morris[1], Dr Chengjia Wang[2], Dr Calum Grey[1], Mr Samuel Sjostrom[1], Dr Giorgos Papanastasiou[1], Dr Sammy Badr[3], Dr Julien Paccou[3], Dr Xue Li[4], Dr Paul R. H. J. Timmers[1], Dr Maria Timofeeva[5], Dr Tom MacGillivray[1], Dr Scott IK Semple[1], Professor Evropi Theodoratou[1], Dr William P Cawthorn[1]

[1]Univerisity of Edinburgh, Edinburgh, UK. [2]Heriot-Watt University, Edinburgh, UK. [3]Marrow Adiposity and Bone Laboratory (MABlab), Lille, France. [4]Zhejiang University, Hangzhou, China. [5]University of Southern Denmark, Odense, Denmark

### Abstract

Bone marrow adipose tissue (BMAT) comprises >10% of total adipose mass in healthy humans. It further increases in ageing and diverse diseases, including osteoporosis and fracture. However, BMAT's pathophysiological functions and genetic determinants remain unknown. In humans, bone marrow adiposity is typically measured as the bone marrow fat fraction (BMFF) using magnetic resonance imaging (MRI).

Herein, we used deep learning to measure the BMFF of the femoral head, total hip, femoral diaphysis, and spine from MRI of >48,000 participants (>42,000 white, >6,500 non-white) in the UK Biobank imaging study. We conducted the largest genome-wide association meta-analyses (meta-GWAS) to date, identifying 67, 147, 134, and 174 independent significant single nucleotide polymorphisms (SNPs), which were mapped to 54, 90, 43, and 100 genes associated with BMFF at each respective site in the white population. To understand putative biological mechanisms and functional roles of BMFF-associated variants, we first performed MAGMA gene-set analysis, tissue expression analysis, and cell-type-specific gene expression analysis, highlighting pathways related to adipogenesis and bone remodeling. We then conducted transcriptome-wide association studies (TWAS) [genes: head=31; total hip=49; diaphysis=32; spine=64] and colocalization analyses, which identified risk genes whose genetically regulated expression levels in adipose tissues, skeletal muscle, and/or lymphoid tissues were associated with altered BMFF. In addition, we conducted multi-ancestry meta-GWAS, which identified 121, 314, 234, and 310 independent significant SNPs which were mapped to 65, 98, 63, and 121 genes for the head, total hip, diaphysis, and spine, across different ethnic groups.

Following the meta-GWAS, we then generated BMFF polygenic risk scores (PRSs) for each bone region, and investigated disease outcomes associated with deep learning measured and genetically proxied BMFF levels using phenome-wide association (PheWAS) analyses (1808 PheCODEs classified into 17 disease categories). These confirmed positive associations between BMFF and osteoporosis at all four skeletal sites while also revealing unexpected site-specific associations with other diseases, including multi-morbidities that impose substantial health burdens. We further conducted two-sample Mendelian randomization (MR) analyses to investigate the potential causal associations between BMFF and these disease outcomes. The findings suggest that genetic predisposition to increased BMFF is causally associated with metabolic and musculoskeletal disorders.

As the first large-scale meta-GWAS and MR-PheWAS for bone marrow adiposity, these findings provide unprecedented insight into BMAT formation and function and highlight new possibilities for BMAT as a biomarker and therapeutic target for the improved prevention and treatment of human diseases.

### P39

### Forecasting future hip fracture incidence in NHS Highland to aid service planning and prevention

Ms Sarah Griffin[1], Mr John MacKintosh[1], Ms Carolyn Hunter-Rowe[1], Dr Stephen Bridgman[1,2,3,4]

[1]NHS Highland, Inverness, UK. [2]Public Health Scotland, Edinburgh, UK. [3]Argyll & Bute Health Social Care Partnership, Helensburgh, UK. [4]University of Aberdeen, Aberdeen, UK

### Abstract

Background. Hip fractures in older populations are one of the most frequent reasons for emergency admission and a major public health issue. In this study we aimed to forecast likely increases in incidence to aid service planning, policy and prioritisation.

Methods. Hospital activity for NHS Highland (NHSH) residents aged 50yrs and over was sourced from Scottish Morbidity Records provided by Public Health Scotland. Non-elective episodes of hip fracture were selected based on ICD-10 codes S720-722 in any diagnostic position and were broken down by age, sex, financial year, urban-rural classification, and deprivation. Future population projections were from National Records of Scotland. All analyses were conducted in R. Models compared were time series modelling; a fixed rate model; and Poisson regression modelling.

Results. There were 8,964 patients in the dataset. The incidence of hip fractures for NHSH increased 58% from 2000/01 to 2022/23. The modal age was 85-89yrs, with 77% of fractures occurring in those aged over 75yrs. The age-standardised rate of fractures was two-fold higher in women than men. Age-sex standardised rates of hip fractures decreased since 2001/02. There was no significant difference based on urban-rural classification. Variation in rates by deprivation was relatively small compared to the effect of age and sex, with higher rates in more deprived areas. The average length of stay was consistent at 11days for the last decade, but there was a small increase with age from a median of 7days up to 65yrs and 9days for those 85yrs and over. Changes in demography generated a forecasted 19% increase in hip fractures for NHSH between 2022 and 2030 using both regression and statistical process control models. A 30% increase in the over 80yrs age-band was forecast and a higher percentage increase was forecast in males compared to females. The time-series model did not account explicitly for demographic changes; it produced higher forecasted numbers of hip fractures.

Discussion. While it is positive that age-sex standardised rates have been falling over the last twenty years, a 19% increase is a concern both due to increased disability and resource utilisation in NHS Highland. The time-series model may overestimate future increases. This work is being used to advocate for a national target/indicator for age-sex standardised rate of hip fractures to help move the management and prevention of hip fractures, and other osteoporotic fractures, further up the policy agenda at both local health board and national level.

### P40

### Vertebral fracture severity and glucocorticoid dosing regimes predict vertebral fracture progression in boys with Duchenne muscular dystrophy (DMD)

Dr Nicola Crabtree[1,2], Dr Sarah McCarrison[3,4], Dr Sze Choong Wong[3,4], Dr Suma Uday[1,2], Dr Vrinda Saraff[1,2]

[1]BWC NHS Trust, Birmingham, UK. [2]University of Birmingham, Birmingham, UK. [3]Royal Hospital for Children, Glasgow, UK. [4]University of Glasgow, Glasgow, UK

### Abstract

**Objectives:** Vertebral fractures (VF) are a recognised sequelae in boys with DMD receiving glucocorticoids (GC). Once identified, current treatment and prevention of further VFs is by bisphosphonate therapy. The objective of this review was to observe the changes in VF and back pain twelve months pre and post bisphosphonate initiation, in two different GC regimes.

**Methods:** A retrospective longitudinal review of boys with DMD and VF, initiated on intravenous bisphosphonates, between April 2014 and January 2023, due to concerns regarding VF. Inclusion criteria was spine imaging immediately prior to bisphosphonates and at least one lateral spine image in the preceding and following 12 months. Annual spine monitoring was performed with lateral dual-energy X-ray absorptiometry. Number and grade of VF was documented using the spine deformity index(SDI). Clinical details including GC regime, mobility, pubertal status, back pain, and bone density were collected. Boys were categorised according to GC treatment regime; daily (D) or intermittent (INTER). Boys on the daily regime were further sub-divided according to VF severity at treatment initiation using the following threshold: SDI< 6 = daily mild (D-M) or SDI >= 6 = daily severe (D-S).

**Results:** 34 boys were included; 13 were on intermittent (10 days on/10 days off) and 21 on daily CG regime of whom 7 had SDI≥6 at treatment initiation. At the time of treatment, INTER boys were older [INTER/D-M/D-S: 12.8(1.7)/10.7(2.2)/11.6(1.5) years, p=0.03] with longer time on GC therapy [INTER/D-M/D-S: 7.0(2.4)/4.2(2.5)/4.6(2.1) years, p=0.012]. INTER boys were also more likely to be pubertal but be less ambulant than both daily groups. Progression was rapid for boys in the D-S group compared to both INTER or D-M boys(see figure). Frequency of back pain was highest at treatment initiation but appeared to stabilise post initial bisphosphonate infusion [Back pain at treatment initiation: 54%, 64%, 83% versus 12m post: 18%, 55%, 29%, for INTER, D-M and D-S, respectively].

**Conclusion:** VF progression is most severe in boys on daily GC therapy with more severe VFs prior to treatment. Bisphosphonate treatment stems the development of VFs and reduces back pain. Early intervention, particularly for boys on daily GC with only mild VF appears to mitigate the most severe and painful VFs.

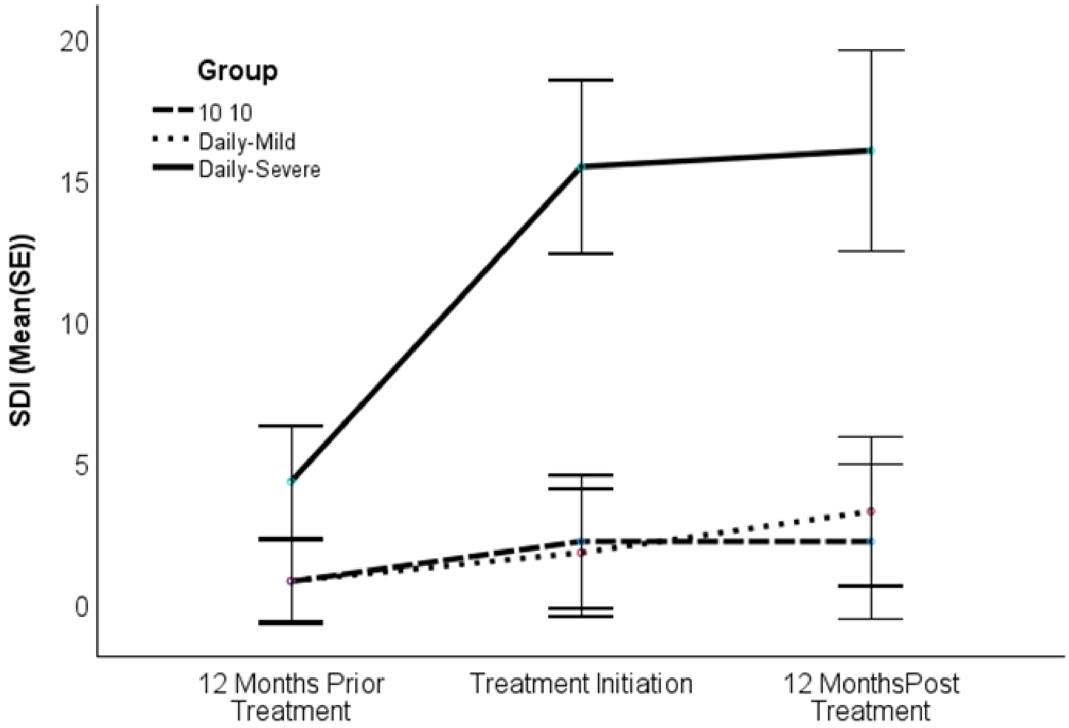

20
15
10
5
0
SDI (Mean(SE))
Group
10 10
Daily-Mild
Daily-Severe
12 Months Prior Treatment
Treatment Initiation
12 MonthsPost Treatment

**P41**

Withdrawn

### P42

### On bone supporting yellow marrow and yellow marrow loading bone

Professor Alan Boyde

Queen Mary University of London, London, UK

### Abstract

Central marrow cavities towards distal ends of long bones in large mammals, including man and horse, contain a sometimes seemingly random trabecular arrangement which is not evidently related to any significant load bearing function or direction: the rods in this network are so slender that they cannot be detected by clinical imaging methods. We have supposed that their function is to support the intervening marrow, which is a kind of 3D bubble wrap of ~50µm diameter adipocytes filled with low viscosity oil of density ~ 0.9. But is - and how is - this fatty marrow attached to bone?

Material, all normal, healthy, archival: (a) sectioned, macerated Thoroughbred racehorse distal third metapodials, 3D trabecular architecture documented by photography, flat-bed scanner imaging, and scanning electron microscopy: (b) two year old Thoroughbred racehorse third metacarpals, longitudinal slabs, aldehyde fixed, defatted and dehydrated in ethanol, embedded in polymethylmethacrylate, block top surfaces micromilled, transparent 2mm sections studied, unstained, by polarised transmitted light microscopy, using both (i) circularly polarised light (CPL) with through focus series, and (ii) multiple rotations of crossed linear polarising filters (MRXPL), images recorded at 7.5° intervals, 'stacks' or pairs processed in different ways using ImageJ – this latter methodology is exquisitely sensitive to detecting birefringent materials.

Results: a complex 3D network of fine fibres permeates normal fatty marrow and is sparsely attached to bone over flattish surfaces but heavily focussed onto nodes in the trabecular continuum, such as real ends of rods (Figure) and triple junctions. Marrow is thereby shown to be firmly tethered to trabecular bone.

Two modes of marrow behaviour during loading of deeper subchondral cancellous bone are considered in the literature: both have treated the kinds of cancellous subchondral bone organised as plates, a honeycomb, or with more equidiametrical cavities containing and restraining fatty marrow. The theories are *either* liquid (fat + water) may move between compartments as bone is compressed *or* there is no free movement because the viscosity of the marrow is too high. That fatty marrow may be directly attached to bone has never been considered. The present studies have shown for the first time that such is the case, so that new concepts of marrow function during bone loading need to be concocted.

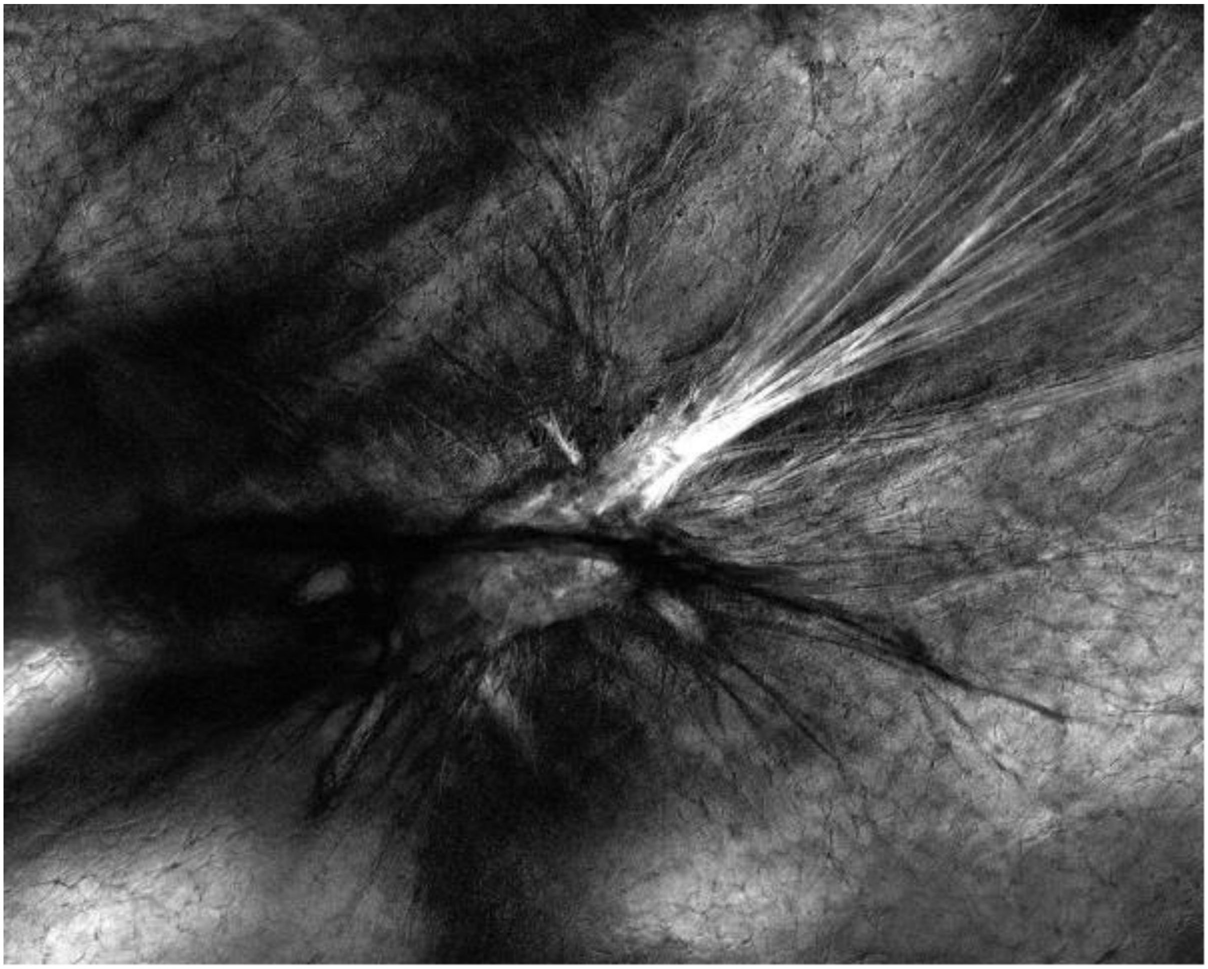

MRXPL horse distal Mc3 marrow, end of rod entering field from left, width 2150µm

### P43

### Systems-level analysis of total body [$^{18}$F]FDG PET/CT to study bone metabolism in adults

Ms María Paula Huertas Caycedo[1], Dr Calum Gray[2], Ms Kayla Bell[3], Ms Keira Young[3], Dr William Cawthorn[1], Prof. Colin Farquharson[4], Dr Benjamin Spencer[5], Dr Yasser Abdelhafez[5], Prof. Adriana Tavares[1,2], Prof. Simon Cherry[6], Prof. Roland Stimson[1], Dr Karla Suchacki[1,3]

[1]University/BHF Centre for Cardiovascular Science, University of Edinburgh, Edinburgh, UK. [2]Edinburgh Imaging Facility, Queen's Medical Research Institute, Edinburgh, UK. [3]Scotland's Rural College, Edinburgh, UK. [4]The Roslin Institute, University of Edinburgh, Edinburgh, UK. [5]EXPLORER Molecular Imaging Center, University of California, Sacramento, USA. [6]Departments of Biomedical Engineering and Radiology, University of California, California, USA

### Abstract

**Background:** Our understanding of complex tissue interactions at a systems level remains rudimentary, limiting our ability to dissect mechanisms underlying diseases and develop novel therapeutics. Skeletal research has focused on the pathogenicity of obesity and diabetes mellitus on the skeleton; however, the skeleton is not merely an endocrine target but also a secretory organ, modulating systemic energy homeostasis. Fluorodeoxyglucose [$^{18}$F]FDG positron emission tomography and computerised tomography (PET/CT) is an integral component for staging of cancer patients and for assessment of treatment efficacy. [$^{18}$F]FDG PET/CT is also currently the method of choice for the evaluation of brown adipose tissue (BAT). In contrast to white adipose tissue, which is specialised for energy storage, BAT enhances energy expenditure in response to cold activation and is responsible for non-shivering thermogenesis. The presence of BAT has been shown to have beneficial effects on metabolic regulation, cardiovascular health and on bone mass by facilitating osteogenesis and suppressing osteoclastogenesis[1]. We have recently discovered that different bones within the murine skeleton have a unique glucose metabolism and form a complex metabolic network[2].

**Hypothesis:** Different bones within the human skeleton have unique molecular signatures and form a distinct metabolic network, which is altered by the presence of BAT.

**Methods:** Age-matched, male and female BAT-negative (n=15, 64.1 ± 14.4 years, 26.4 ± 5.1kg/m$^2$) and BAT positive patients (n=15, 64.5 ±14.0 years, 25.0 ± 5.4 kg/m$^2$) were identified from static total body [$^{18}$F]FDG PET/CT. Multi-organ objective segmentation (MOOSE) was used for organ delineation. Bone marrow adipose tissue (BMAT), red marrow (RM) or bone were segmented using diagnostic Hounsfield Unit (HU) thresholds (Figure 1), and BAT using the BARCIST criteria. Standardised uptake values (SUV) were used to perform network analysis (Graphia).

**Results:** There was an increased incorporation of [$^{18}$F]FDG in the axial skeleton compared to the appendicular skeleton in both BAT positive and negative groups. BAT had no effect on incorporation of [$^{18}$F]FDG into the bone and BMAT, however network analysis indicated a shift in bone metabolic profiles between both groups.

**Conclusion:** Different bones within the human skeleton have a unique glucose metabolism and form complex metabolic networks, which are altered by the presence of BAT.

1. Du J, He Z, Xu M, et al. Brown Adipose Tissue Rescues Bone Loss Induced by Cold Exposure. *Front Endocrinol (Lausanne)*. 2022;12. doi:10.3389/fendo.2021.778019

2. Suchacki KJ, Alcaide-Corral CJ, Nimale S, et al. A Systems-Level Analysis of Total-Body PET Data Reveals Complex Skeletal Metabolism Networks in vivo. *Front Med (Lausanne)*. 2021;8. doi:10.3389/

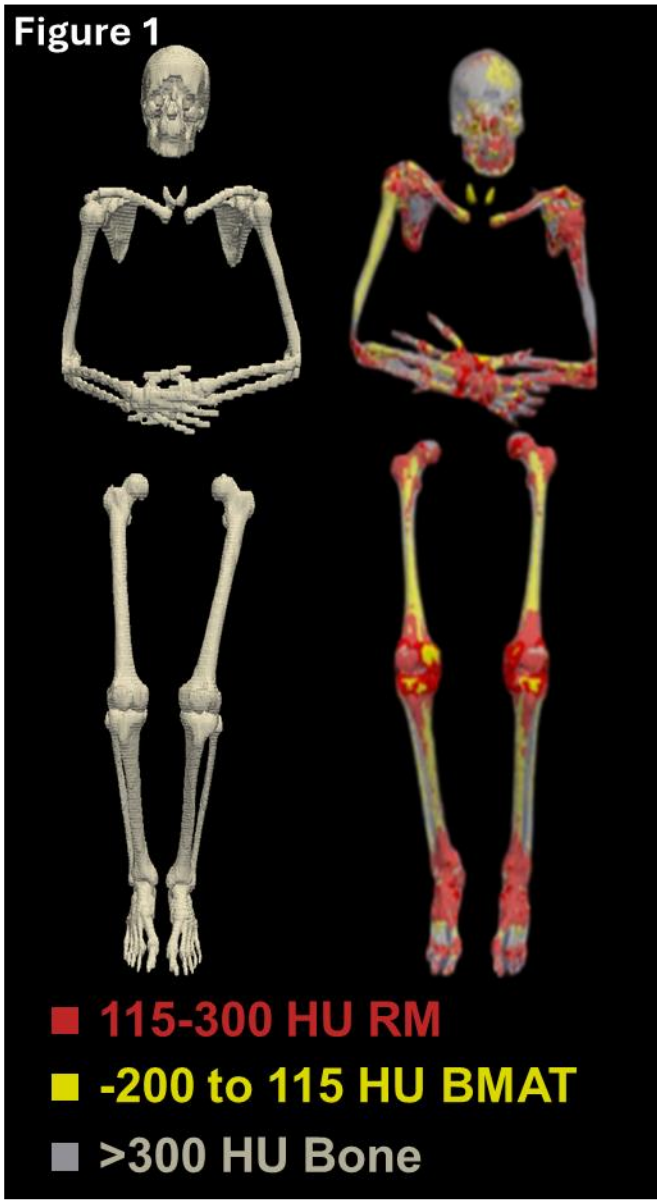

Figure 1

**P44**

### Patient quantification of second lumbar vertebral body mineral concentration with X-ray micro-tomography

Dr David Mills, Prof Alan Boyde

Queen Mary University of London, London, UK

### Abstract

The application of X-ray micro-tomography (XMT, uCT) to the study of bones of all sorts is widely accepted but perhaps with too little comment. The voxel resolution is inevitably limited by the size of the physical sample under study, and analyses will always be affected by partial volume effects where bone voxel values are blurred with either low density non-bone elements such as osteoid, marrow, periosteum, cartilage, nucleus pulposus, annulus fibrosis, tendon and ligament, or with higher density calcified non-bone tissues including periosteum, cartilage, fibrocartilage, tendon and ligament.

We studied ~4mm thick parasagittal bone slabs from 69 second lumbar vertebral body obtained 30 years ago from donors aged 23 to 92 years via the European Union BIOMED I project “Assessment of Bone Quality in Osteoporosis”; embedded in polymethylmethacrylate (PMMA) after dehydration and defatting in ethanol, one block surface finished by micro-milling. Initial studies used quantitative backscattered electron scanning electron microscopy (qBSE-SEM) to characterise mineral concentration at sub-micron voxel resolution.

XMT used the QMUL MuCAT2 system: 90 kV, 180 µA, beam hardening filters 1.2 mm Al and 0.05 mm Cu, Time Delay Integration readout CCD detector, scan time ~48 hours, total x-ray dose >30 Gy, reconstructed voxel size 30 microns. Calibration was performed by scanning a 9-metal carousel immediately after the sample, fitting a polynomial to the carousel data and using this to estimate the x-ray spectrum and detector response. One voxel was digitally stripped from all surfaces before analysis to circumvent partial volume effects: this returns sharper histograms by removing dubious edge voxels, giving more reliable – and higher – mineral concentrations.

In XMT, the higher density calcified non-bone tissue phases demonstrated in the prior qBSE-SEM imaging of the same samples typically have densities beyond the normal range for bone tissue proper. This is particularly important in distinguishing calcified cartilage in end-plates and ‘Schmorl’s nodes’ and calcified ligament and periosteum in cortices.

Determining the tissue types and degree of mineralisation in vertebral bodies comprised of mixed tissue types with different degrees of mineralisation with micro-tomography is more than challenging due to inaccuracies in the reconstructed linear attenuation coefficient caused by using standard laboratory polychromatic X-ray sources and short integration times. High-density phases are obviously blurred into 'bone' in clinical CT imaging but have also not been returned as separate materials in prior published ‘micro’CT studies.

Uncalibrated (50%)
994x1097 pixels; RGB; 4.2MB
Calibrated (50%)
994x1097 pixels; RGB; 4.2MB

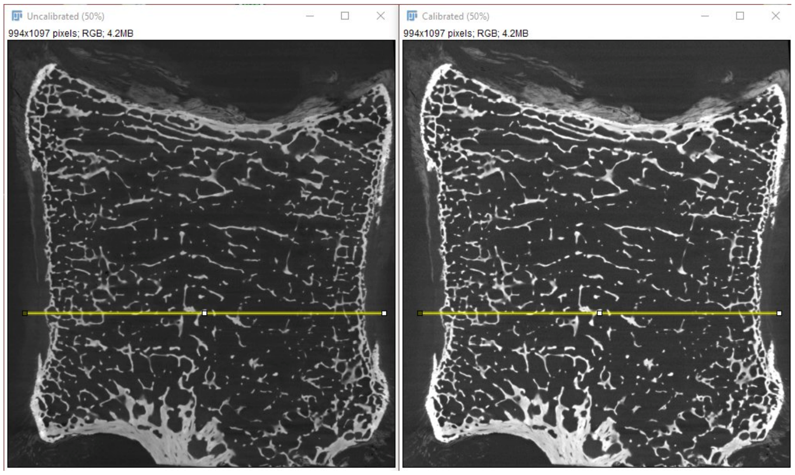

Calibrated Vs Uncalibrated LAC
LAC
300
250
200
150
100
50
0
0
100
200
300
400
500
600
700
800
900
Distance in Pixels
Calibrated
Uncalibrated

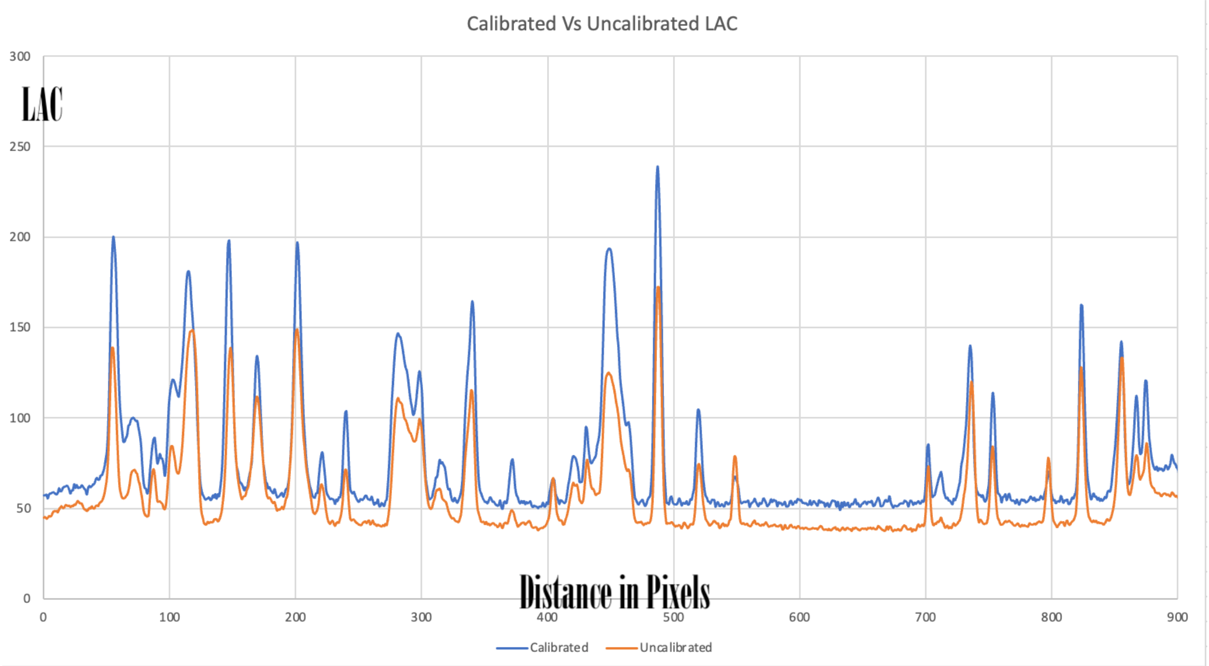

Difference
LAC
100
80
60
40
20
0
-20
0
100
200
300
400
500
600
700
800
900

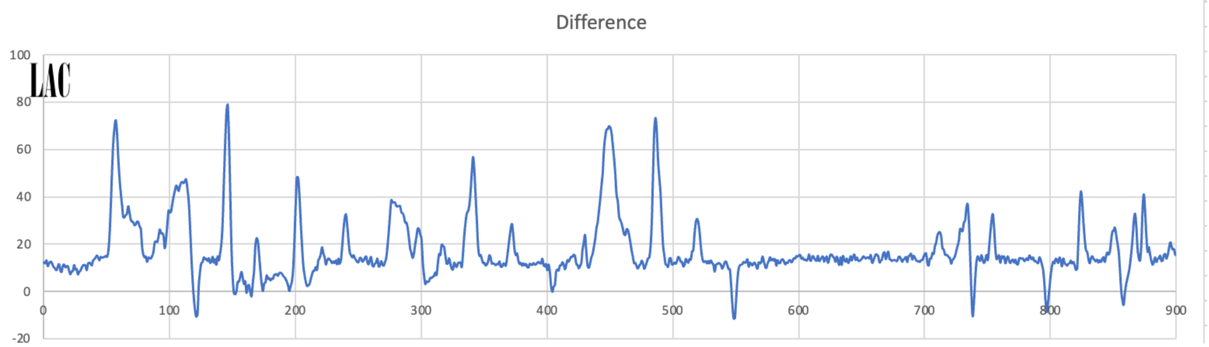

### P45

### Evaluating the Relationship Between Type 2 Diabetes, Popliteal Artery Circumference and Osteoarthritis in the Human Knee

Mr Paul Rothwell, Dr Alistair Bond, Professor James Gallagher

University of Liverpool, Liverpool, UK

### Abstract

Osteoarthritis (OA) is the most common condition to affect joints and a major cause of limitation and morbidity globally; affecting 528 million people. Diabetes is the most prevalent and rapidly increasing metabolic disorder in the world; in 2023 over 4.4 million people in the UK had diabetes with 90% having type 2 diabetes mellitus (T2DM). Conflicting evidence suggests individuals with T2DM have an increased risk of OA. Articular cartilage, whilst avascular, relies indirectly upon an adequate blood supply to the joint. If vessels become diseased in T2DM, it is postulated this can result in poor joint health leading to OA.

This study aims to evaluate whether changes in artery circumference, observed in T2DM, is related to the presence of OA in the knee joint.

Using data from the Osteoarthritis Initiative (OAI) , MRI knee scans have been used to measure popliteal artery luminal circumference. To account for the non-linear path taken by arteries, a 3D reconstruction was used to orientate slices in the correct plane for accurate measurement (Fig1 A, B). Average circumference is compared to Kellgren and Lawrence OA classification.

MRIs of patients with a BMI <30 and T2DM were compared to non-diabetic controls. Age, sex and BMI have all been considered to rule out confounding results.

Results (Fig1 C) show males with T2DM have a smaller lumen but does not relate to severity of OA. Females with T2DM have smaller lumen and is related to OA severity. OA severity is related to lumen size in women with T2DM but not seen in men suggesting there may be a possible link between T2DM and OA severity in women and could potentially be a target for therapeutic intervention.

With better understanding of this mechanism patients with T2DM can be advised to manage their condition better and be at less risk from poor joint health.

To validate this method, cadaveric knees were MRI scanned using this method then dissecting the artery. A 3D printed model of the artery and specific anatomical landmarks were used to identify where measurements were taken to verify the new method was more accurate (Fig1 D).

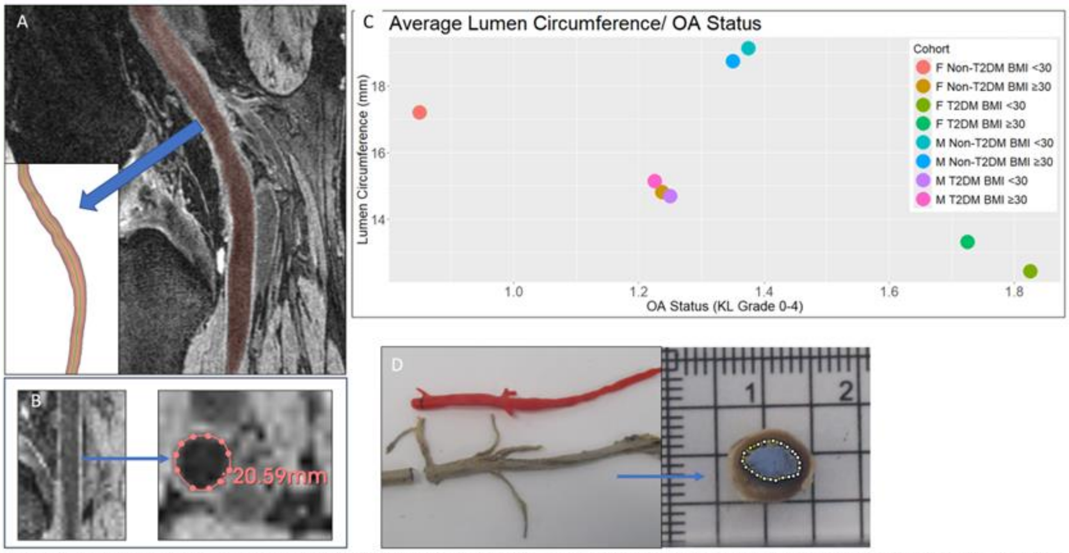

Fig 1. 3D Reconstruction of lumen used to orientate each MRI slice to centreline (A). Axial view of straightened volume used to measure lumen (B). Popliteal luminal circumference vs Kellgren Lawrence Grade of 160 patients (20 patients per cohort, left and right knee) (C). Popliteal artery was dissected and cut at specific landmarks to measure luminal circumference and verify accuracy of method (D)

**P46**

Withdrawn

### P47

### Advancements in Bone Mineral Density Assessment: A Review of The Role of Dual-Energy Computed Tomography and Artificial Intelligence

Dr. Hassan Alshamrani

Najran University, Najran, Saudi Arabia

### Abstract

Bone status is typically assessed using dual-energy absorptiometry (DEXA) and/or quantitative computed tomography (QCT). However, DEXA has the inherent limitation of not providing true volumetric bone mineral density (BMD), while QCT requires a dedicated CT machine and regular quality assurance. In recent years, there has been growing interest in using dual-energy computed tomography (DECT) to assess BMD, with the aid of artificial intelligence (AI). This narrative review summarises recent literature on this topic.

A systematic search was conducted across PubMed, Google Scholar, and Web of Science to identify relevant studies. The research found that DECT, when combined with AI, offers precise quantitative BMD measurements and enables simultaneous assessment of bone quality and composition. AI algorithms, particularly deep learning models, have demonstrated improved outcomes in automating the segmentation of bone regions in DECT images and predicting BMD. Additionally, convolutional neural networks (CNNs) have shown promise in analysing bone microarchitecture and predicting fracture risk.

Despite these promising developments, challenges remain, including the generalizability of AI models to different populations, the need for high-quality datasets for training, and the significant computational resources required to train robust AI models

**P48**

### Trabecular Bone Assembly Index: A Metric for Quantifying Trabecular Organisation and Connectivity

Dr Jonathan Williams

University of Strathclyde, Glasgow, UK

### Abstract

Trabecular bone structure is highly dynamic, reflecting the (im)balance between formation and resorption. Traditional morphometric parameters such as trabecular thickness (Tb.Th), separation (Tb.Sp), and connectivity density (Conn.D) provide valuable insights into bone microarchitecture but fail to capture the complexity of trabecular organisation as an emergent property of structural assembly. The Trabecular Bone (dis)Assembly Index (Tb.AI), a metric that (among other things) quantifies the number of assembly steps required to reconstruct a trabecular network from initial random seed points, is introduced here. This index extends beyond fractal and topological measures by considering the hierarchical formation of trabecular structures, with the stepwise (dis)assembly processes mimicking bone formation (and resorption).

This approach was applied to distinct high-resolution microCT datasets from small animal model bone samples to determine Tb.AI. The results (in progress) reveal that Tb.AI differentiates between types of trabecular networks, with structurally disorganised networks requiring fewer assembly steps than well-preserved, anisotropic trabeculae.

This study establishes Tb.AI as a tool for characterising trabecular bone architecture in preclinical contexts. Future work will explore its applicability to opportunistic clinical imaging data for osteoporosis screening.

### P49

### Impact of AI-enabled vertebral fracture (VF) identification on Fracture Liaison Service (FLS) key performance indicators (KPIs) and treatment recommendations varies by FLS

Dr Franz Clemano[1], Mrs Janine Connor[2], Dr Cassandra Chisholm[2], Mrs Jane Threlkeld[2], Mr Daniel Chappell[3], Professor Ken Poole[3], Dr Emma Gerety[4], Dr Jack Boylan[5], Dr Jane Turton[5], Professor Michael Stone[5], Mr Yotam Kimmel[6], Professor Opinder Sahota[7], Ms Rachel Eckert[8], Mr Tiago Santos[9], Dr Madeleine Sampson[9], Dr Mark Baxter[9], Dr Elizabeth Curtis[10], Professor Muhammad Javaid[1]

[1]University of Oxford, Oxford, UK. [2]Bradford Teaching Hospitals NHS Foundation Trust, Bradford, UK. [3]NIHR Cambridge Biomedical Research Centre, Cambridge, UK. [4]Cambridge University Hospitals NHS FT, Cambridge, UK. [5]University Hospital Llandough, Cardiff, UK. [6]Nanox-AI, Petach Tikva, Israel. [7]Nottingham University Hospitals NHS Trust, Nottingham, UK. [8]Oxford University Hospitals NHS Foundation Trust, Oxford, UK. [9]University Hospital Southampton NHS Foundation Trust, Southampton, UK. [10]MRC Lifecourse Epidemiology Centre, Southampton, UK

### Abstract

### Background

VF identification remains challenging for FLSs. While AI models identify adults with vertebral fractures from existing CT scans, the impact on wider FLS performance has not been evaluated.

### Objectives:

To compare the KPIs of FLSs before and after introduction of AI-enabled VF identification across FLSs in England and Wales.

### Materials and Methods:

The Nanox-AI HealthVCF AI model was implemented in 3 FLSs to identify potential vertebral fractures from existing CT scans with additional funding for FLS administrators and nurses. The KPIs for identification, assessment, treatment recommendation and follow-up were compared 12 months before and 6 to 12 months after AI implementation using the FLS-Database of England and Wales. Up to 4 control FLSs for each AI-FLSs were identified, matching on spine fracture identification KPI in 2023 to account for secular changes. Differences in proportions were compared using two-proportion z-tests.

### Results

The AI implementation increased VF case-finding 3 fold across sites ($p<0.001$) (Table). The higher numbers of patients with VF identified had a variable effect on the KPI for assessment, treatment recommendation and monitoring between FLS sites. In comparison, control sites demonstrated a smaller increment in in VF case finding 28.6 to 31.3% ($p=0.008$). For non-vertebral fractures (NVF) case-finding increased in AI-FLS (45.6 to 50.8%) but worsened in control FLSs (81.6 to 70.3%).

### Conclusion

As expected AI implementation significantly improved VF identification, but had differential effects on the KPI for VF and NVF between implementation sites. This finding highlights the importance of embedding service improvement for the whole patient pathway when implementing AI-enabled VF identification in the FLS setting.

Change in FLS-DB key performance indicators in the 12 months before and after the implementation of AI overall and by site for adults with VF.

| VF cases | All sites | | | FLS A | | | FLS B | | | FLS C | | |
|---|---|---|---|---|---|---|---|---|---|---|---|---|
| | Pre-AI | Post-AI | p-value | Pre-AI | Post-AI | p-value | Pre-AI | Post-AI | p-value | Pre-AI | Post-AI | p-value |
| KPI3 (%) | **36.2** | **137.3** | **-** | **132** | **314** | **-** | **11** | **41.8** | **<0.001** | **17.9** | **132.9** | **-** |
| KPI4 (%) | **74.2** | **35.3** | **<0.001** | **90.9** | **49** | **<0.001** | **45.8** | **15.2** | **<0.001** | **40.9** | **20.1** | **<0.001** |
| KPI5 (%) | 44.7 | 52.3 | 0.264 | 100 | 82.5 | 0.428 | 33.3 | 25.6 | 0.441 | 53.9 | 51.8 | 0.903 |
| KPI6 (%) | **21.7** | **11.9** | **<0.001** | 0 | 0 | - | **64.4** | **11.2** | **<0.001** | **62.4** | **32.4** | **<0.001** |
| KPI7 (%) | **69.2** | **42.1** | **<0.001** | **59.6** | **29.9** | **<0.001** | 94.9 | 98.7 | 0.0756 | **84.6** | **40** | **<0.001** |
| KPI8 (%) | **7.6** | **2.1** | **<0.001** | **8.8** | **1.9** | **0.002** | 0 | 0 | - | 8.7 | 4.4 | 0.15 |
| KPI9 (%) | 1 | 1.7 | 0.352 | 0.9 | 1.1 | 0.819 | 0 | 0 | - | 1.7 | 4.2 | 0.219 |
| KPI10 (%) | 23.8 | 12.2 | **<0.001** | **19.7** | **11.5** | **0.01** | **14.3** | **3.6** | **0.002** | **36.1** | **21.9** | **0.005** |
| Number of patients recommended treatment[1] | | | | 393 | 469 | | 56 | 221 | | 126 | 443 | |
| Additional number of patients recommended treatment (annualised) | | | | | 76 | | | 165 | | | 542[2] | |

Legend: KPI 3- Identification Spine fractures, KPI4 – Assessment within 90 days, KPI5 DXA within 90 days, KPI6 Falls risk assessment, KPI7 Recommended bone treatment, KPI8 Strength and Balance by 16 weeks, KPI9 16 weeks follow-up, KPI10 Treatment by 1st follow-up, p-values compared the pre- and post-AI pathway KPI values within each FLS site. These were calculated using two-proportion z-tests.

[1]Number of additional patients recommended treatment = KPI 2 * KPI 7 * local hip fracture count from NHFD.

[2]FLS C AI ran for 7 months so value extrapolated to reflect this.

### P50

### The 'three' ages of bone at the tissue level in ontogeny

Professor Peter Zioupos[1,2], Dr Andrea Bonicelli[3], Professor Elena Kranioti[4], Dr Bledar Xhemali[5]

[1]University of Hull, Kingston upon Hull, UK. [2]Cranfield University, Shrivenham, UK. [3]University of Central Lancashire, Preston, UK. [4]University of Crete, Heraklion, Crete, Greece. [5]Institute of Forensic Medicine, Tirana, Albania

### Abstract

In ontogeny the human skeleton follows different patterns in growing towards maturity and beyond it with respect to: (i) height/size; (ii) total skeletal mass; and (iii) bone properties, which are dependent on total bone mass and density/porosity. However, these properties are also concomitant to composition, architecture and physicochemical changes at the microscale. The micromechanical properties of bone at the tissue level have not been looked in detail and across the ages and an understanding of these patterns at the micro level may enable us to understand bone physiology in healthy ontogeny, in disease, as well as the developmental pressures placed upon it. We present here the nanomechanical properties of osteons and nearby interstitial tissue from samples of human ribs [R2] and clavicles [R3] from 85 individuals across a wide age range (12-85 yrs). The tests involved nanomechanical testing (CSM-NHT Switzerland) instrument, composition analysis (TGA) and physicochemistry (DSC, FTIR, XRD). The first pattern we observed was a two-phase behaviour of bone tissue with a rise in average hardness/stiffness up to the age of 35 and a decline thereafter. This behaviour, as expected, was related to the underlying chemistry and composition of the tissue [R2]. However, the data also showed that the interstitial lamellae also decline after maturity, while the prevailing wisdom [R4] is that residual tissue gros stiffer with age in-situ! The second pattern was revealed by examining the relative magnitude (difference) of values of interstitial and osteonal lamellae across the full age range. There was NO noticeable change to the age of ~57yr old and then there was significant decline beyond 57yrs. In conclusion a combined consideration of the compositional, mechanical and material data shows that bone tissue goes through 3 phases in ontogeny with cross over/threshold points at ~35 and ~57yrs old. The 1$^{st}$ phase is driven by modelling and maturing of the tissue up to age 35. The 2$^{nd}$ phase up to 57yrs is a phase for maintaining equilibrium. The 3$^{rd}$ phase post-57yrs does not appear to be physicochemical in its origin, but simply a change in the relative ratios of bone resorption-vs-formation (removal-vs-addition of osteons).

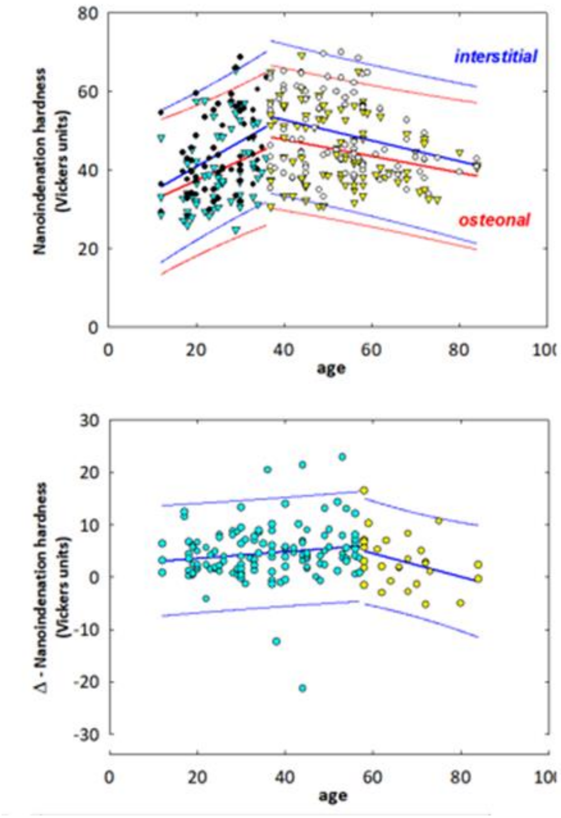

Figure 1: (TOP) Behaviour of Hardness (in Vickers units for easy comparisons) in the rib material for interstitial (Blue) and osteonal (Red) lamellae. Interstitial lamellae were on average (across ages) 4.3(±5.2) VU harder and 2.3(+1.7) GPa stiffer than the osteonal ones. The dataset showed just one inflection point at 35yrs of age using CR (component + residuals) plots to identify the threshold. At this point the tissue transitioned from the growing-maturing-modelling phase to the developed-mature-remodelling phase. There was an upward trend in phase-I and afterwards a downward trend in phase-II.

(BOTTOM) Behaviour of the relative magnitude (difference) of values of interstitial and osteonal lamellae across ages up to 84 yrs in nanoindentation Hardness. The dataset did NOT at this point show any significant or noticeable change up to the age of 57yrs old and then it showed significant decline in the difference for both Hardness (and also Stiffness) values. The nanoindentation hardness-vs-modulus data showed the same linear dependency throughout life and in male/females, and on ribs/clavicles and across ages.

References: [1] Currey, J.D., *Bones:Structure and Mechanics*, Princeton Univ Press, 2013. [2] Bonicelli A. *et al*., *Bone*, vol 155, 116265 (2022) [3] McGivern H. *et al*., *Front. Bioeng. Biotechnol*. Vol 7:467 (2020) [4] Burr DB, & Allen MR (eds) *Basic and Applied Bone Biology*, Academic Press, 2019.

**P51** (Also presented as an Oral Communication in Clinical Cases Session)

### Central obesity measured by waist to height ratio vs. BMI-derived obesity as predictors of fracture risk: A retrospective cohort study from Oswestry, UK

Dr Chadi Rakieh[1], Dr Jan-Herman Kuiper[2], Dr Claire Mennan[1], Dr Diane Powell[1], Dr Shu Ho[1], Dr Bernhard Tinns[1], Professor Kassim Javaid[3]

[1]The Robert Jones & Agnes Hunt Orthopaedic Hospital Foundation Trust, Oswestry, UK. [2]Keele University, Newcastle-under-Lyme, UK. [3]The University of Oxford, Oxford, UK

### Abstract

#### Background and Aims

The relationship between obesity and fracture risk is complex and inconclusive. This study aimed to determine whether: (1) central obesity, measured by waist-to-height ratio (WHtR), predicts recent fragility fracture within two years; (2) WHtR improves fracture risk prediction after accounting for the components (excluding BMI) that constitute the FRAX score; and (3) WHR influences fracture site risk differently to body mass index (BMI).

#### Methods

This retrospective study included postmenopausal women and men over 50 years of age who attended our bone density service between March and July 2023, with a BMI between 18.5 and 40. The primary outcome was prevalent fracture within two years. Univariable multinomial regression was used to analyse the influence of WHtR and BMI on fracture risk. Multivariable multinomial regression assessed whether WHtR or BMI improved fracture risk prediction after adjusting for the ten components (excluding BMI) that constitute FRAX: age, sex, femoral neck bone mineral density (BMD), prior fragility fracture, parental history of hip fracture, current smoking, long-term oral glucocorticoid use, rheumatoid arthritis, other causes of secondary osteoporosis, and alcohol consumption of ≥3 units daily. A two-sided p-value <0.05 was considered statistically significant.

#### Results

A total of 1,005 participants were eligible (83.9% female, mean age 71±10.8 years), of whom 30.1% had a recent fracture, including 5.3% hip, 10.7% vertebral, and 7% wrist fractures. Before adjusting for FRAX components, BMI showed no correlation with fracture risk, whereas WHtR ≥0.6 was significantly associated with increased fracture risk (odds ratio [OR] 1.59, 95% confidence interval [CI] 1.04–2.48, p=0.037). After adjusting for FRAX, BMI ≥30 was associated with increased fracture risk (OR 1.65, 95% CI 1.10–2.49, p=0.016), but to a lesser extent than WHR ≥0.6, which showed a stronger association (OR 2.48, 95% CI 1.53–4.11, p<0.001). Figure 1 indicates that individuals with increased central obesity had a significantly higher risk of vertebral fractures, with a tendency towards increased hip fracture risk, whereas those with overweight BMI exhibited an opposite trend at these skeletal sites.

#### Conclusions

Central obesity, as measured by WHtR, appears to be a stronger predictor of fracture risk than BMI. WHtR and BMI demonstrate distinct fracture risk patterns across skeletal sites, with WHtR being more strongly associated with vertebral and hip fractures. Further research is required to validate these findings in longitudinal cohorts and assess the added value of central obesity in FRAX-based fracture risk prediction.

Figure 1. Distribution of fracture across different skeletal sites according to increased central obesity (WHtR≥0.5) and overweight (BMI≥25)

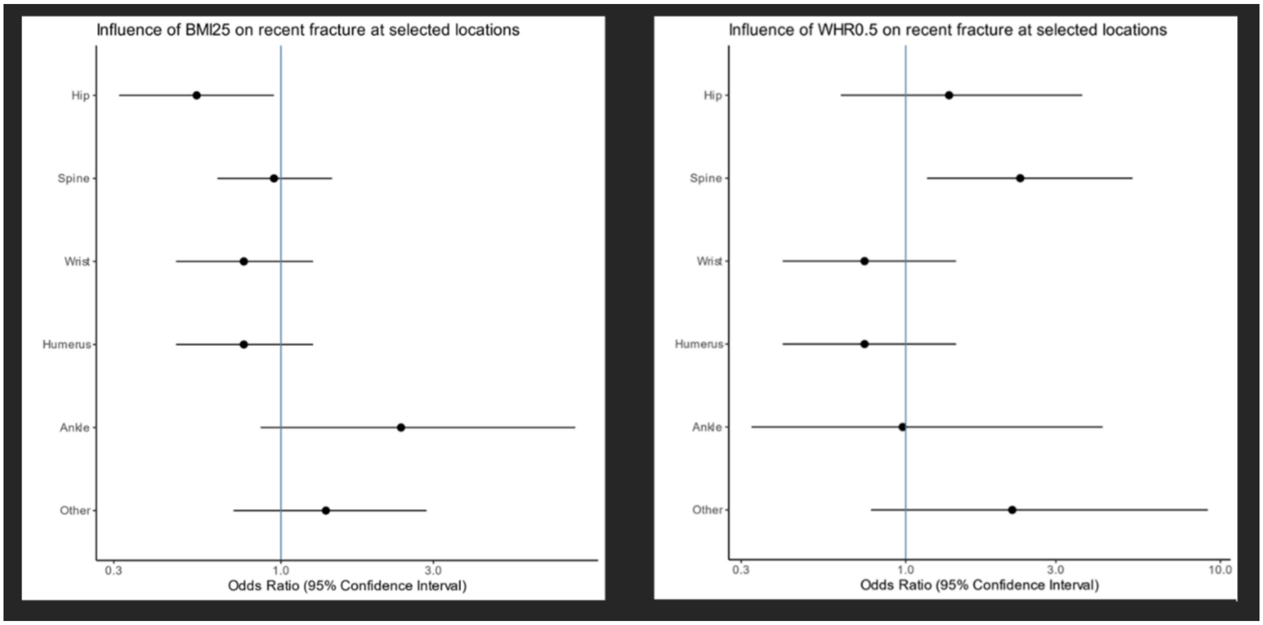

### P52

### Picking up Hidden Osteoporosis Effectively during Normal CT Imaging without additional X-rays (PHOENIX-f): Results from the 1-year Extension Phase

Mr Daniel Chappell[1], Dr Rahul Shah[1], Dr Jane Fleming[1], Ms Judith Brown[1], Professor Emma Clark[2], Professor Lee Shepstone[3], Professor Thomas Turmezei[3], Dr Adam Wagner[3], Ms Karen Willoughby[1], Dr Stephen Kaptoge[1], Professor Ken Poole[1]

[1]University of Cambridge, Cambridge, UK. [2]University of Bristol, Bristol, UK. [3]University of East Anglia, Norwich, UK

### Abstract

The PHOENIX (Picking up Hidden Osteoporosis Effectively during Normal CT Imaging without additional X-rays) one-stop osteoporosis screening service targets patients attending hospital for diagnostic computed tomography (CT) scans. Bone mineral density (BMD) is measured and vertebral fractures identified re-using those CT scans of patients assessed as at moderate/high risk of fracture, with subsequent osteoporosis investigations and treatment recommended by either electronic letter to GPs or hospital Metabolic Bone services.

To improve identification and treatment rates among higher fracture risk older patients, we conducted a randomised controlled feasibility trial involving one teaching hospital and four regional hospital radiology departments in England, approaching 595 patients (women aged ≥65, men ≥75) undergoing CT scans of abdomen and/or pelvis. Patients in CT waiting areas were offered a FRAX 10-year fracture risk questionnaire, with embedded consent form. Consenting patients categorised into higher risk categories (FRAX red/amber zones) were randomised (1:1:1) to: 1) PHOENIX one-stop osteoporosis screening service; 2) FRAX results sent to GP only; and 3) usual care (GP informed of participation).

The trial recruited/randomised 382 patients within 10 months, with 276 of 327 survivors (84%) completing 1-year follow-up (95% CI: 81–88). Osteoporosis was diagnosed in 41% of 362 analysable CT scans, and vertebral fractures found in 20% (n=53/264). 36%, 27%, and 8% of those patients recommended osteoporosis drug treatment in the PHOENIX, FRAX-only, and usual care groups respectively reported taking treatment at follow-up.

To investigate optimising treatment following identification, a trial extension additionally consented 250 patients to the PHOENIX identification path, with randomisation (1:1) to subsequent treatment led by : A) GPs; B) hospital Metabolic Bone clinics. Here, 26% of Group A and 24% in Group B reported taking osteoporosis treatment at follow-up. Group B patients were greatly delayed in being seen due to service pressures on all appointments.

Recruitment and follow-up were sufficient for a definitive trial. Given the high rate of vertebral fractures identified, the definitive trial is likely to focus on these patients.

### P53

### Secondary osteoporosis prevention in the 80 years old and over population: Equity of care

Miss Pritika Anthonypillai[1], Ms Amara Williams[2], Ms Sophie Maggs[2], Ms Linda Scanlon[2], Dr Chris Edwards[2], Dr Inder Singh[2]

[1]Cardiff University, Cardiff, UK. [2]Aneurin Bevan University Health Board, Ystrad Mynach, UK

### Abstract

**Introduction:**

Fragility fractures increase the risk of re-fracture and mortality, especially within two years. Fracture Liaison Services (FLS) aim to prevent secondary fractures by ensuring quality care for patients over 50. This study assesses equity of care in an existing FLS for patients above and below 80 years and evaluates re-fracture and mortality outcomes.

**Methods:**

A retrospective review of 1,099 patients seen by the Aneurin Bevan Fracture Liaison Service (AB-FLS) from July to December 2023 using national FLS Database (FLS-DB) data was completed. After excluding 10 patients with missing data, 1,089 were categorized into two age groups: below and above 80. Data on previous fractures, re-fractures, and fracture type (hip/femur, spine, wrist, humerus, pelvis, others) was collected. Patients were followed for up to 18 months until December 31, 2024, to assess re-fractures and mortality.

**Results:**

The cohort's mean age was 79 years (range: 50–102), with a significant female predominance (76.8%, $p<0.001$). Prior fractures were recorded in 53% (n=577) of patients, with a mean duration of 5 years (range: 0–36). Most (93.6%) lived in the community, while 6.4% were in care homes.

The AB-FLS reviewed 534 (49%) patients aged 50–80 and 555 (51%) aged over 80, with no significant group differences. Female distribution was similar (50.3% vs. 49.6%).

Bone treatment was initiated in 621 (57%) patients. Over 18 months, 916 (84%) had no re-fracture, while 173 (15.8%) did, with a mean time of 203 days (range: 2–538).

Re-fracture rates were significantly higher in patients over 80 (61.3%, n=106) than under 80 (38.7%, n=67, $p<0.0001$). Mean time to re-fracture was similar: 207 days (7–528) for under 80 and 200 days (2–513) for over 80.

Over 18 months, 224 patients had died. One-year mortality was 18.3% (n=199), significantly higher in those over 80 (74.8%, n=149) than under 80 (25.1%, n=50, $p<0.0001$).

**Discussion:**

This study showed that re-fractures and mortality were significantly higher in patients over 80, despite similar time to re-fracture between age groups. These findings highlight the need for targeted interventions to reduce fracture risk and improve outcomes in older patients.

**Conclusion:**

The AB-FLS has demonstrated equitable care over the six-month period; however, further assessment over a longer timeframe is needed for confirmation. Given the significantly higher risk of re-fracture and mortality in older patients, secondary fracture services should be tailored to better address the needs of this population, ensuring true equity in healthcare.

Table 1: Baseline characteristics

| | | All patients | 50-80 years | Above 80 years | P-value |
|---|---|---|---|---|---|
| n (%) | | 1089 | 534 (49%) | 555 (51%) | 0.35 ns |
| Mean age in Years (range) | | 79 (50 – 102) | 71 (50 – 80) | 87 (80 – 102) | <0.001 |
| Females; n | | 836 | 421 | 415 | ns |
| Admitted from care home n | | 70 | 9 | 61 | <0.0001 |
| **H/o prevalent fracture** | Total | 577 | 262 (45.4%) | 315 (54.59%) | 0.0018 |
| | Hip/Femur; n (%) | 72 | 26 (36.11%) | 46 (63.89%) | 0.009 |
| | Spine; n (%) | 143 | 52 (36.36%) | 91 (63.64%) | <0.0001 |
| | Wrist; n (%) | 76 | 38 (50.0%) | 38 (50.0%) | 1.0 ns |
| | Humerus; n (%) | 44 | 25 (56.82%) | 19 (43.18%) | 0.2 ns |
| | Pelvis; n (%) | 26 | 5 (19.23%) | 21 (80.77%) | <0.0001 |
| | Others; n | 216 | 116 (53.70%) | 100 (46.30%) | 0.124 ns |
| **Index Fracture type** | Total fragility fractures; n (%) | 1089 | 534 (49.04%) | 555 (50.96%) | 0.37 ns |
| | Hip/Femur; n (%) | 431 | 154 (35.73%) | 277 (64.27%) | <0.0001 |
| | Spine; n (%) | 211 | 109 (51.66%) | 102 (48.34%) | 0.5 ns |
| | Wrist; n (%) | 265 | 179 (67.55%) | 86 (32.45%) | <0.0001 |
| | Humerus; n (%) | 77 | 53 (68.83%) | 24 (31.17%) | <0.0001 |
| | Pelvis; n (%) | 70 | 20 (28.57%) | 50 (71.14%) | <0.0001 |
| | Others; n (%) | 35 | 19 (54.29%) | 16 (45.71%) | 0.47 ns |
| % On bone sparing treatment at initial fracture, n (%) | | 136 | 56 (41.18%) | 80 (58.82%) | 0.004 |

### P54

### Comparison of using AI to augment routine radiology reports vs asynchronous clinical confirmation of AI flagged scans with potential vertebral fractures

Ms Jane Threlkeld[1], Ms Janine Connor[1], Dr Cassandra Chisholm[1], Professor Muhammad Javaid[2]

[1]Bradford Teaching Hospitals NHS Foundation Trust, Bradford, UK. [2]University of Oxford, Oxford, UK

### Abstract

OBJECTIVES: To compare the performance of using synchronous AI-augmented general radiology reporting with asynchronous dedicated MSK expert review of CT scans flagged by AI.

METHODS: Using the Nanox-AI HealthVCF AI model, CT scans that included the thoracic/ lumbar spine were re-analysed at the high specificity setting for moderate/ severe vertebral fractures. Flagged CT images were annotated for the reporting radiologist to confirm and the patient referred to the Fracture Liaison Service (FLS). In parallel, all AI-flagged CT scans were sent to a separate server for asynchronous reading by a musculoskeletal (MSK) radiologist. We used Fisher's exact test to compare the two pathways.

RESULTS: Between December 2023 and November 2024, 10,679 scans were analysed, 1,311 flagged by the AI as potential vertebral fractures. An MSK radiologist confirmed 946 (72.2%) as vertebral fractures. 20.9% of flagged scans with at least one confirmed vertebral fracture were not reported and referred. Trainees had the lowest missing rate, with the highest missing rate being for outsourced and locums radiologists ($p<0.001$) (Fig). The highest non-MSK missing rates were from Chest/Nuclear Medicine (29.6%), head&neck (26.7%) and Breast (26.3%).

CONCLUSION: Even with AI-informed prompting, a fifth of scans with confirmed VF were not reported. This supports asynchronous reading of the AI-flagged scans when implementing in the FLS setting.

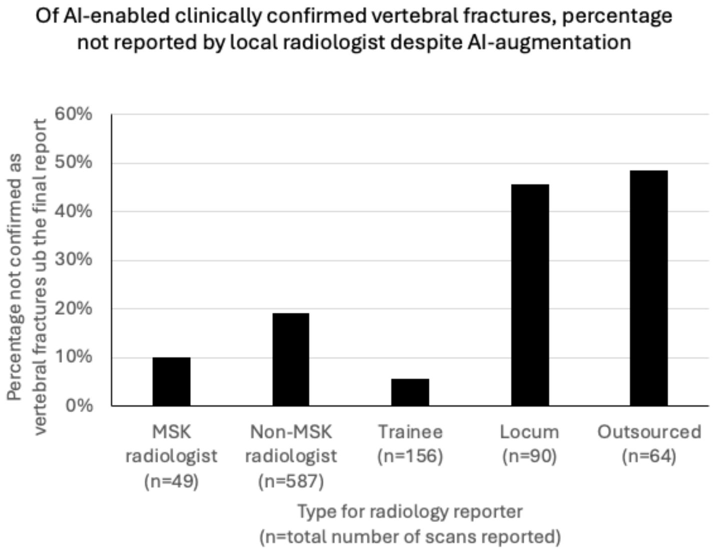

### P55

### Long-Term Effects of Teriparatide Therapy Followed by Antiresorptive Treatment on Bone Mineral Density in Patients with Osteoporosis

Dr Kapil Kumar Garg[1], Dr Anupama Nandagudi[1,2]

[1]Basildon University Hospital, Mid & South Essex NHS Foundation Trust, Basildon, UK. [2]Anglia Ruskin University, Faculty of Health, Medicine and Social Care, Chelmsford, UK

### Abstract

**Introduction**
Teriparatide treatment is recommended for 24 months followed by switching to an anti-resorptive agent to prevent bone loss as per NICE and NOGG guidelines.

**Objective**
To analyse the effects of teriparatide treatment and role of further sequential treatment in patients with osteoporosis as per NICE and NOGG guidelines.

**Methods**
A 12 year follow-up (2012-2024) was done in patients with osteoporosis after their treatment with teriparatide and total of 46 patients identified for analysis. Demographics, baseline and post-treatment T-scores of all patients on teriparatide and further sequential treatments were compared using DEXA scan at our centre and adverse effects were analysed.

**Results**
Out of 46 patients, 2 patients declined treatment, and one could not inject teriparatide. There was premature discontinuation of teriparatide in 10 patients (between 1-7 months). We reviewed data from remaining 33 patients. Mean age at teriparatide initiation was 70.3 years (range 56-82) which included 29 post-menopausal females (87.8%). Twenty-nine patients (87.8%) completed full 24 months of teriparatide treatment, and 4 patients (12.2%) had premature discontinuation of therapy (between 12-18 months). Mean duration of treatment was 22.9 months. Teriparatide was initiated as first-line therapy in 15 patients (45.5%), second-line in 14 patients (42.4%) and third-line treatment in 4 patients (12.1%). Vertebrae were site of fragility fracture before teriparatide in 20 patients (60.6%), wrist in 9 patients (27.3%), humerus and neck of femur in 5 (15.2%) each. Minor adverse effects were reported in 13 patients (39.4%) not requiring change in teriparatide treatment.
Out of 33 patients who received teriparatide, 28 patients received sequential osteoporosis treatment with denosumab in 11 patients (39.3%), zoledronic acid in 10 patients (35.7%) and Alendronic acid in 7 patients (25%). 5 patients did not receive any further specialized treatment. The mean duration of sequential treatment was 3.56 years prior to repeat DEXA scan.

Rate of change in T-score after teriparatide therapy was +12.95% at lumbar spine (Table 1) and +2.1% at total hip (Table 2) and not significant at neck of femur (Table 3). Further changes in T-score with sequential denosumab, zoledronic acid and alendronic acid at each site is illustrated in tables 1-3.

| Table 1. Lumbar Spine | | | | | | | | |
|---|---|---|---|---|---|---|---|---|
| T-score Before teriparatide initiation (A) | T-score Post Teriparatide treatment (B) | Rate of change (B-A) | Statistical Significance | Second Sequential agent after teriparatide | Treatment duration (average) | T-score (C) | Rate of change (C-B) | Statistical Significance |
| -4.13 | -3.41 | +12.95% | Significant | All treatments combined (28 patients) | 3.56 years | -3.04 | +2.63% | Not significant |
| -4.02 | -3.24 | +12.89% | Significant | Denosumab (11 patients) | 2.91 years | -2.50 | +7.81% | Significant |
| -3.98 | -3.31 | +12.79% | Significant | Zoledronic acid (10 patients) | 4.70 years | -3.03 | +1.3% | Not significant |
| -4.16 | -3.54 | +12.11% | Significant | Alendronic acid (7 patients) | 5.57 years | -3.67 | -6.4% | Significant |

| Table 2. Total Hip | | | | | | | | |
|---|---|---|---|---|---|---|---|---|
| T-score Before teriparatide initiation (A) | T-score Post Teriparatide treatment (B) | Rate of change (B-A) | Statistical Significance | Second Sequential agent after teriparatide | Treatment duration (average) | T-score (C) | Rate of change (C-B) | Statistical Significance |
| -2.60 | -2.39 | +2.1% | Not significant | All treatments combined (28 patients) | 3.56 years | -2.12 | +2.46% | Not significant |
| -2.55 | -2.38 | +0.51% | Not significant | Denosumab (11 patients) | 2.91 years | -2.01 | +0.56% | Not significant |
| -2.35 | -2.12 | +2.36% | Not significant | Zoledronic acid (10 patients) | 4.70 years | -2.04 | +4.76% | Significant |
| -2.29 | -2.06 | +5.9% | Significant | Alendronic acid (7 patients) | 5.57 years | -2.37 | +2.6% | Not significant |

| Table 3. Neck of Femur | | | | | | | | |
|---|---|---|---|---|---|---|---|---|
| T-score Before teriparatide initiation (A) | T-score Post Teriparatide treatment (B) | Rate of change (B-A) | Statistical Significance | Second Sequential agent after teriparatide | Treatment duration (average) | T-score (C) | Rate of change (C-B) | Statistical Significance |
| -2.71 | -2.62 | | Not significant | All treatments combined (28 patients) | 3.56 years | -2.40 | | Not significant |
| -2.67 | -2.48 | | Not significant | Denosumab (11 patients) | 2.91 years | -2.04 | | Not significant |
| -2.48 | -2.70 | | Not significant | Zoledronic acid (10 patients) | 4.70 years | -2.46 | | Not significant |
| -2.66 | -2.80 | | Not significant | Alendronic acid (7 patients) | 5.57 years | -2.75 | | Not significant |

### Conclusion

The main limitation of our study small number of patients. Teriparatide treatment was well tolerated and showed significant improvement in lumbar spine while maintaining bone mineral density at hip and neck of femur. Denosumab proved a better choice as sequential agent at lumbar spine and zoledronic acid was superior at hip. Alendronate acid could not maintain bone mineral density at lumbar spine but did maintain bone mineral density at hip.

### P56

### Effect of Renal Function on Procollagen Type I N-Terminal Propeptide (PINP) and Bone Alkaline Phosphatase (BAP) Suppression in Denosumab-Treated Osteopootic Patient

Dr Diane Powell, Dr Claire Mennan, Mrs Tracey Roberts, Mrs Julie Cole, Dr Chadi Rakieh

Robert Jones & Agnes Hunt Orthopaedic Hospital NHS Foundation Trust, Oswestry, UK

### Abstract

Background:

Intact (PINP) is unaffected by renal function, making it a potential biomarker for assessing treatment response in patients with impaired renal function.

Aims:

The aim of this study was to determine whether chronic kidney disease (CKD) stage influences the suppression of PINP and BAP below the premenopausal mean.

Methods:

Data from 114 postmenopausal women with osteoporosis attending a hospital metabolic bone unit for denosumab treatment was analysed. PINP and BAP were measured using an i-SYS autoanalyzer (IDS Ltd) following standard protocol.

Results:

No significant differences were observed in age, vitamin D, total alkaline phosphatase, or calcium between the different CKD groups (Table1). All patients had received at least one prior denosumab treatment, with no significant difference in treatment duration across groups.

Although not reaching statistical significance, PINP levels exhibited a trend toward increasing with declining renal function. BAP levels significantly increased with declining renal function ($p=0.032$). The proportion of subjects whose turnover was suppressed below the premenopausal mean for PINP (<30.1ng/ml) and BAP (<9.8 ng/mL) declined from CKD stage 2 to CKD stage 4, though this difference did not reach statistical significance.

Conclusions:

These results indicate that denosumab suppresses PINP levels to a greater extent than BAP across all CKD groups examined (stages 2-4). Furthermore, there is a trend suggesting that patients with lower renal function experience less effective suppression of bone turnover. The relationship between bone turnover suppression and bone mineral density response requires further evaluation.

**Table 1: Characteristics of Denosumab Patients by CKD Status**

| Characteristic | CKD2 (n = 53) | CKD3a (n = 24) | CKD3b (n = 27) | CKD4 (n = 10) | p-value |
|---|---|---|---|---|---|
| Age (years) | 82.3 ± 5.4 | 84.4 ± 5.7 | 84.0 ± 6.1 | 82.3 ± 5.7 | 0.390 |
| PINP (ng/ml) | 19.9 [15.1–25.2] | 23.1 [16.9–32.7] | 23.6 [15.9–33.2] | 31.4 [17.7–43.6] | 0.204 |
| % PINP < 30.1 | 79% | 67% | 63% | 50% | 0.119 |
| PTH (pmol/L) | 3.5 [3.1–5.1] | 5.5 [4.9–8.7] | 6.3 [4.1–7.2] | n.a. | 0.015 |
| Vitamin D (nmol/L) | 83.4 [73.7–102.0] | 75.9 [64.3–94.7] | 88.1 [70.4–108.0] | 91.3 [80.6–97.8] | 0.276 |
| eGFR (mL/min/1.73m²) | 75.0 [67.0–83.0] | 51.0 [47.5–55.0] | 37.0 [34.0–41.0] | 24.5 [20.0–26.0] | <0.001 |
| Total AP (U/L) | 69.5 [57.5–80.5] | 65.0 [50.0–78.0] | 70.0 [58.0–82.0] | 77.0 [55.0–97.0] | 0.705 |
| BAP (ng/ml) | 9.9 [8.6–13.4] | 9.5 [7.9–13.4] | 10.8 [7.0–13.5] | 17.0 [12.8–19.5] | 0.032 |
| BAP < 9.8 | 50% | 57% | 33% | 0% | 0.055 |
| Calcium (mmol/L) | 2.43 ± 0.10 | 2.43 ± 0.10 | 2.44 ± 0.10 | 2.40 ± 0.14 | 0.750 |

**P57**

Withdrawn

### P58

### Prevalence of normal, low and very low BMD in Artificial Intelligence (AI) -identified vertebral fractures (VF)

Mr Tiago Santos[1], Dr Madeleine Sampson[1], • Dr Mark Baxter[1], Dr Elizabeth Curtis[2], Professor Muhammad Javaid[3]

[1]University Hospital Southampton NHS Foundation Trust, Southampton, UK. [2]MRC Lifecourse Epidemiology Centre, Southampton, UK. [3]University of Oxford, Oxford, UK

### Abstract

### Background

AI-models effectively identify patients with VF from existing CT scans. Access to anabolic osteoporosis therapy is often dependent on the lowest T score but the proportion of these adults with low and very low BMD is unknown.

### Purpose

To describe BMD in women and men aged over 50 years with AI-identified VF.

### Methods

Consecutive CT scans in adults aged 50 years plus were analyzed using the Nanox HealthVCF algorithm. AI flagged scans were then clinically confirmed and patients ≤ 75 years were referred to DXA scanning. Older patients were referred if they had multiple fractures. Logistic regression was used to predict BMD categories ≤-2.5 or ≤-3.5.

### Results

From 01/08/23 - 04/12/2024, 11,092 CT scans were sent for AI analysis. Of the 3,741 scans flagged by the AI with potential VF, 1,336 were clinically confirmed as moderate or severe VF. Of the first 218 referred and completed a DXA, 82/218 (37.6%) had a lowest T score ≤-2.5 and 13/218 (6.0%) had a T score of ≤-3.5. 36/127 (28.4%) of men with clinically confirmed VF had a T score ≤-2.5 and 3.9% of men a T score ≤-3.5%. A third of women and 41.2% of men had a lowest BMD between -2.4 and -1 and 16.5% and 30% had a T score > -1 respectively (Figure). Age at time of CT scan did not predict BMD categories (p=0.1).

### Conclusion

Approximately half of women and 30% of men with a confirmed AI-identified VF had a T score of ≤-2.5. A significant proportion of older adults with moderate/ severe vertebral fractures had normal BMD and further work is required to establish their treatment needs.

Relationship between Age at time of CT scan and Lowest T score in clinically confirmed moderate/ severe vertebral fractures identified using AI

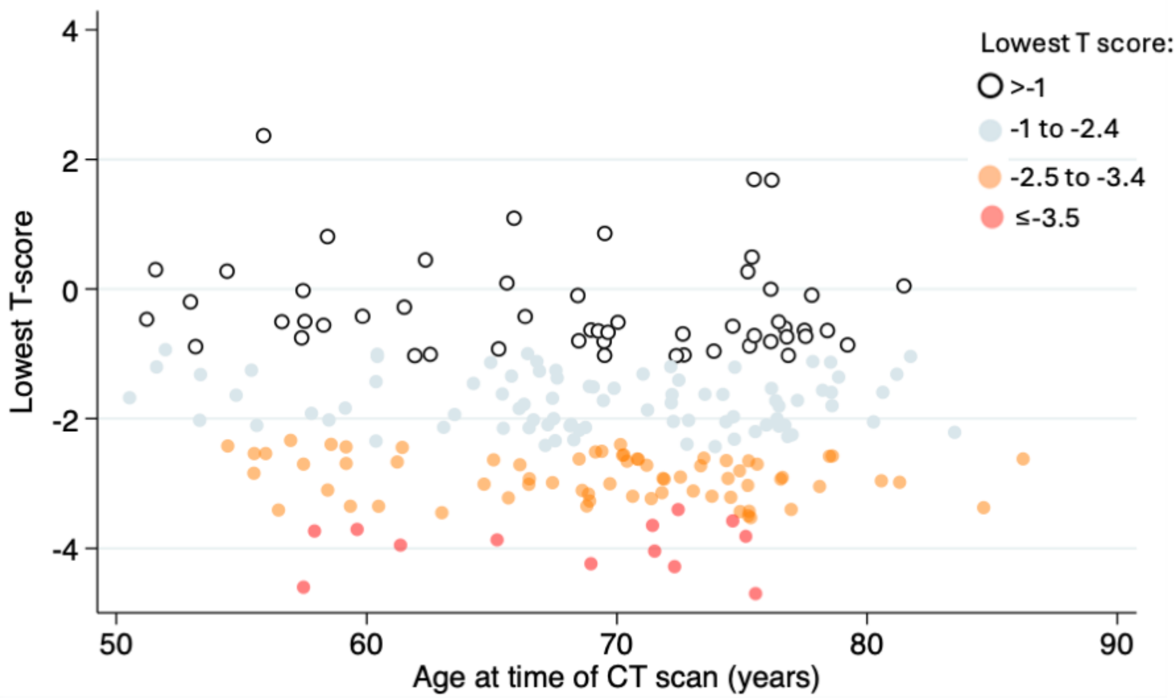

### P59

### Delivering romosozumab for high fracture risk patients in the Fracture Liaison Service (FLS) setting: service modifications and early results

Dr. Claudia Irene Maushart[1], Ms Rachel Eckert[2], Ms Sarah Connacher[2], Prof. M Kassim Javaid[1]

[1]Nuffield Department of Orthopaedics, Rheumatology and Musculoskeletal Sciences, University of Oxford, Oxford, UK. [2]Metabolic Bone Unit, Oxford University Hospital, Oxford, UK

### Abstract

### Objective
The UK guidelines recommend first line treatment with romosozumab (Romo) for postmenopausal people with a recent hip, vertebral, humerus or wrist fracture and a BMD T-score ≤-3.5 SD or T-score ≤-2.5 SD with a vertebral fracture (VF) within 24 months, a history of ≥2 osteoporotic VFs, or a very high FRAX-based fracture risk. These patients are routinely seen by FLS where anabolic treatment usually requires referral to the specialist bone clinic.

### Methods
A patient co-developed, multidisciplinary team (MDT) identified changes to the FLS pathway to enable access to Romo. These modifications were implemented to effectively identify high-risk patients: Fractures below the knee were excluded, and spine fracture detection enhanced by standardising referral from positive vertebral fracture assessments (VFA) and AI analyses of existing CT scans. All patients without a history of cardiovascular disease were automatically referred for DXA and completed a questionnaire including QRISK3 to evaluate their cardiovascular risk factors. If eligible by DXA, patients were sent lab tests (including HbA1c, non-fasting lipids) and the Royal Osteoporosis Society Romo leaflet before their FLS Nurse telemed assessment. Eligible cases were reviewed in weekly MDT meetings with the clinical lead. Treatment was initiated using homecare providers.

### Results

From January 2024 to September 2024, the FLS identified a total of 1705 postmenopausal women with newly diagnosed fractures, including major osteoporotic fractures of the hip (n=361), humerus (n=193), spine (n=376), or wrist (n=232). After the complete clinical assessment, Romo was started in 61 patients (3.6%). Almost half of these patients had a vertebral fracture (n=30) and nearly a third a wrist fracture (n=19, Figure 1). The median time from first fracture to treatment recommendation was 147 (IQR 104-194) days and from fracture to Romo initiation was 170 (IQR 119-209) days.

Patients starting romosozumab by index fracture

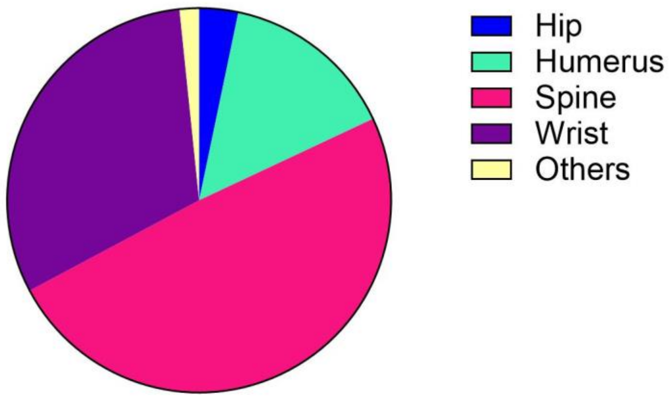

**Conclusion**

Integrating Romo into the FLS setting required a pathway redesign for patient identification, assessment, treatment recommendation and monitoring. Further work is required to assess clinical and patient experience and satisfaction.

### P60

### Primary Prevention. Fracture Risk and Osteoporosis in Women: A Practice Population Study

Ms Jodie Wilkinson, Mr Conor Corbett

Sabden and Whalley Medical Group, Whalley, UK

### Abstract

*Introduction*

Fracture risk and osteoporosis increase with age [1,2]. Over half a million new fragility fractures occur annually in the UK, with associated significant implications in terms of patient disability and cost to the health system. Bisphosphonate treatment can reduce the relative risk of fracture by 20-60% [3] at various anatomical sites.

*Aims*

To prospectively assess the bone health of practice female patients aged 60-65 years. To provide lifestyle advice on maintaining bone health to those not meeting criteria for objective dual energy x-ray absorptiometry (DEXA) assessment. To prescribe appropriate treatment for those patients newly found to have osteopenia or osteoporosis.

*Method*

The project occurred between January 2023 and June 2024; predominantly undertaken by a pharmacy technician with supervision from the practice pharmacist. Patients were identified using EMIS searches. Pre-existing osteopenia or osteoporosis was excluded, as were patients who had DEXA scans in the previous 5 years. A bespoke, modified FRAX assessment [2] was text to patients using AccuRx. Text responses were entered through FRAX. Patients with FRAX scores exceeding 10% for major fracture were referred for DEXA scans. Those not crossing the DEXA threshold were informed and text Royal Osteoporosis Society bone health advice.

*Results*

Five hundred and eighty-two women were identified. Responses were received from 366 (62.9%) women. One hundred and four (28.4%) respondents met criteria for DEXA. Forty-one of those referred were newly diagnosed with osteopenia and twenty with osteoporosis. FRAX sensitivity was 58.6% for a new bone health diagnosis.

*Conclusion*

A response rate over 60% led to 1 in 6 respondents (61/366) receiving a new diagnosis. These patients have now started appropriate medical treatment which should reduce their future fracture risk [4]. Twenty patients (100%) diagnosed with osteoporosis agreed to start oral bisphosphonates. Feedback was positive from patients and clinicians. The project was an excellent use of multi-disciplinary resources and digital technology within primary care, with potential to be easily expanded across boundaries.  Limitations were the requirement that GPs send DEXA referrals and that at present this is unpaid work in primary care.

*References*

1.  National Osteoporosis Guideline Group (NOGG) (n.d.) *NOGG 2021: Clinical guideline for the prevention and treatment of osteoporosis*. Available at: https://www.nogg.org.uk/full-guideline (Accessed: 24 October 2024)

2.  University of Sheffield (n.d.) *FRAX tool*. Available at: https://www.frax.shef.ac.uk/FRAX/tool.aspx?country=1 (Accessed: 24 October 2024)

3. Reconsidering the Benefits of Osteoporosis Treatment: The Case of Bisphosphonates. Goodman, Christopher W. The American Journal of Medicine, Volume 137, Issue 6, 476 – 478

4. Screening in the community to reduce fractures in older women (SCOOP): a randomised controlled trial. Shepstone, Lee et al. The Lancet, Volume 391, Issue 10122, 741 - 747

### P61

### Assessment and Management of Patients with Low Bone Mineral Density (BMD) and Coeliac Disease (CeD): A Clinical Audit

Dr Kapil Kumar Garg[1], Dr Anupama Nandagudi[1,2]

[1]Basildon University Hospital, Mid & South Essex NHS Foundation Trust, Basildon, UK. [2]Anglia Ruskin University, Faculty of Health, Medicine and Social Care, Chelmsford, UK

### Abstract

### Background:

The prevalence of low BMD with CeD is variable and has been reported in >50% of new CeD diagnosis. The aim of our audit was to evaluate management of patients with low BMD and CeD at our centre in line with NICE and British Society of Gastroenterology (BSG) guidelines.

### Methods:

A retrospective audit of patients reviewed in osteoporosis service at our centre between 2005-2024 with low BMD and confirmed diagnosis of CeD by gastroenterologist was carried out. Patients with CeD and normal BMD were excluded from the study.

### Results:

Of 51 patients analysed, mean-age at diagnosis of CeD was 58.51years (range 16-84) and included 47 females (92.2%) with 35 being postmenopausal (68.6%). Mean duration between diagnosis of CeD and low BMD was 1.78 years with CeD diagnosed earlier in 29 patients (56.9%). Both conditions were diagnosed concurrently in 13 patients (25.5%) and low BMD diagnosis preceding CeD in 9 patients (17.6%). Thirty-nine patients documented to be on gluten free diet. Iron-deficiency, vitamin D and calcium deficiency was treated in 8 (15.7%), 6 (11.8%) and 1 patient (2%) respectively. Fragility fractures were reported in 22 patients (43.1%) prior to diagnosis of low BMD, with 7(31.8%) vertebral, 7(31.8%) wrist, 4(18.2%) humeral and 2(3.9%) in neck of femur.

The mean-duration between initial and most recent DEXA scan was 5.5 years. T-scores between these scans were compared at different sites (Table 1). All 51 patients received treatment for osteoporosis/osteopenia as per prevalent guidelines. Oral bisphosphonates were prescribed in 43 patients (84.3%), denosumab in 26 patients (51%), zoledronic acid in 13 patients (25.5%), vitamin-D calcium in 4 patients (7.8%), romosozumab and teriparatide in one patient (1.96%) each. There were 3 vertebral and 1 wrist fracture suffered on Alendronic acid. There was no documentation on lifestyle and dietary advice for 10 patients (19.6%). Compliance with gluten free diet was noted in 5 patients (9.8%) during follow-up visits.

| Table 1. Comparison of T-Scores | | | | | | |
|---|---|---|---|---|---|---|
| | Initial DEXA (at diagnosis) | Standard Deviation | Latest DEXA (on osteoporosis treatment) | Standard Deviation | Rate of Change | Statistical significance ($p<0.05$) |
| **Lumbar Spine** | -2.89 | 1.20 | -2.24 | 1.23 | +6.89% | Significant |
| **Neck of Femur** | -2.26 | 0.82 | -1.93 | 0.76 | | Not significant |
| **Hip Total** | -1.82 | 1.19 | -1.80 | 0.74 | +3.05% | Not significant |

**Conclusion:**

The male-to-female ratio in our study (1:11.5) was higher than reported in literature (1.5:2). Baseline DEXA scan confirmed osteoporosis at lumbar-spine and osteopenia at neck of femur and hip. This would support the need for BMD measurement at diagnosis of CeD, in line with BSG guidelines 2014. CeD preferentially damages trabecular bone and our study show significant improvement in lumbar spine with treatment. Our analysis is from small number of patients. The compliance to GFD should be assessed at each clinic visit. All CeD patients should be treated for any calcium, vitamin D and other nutritional deficiencies.

### P62

### Implementing an osteoporosis shared decision-making intervention in Fracture Liaison Services: Interview findings from the iFraP process evaluation

Dr Laurna Bullock[1], Dr Andrea Cherrington[2], Professor Emma M Clark[3], Dr Jane Fleming[4,5], Ms Ida Bentley[6], Dr Elaine Nicholls[2,1], Mr David Webb[2], Ms Jo Smith[2], Ms Sarah Lewis[2], Professor Robert Horne[7], Professor Terence W O'Neill[8,9], Professor Christian D Mallen[1], Professor Clare Jinks[1], Professor Zoe Paskins[1,10]

[1]Centre for Musculoskeletal Health Research, School of Medicine, Keele University, Newcastle under Lyme, UK. [2]Keele Clinical Trials Unit, Keele University, Newcastle under Lyme, UK. [3]Bristol Medical School, Faculty of Health Sciences, University of Bristol, Bristol, UK. [4]Cambridge Public Health, University of Cambridge, Cambridge, UK. [5]Addenbrooke's Hospital Fracture Liaison Service, Cambridge University Hospitals NHS Trust, Cambridge, UK. [6]School of Medicine Research User Group, Keele University, Newcastle under Lyme, UK. [7]Centre for Behavioural Medicine, UCL School of Pharmacy, University College London, London, UK. [8]Centre for Epidemiology Versus Arthritis, University of Manchester, Manchester, UK. [9]NIHR Manchester Biomedical Research Centre, Manchester University NHS Foundation Trust, Manchester, UK. [10]Haywood Academic Rheumatology Centre, Midlands Partnership University NHS Foundation Trust, Stoke-on-Trent, UK

### Abstract

### Background

High quality shared decision-making (SDM) conversations involve people with or at risk of osteoporosis and clinicians working together to reach decisions about care. The **i**mproving uptake of **Fra**cture **P**revention drug treatments (iFraP) randomised controlled trial (RCT) tested a SDM intervention, comprising a computerised Decision Support Tool (DST), clinician training package, and information resources, in four UK Fracture Liaison Services (FLSs). The nested iFraP process evaluation aimed to explore perceived intervention acceptability, implementation, and the hypothesised mechanisms of impact and outcomes, including any contextual factors associated with variation.

### Methods

The iFraP process evaluation included semi-structured interviews with consenting (1) patients who received the iFraP intervention in their FLS appointment; (2) FLS clinicians who delivered the iFraP intervention; and (3) General Practitioners (GPs) who consulted with a patient following their FLS appointment.

Data were analysed using a framework approach, informed by the Normalisation Process Theory to unpick the 'work' required to implement the iFraP intervention.

### Results

Interviews were completed with 21 patients (90% female; mean age 66 years (range 54 – 82)), 8 FLS clinicians, and 2 GPs.

Overall, the intervention was viewed as acceptable. Many patients and clinicians wished for the intervention to be incorporated as part of usual care and some clinicians requested to use the intervention outside of the trial context.

All patients who had a face-to-face appointment reflected that the DST had been used by the clinician, and most had received the information resources. In contrast, patients who received a telephone appointment were less certain if the clinician had used the DST and some reported not receiving the information resources. Patients described engaging with the iFraP DST as requiring no extra work. In contrast, the work required for FLS clinicians to integrate the intervention in clinical practice varied, with some clinicians voicing challenges because of existing appointment lengths, IT skills, and adapting their well-established consultation 'flow'. Despite this, clinicians perceived the extra work as worthwhile because of perceived patient benefit.

The iFraP intervention was hypothesised to improve decision-making about osteoporosis medicines. In line with this, most patients and clinicians reflected that the intervention prompted patient involvement in discussions, elicited patient perceptions, and supported consistent, tailored and accessible information sharing.

**Conclusions**

The iFraP intervention was perceived as acceptable and implementable in FLS, with the potential to support SDM about osteoporosis medicines. Interview findings will be integrated with the RCT results to generate further insights about the iFraP intervention.

### P63

### Estimated number of adults missing osteoporosis treatment following a fragility fracture using the Fracture Liaison Service Database of England and Wales

Dr Laura Pugh[1], Ms Rumneet Ghumman[2], Professor Muhammad Javaid[3]

[1]Sherwood Forest Hospitals NHS FT, Sutton in Ashfield, UK. [2]Royal College of Physicians, London, UK. [3]University of Oxford, Oxford, UK

### Abstract

Introduction:

Despite clear national guidelines and government support for Fracture Liaison Services, the osteoporosis treatment gap remains significant. The Fracture Liaison Service Database (FLS-DB), a national audit run by the Royal College of Physicians (RCP), has recently expanded its reporting to highlight this issue.

Method:

Previously the FLS-DB benchmarked data from those trusts submitting data to the audit. From January 2025, an extra column has been added to show 'Missed Opportunities' that includes data from sites not participating in the FLS-DB. Using local hip fracture data for 2022 from the National Hip Fracture Database (NHFD) figures, the predicted local FLS caseload was determined by multiplying the number of hip fractures by 5. Expecting 80% of the predicted caseload to be identified, at least 50% of those to be recommended treatment (accounting for mortality, severe comorbidities etc.) and 80% of those initiating and staying on treatment up to 12 months gives the expected on treatment population. This was compared with the data from the FLS-DB and NHFD KPI set to generate the number with a missed opportunity.

Results:

77 FLS are participating with the FLS-DB with 82 NHFD sites not covered by an FLS. While 80,767 records were submitted in 2022, the missed opportunity count was estimated to be 56,550 patients (48,214 in England and 6,180 in Wales) per annum. When the missed opportunity estimate was analysed in 36 ICSs, there was an over 10 fold difference in the estimate.

Conclusions:

Despite clear guidelines and prioritisation of FLSs, over 50,000 patients are not on osteoporosis treatment when they should be. By making this data visible at the local hospital and ICS / Health board level, care providers can better judge the level of resources required for FLS locally, and the data provides support for ICSs in FLS implementation.

**P64**

### Role of primary care in reducing the health impact of osteoporosis in Highland; reflections on existing system and future opportunities

Dr Nicholas Cemm[1], Dr Stephen Bridgman[1,2,3,4]

[1]NHS Highland, Inverness, UK. [2]Public Health Scotland, Edinburgh, UK. [3]Argyll and Bute Health and Social Care Partnership, Edinburgh, UK. [4]University of Aberdeen, Aberdeen, UK

### Abstract

Background:

Osteoporosis and its associated fragility fractures account for substantial morbidity, disability and mortality to individuals suffering from it, as well as placing a sizeable burden on the health and other systems. This disability and service burden in NHS Highland is predicted to increase substantially as our population ages. Primary care have a potentially key role in Highland in managing and preventing the disease burden. This study examined the role, barriers, and opportunities to improve population bone health through primary care.

Methods:

Best practice management for bone health from a primary care perspective was summarised using; the Scottish Intercollegiate Guideline Network (SIGN142) guideline on the management of osteoporosis and prevention of fragility fractures; National Institute of Clinical Excellence (NICE) guideline on osteoporosis; National Osteoporosis Guideline Group (NOGG) clinical guideline. Secondly, suggested best practice management was contextualised within the current NHS Highland system, by reviewing local guidelines and protocols informed by discussions with relevant professionals with involvement in the management of bone health locally

Results:

In NHS Highland there are helpful local guidelines on the management of osteoporosis available to GPs. However, different guidelines from different expert bodies create some difficulties and challenges. Most general practices in Highland use the Vision clinical system, and while Vision used to include the QFracture automatic calculator, GPs now have to manually assess fracture risk which takes much longer. Scotland has also withdrawn the Quality Outcomes Framework (QOF) which enabled a general practice to be funded to have a register of patients with osteoporosis. Currently there is no incentive system in Scotland for GPs wishing to focus on bone health, and, given workload, most preventative opportunities are not able to be taken up.

Discussion:

GPs, along with the wider multi-disciplinary team (MDT), such as primary care pharmacists, are well placed to follow up on patients after treatment is started and for patients to be able to discuss any concerns, being directly accessible to patients unlike the majority of secondary care. While primary care is well placed to undertake preventative work in bone health, without the primary care system becoming dramatically less stretched from dealing with a large amount of reactive work, it is unrealistic to expect GPs to be able to undertake on additional preventative work without any system changes or support. Opportunities for improvement in Scotland will be discussed.

**P65**

Withdrawn

**P66**

### The United Kingdom Adult NorthStar Network Consensus Recommendations for the Management of Osteoporosis in Adults with Duchenne Muscular Dystrophy and Transition to Adult Care

Dr Shima Abdelrahman[1], Dr Sarah McCarrison[1,2], Prof David Armstrong[3], Dr Terry Aspray[4], Dr Judith Bubbear[5], Ms Fleur Chandler[6], Prof Emma Clark[7], Dr Nicola Crabtree[8], Prof Emma Duncan[9], Prof Neil Gittoes[10], Ms Alex Johnson[6], Dr Gavin Langlands[11], Dr James Lilleker[12], Prof Raja Padidela[13], Prof Peter Selby[14], Dr Vrinda Saraff[15], Dr Maria Talla[16], Dr Clare Thornton[17], Dr Claire Wood[18], Prof Jennifer Walsh[19], Prod Tracey Willis[20], Prof Kassim Javaid[21], Prof Richard Keen[22], Prof Rosaline Quinlivan[23], Dr Sze Choong Wong[1,2]

[1]University of Glasgow, Glasgow, UK. [2]Department of Paediatric Endocrinology, Royal Hospital for Children, Glasgow, UK. [3]Western Health and Social Care Trust (NI) and Nutrition Innovation Centre for Food and Health, Ulster University, Londonderry, UK. [4]NIHR Newcastle Biomedical Research Centre, Translational Clinical Research Institute, Newcastle University and Newcastle-upon-Tyne Hospitals NHS Trust, Newcastle upon Tyne, UK. [5]Centre for Metabolic Bone Disease, Royal National Orthopaedic Hospital NHS Trust, London, UK. [6]Duchenne UK, Hammersmith, UK. [7]Bristol Medical School, University of Bristol, Bristol, UK. [8]Birmingham Women's and Children's NHS Foundation Trust, Birmingham, UK. [9]Department of Twin Research and Genetic Epidemiology, School of Life Course & Population Sciences, Faculty of Life Sciences and Medicine, King's College London, and Dept of endocrinology, Guy's and St Thomas' NHS foundation trust, London, UK. [10]Centre for Endocrinology, Diabetes and Metabolism, Queen Elizabeth Hospital, University Hospitals Birmingham & University of Birmingham, Birmingham, UK. [11]Institute of Neurological Sciences, NHS Greater Glasgow and Clyde, Glasgow, UK. [12]Manchester Centre for Clinical Neuroscience, Manchester Academic Health Science Centre, Salford Royal Hospital, Northern Care Alliance NHS Foundation Trust, Salford, UK. [13]Department of Paediatric Endocrinology, Royal Manchester Children's Hospital and Faculty of Biology, Medicine & Health, University of Manchester, Manchester, UK. [14]Department of Diabetes, Endocrinology and Metabolism, Manchester Royal Infirmary, Manchester University NHS FT, Manchester, UK. [15]Department of Paediatric Endocrinology, Birmingham Women's and Children's Hospital, Birmingham, UK. [16]Medical Unit, Royal Alexandra Hospital, Paisley, UK. [17]Department of Rheumatology, University College London Hospital, London, UK. [18]Newcastle University Translational and Clinical Research Institute and Paediatric Endocrinology department at Great North Children's Hospital, Newcastle upon Tyne, UK. [19]Mellanby Centre for Bone Research, University of Sheffield, Sheffield, UK. [20]Robert Jones and Agnes Hunt Orthopaedic Hospital, NHS Foundation Trust, Oswestry, UK. [21]Nuffield Department of Orthopaedics, Rheumatology and Musculoskeletal Science (NDORMS), University of Oxford, Oxford, UK. [22]Metabolic Bone Disease Unit, Royal National Orthopaedic Hospital, Stanmore, UK. [23]Centre for Neuromuscular Diseases, National Hospital, UCL Queen Square Institute of Neurology, London, UK

### Abstract

**Objectives:** Osteoporosis is a common complication in Duchenne muscular dystrophy (DMD). Current clinical guidelines focus primarily on paediatric care, with limited guidance for managing osteoporosis during the transition to adulthood and long-term management of osteoporosis therapy initiated in paediatric care.

**Methods:** In 2023, a UK expert working group was formed with the task of developing national expert consensus on the management of osteoporosis in adults with DMD in the adult NorthStar Network. The working group included 13 adult and 5 paediatric bone specialists, 3 adult neuromuscular clinicians (2 with paediatric experience), a clinician-scientist/densitometrist, and 3 patient representatives. Systematic and scoping reviews of osteoporosis therapies for DMD, glucocorticoid-induced osteoporosis guidelines for adults, and DXA-based fracture predictions in DMD were conducted. A survey was distributed via patient organisations to paediatric clinicians managing DMD-related osteoporosis, with focus on care during transition. Feedback on bone health management was also gathered through a focus group of adults with DMD. Four online meetings of the expert working group were held. Using a modified Delphi approach, consensus clinical recommendations were developed.

**Results:** Consensus recommendations were established through three Delphi voting rounds, with 80% agreement required. Thirteen clinical guidance statements were developed. Key recommendations include:

- Adults with DMD should undergo osteoporosis and fracture risk assessments, including vertebral fracture (VF) assessment and DXA bone density, if expected to influence clinical decision making.
- VF reassessments are recommended every two years for those on glucocorticoids and should be individualised for those who are not on glucocorticoid treatment and/or, those with metal instrumentation for scoliosis.
- For young people on long-term bisphosphonate therapy (>10 years) initiated in childhood and who have completed puberty, discontinuation of therapy should be considered at transition, depending on clinical risk factors. The clinical risk factors include glucocorticoid therapy, presence of VF at most recent evaluation, new VF and/or worsening VF during treatment with osteoporosis therapy or recent low trauma long bone fracture.
- Upon discontinuation of osteoporosis therapy, re-evaluation of the need to re-initiate osteoporosis treatment is recommended if low trauma fractures are sustained off treatment or if there is significant bone density decline, or at two years post-discontinuation.

**Conclusion:** Using a Delphi-based systematic process, this UK expert working group developed national consensus guidance for managing osteoporosis in adults with DMD. These recommendations address critical gaps in care during the transition from paediatric to adult services, ensuring a comprehensive and individualised approach to bone health management.

### P67

### Gaining consensus on recommendations to improve the understanding of bone density scans and results: A nominal group technique

Miss chelsea Kettle[1,2], Dr Fay Manning[3], Mrs Beverly Henderson[4], Mrs Jill Griffin[5], Professor Karen Knapp[3], Dr jo Butterworth[6], Professor Clare Jinks[1], Dr Laurna Bullock[1], Professor Zoe Paskins[1,2]

[1]Centre for Musculoskeletal Health Research, School of Medicine, Keele University, Staffordshire, UK. [2]Haywood Academic Rheumatology Centre, Haywood Hospital, Midlands Partnership University NHS Foundation Trust, Staffordshire, UK. [3]Health and Care Professions, Faculty of Health and Life Sciences, University of Exeter, Exeter, UK. [4]Keele University Research User Group, Keele University, Staffordshire, UK. [5]Royal Osteoporosis Society, Bath, UK. [6]Exeter Collaboration for Academic Primary Care (APEx), University of Exeter Medical School, Exeter, UK

### Abstract

### Background

Patients and clinicians report difficulty understanding dual-energy X-ray absorptiometry (DXA) scans and results, impacting clinical and shared decision-making about osteoporosis medicines. The INDEX (understandINg bone DEnsity [dXa] scans) study aimed to develop recommendations to enhance understanding and communication of DXA scans and results.

### Methods

We employed a modified hybrid in-person and online Nominal Group Technique (NGT). A convenience sample of patients, DXA service providers (e.g. DXA technicians, radiologists), and DXA service referrers (e.g. GPs, physiotherapists) were invited.

Initially, twenty-one candidate recommendations were drafted by the study team, informed by interviews exploring patients' and clinicians' unmet DXA information needs, and public contributor and stakeholder discussions. Recommendations were organised according to audience; for (1) DXA providers or (2) DXA referrers. Participants reviewed and gave feedback on candidate recommendations remotely before the first NGT meeting.

At the first NGT meeting, participants refined candidate recommendations and generated new recommendations through discussion. Afterwards, participants individually rated their agreement with each recommendation remotely. Ratings were discussed at the second NGT meeting followed by individual rerating considering discussions. Each recommendation was rated on a 7-point Likert scale (from 1 'strongly disagree' to 7 'strongly agree'). For analysis, consensus was pre-defined as >70% agreement (excluding 'no opinion' responses), and recommendations were ranked by scored importance.

### Findings

The first NGT meeting included four patients and 13 clinicians from various disciplines (e.g. GPs, DXA service leads). Ten additional recommendations were developed through discussion. In the first rating exercise, 30 of

31 recommendations achieved >70% agreement (55%-100%). These results were discussed in the second NGT meeting with two patients and 14 clinicians, where 30 recommendations again achieved >70% agreement.

For DXA providers, the top recommendation was to ‘include a clinical interpretation in reports’, with other agreed recommendations addressing DXA report formatting, using lay language, and acknowledging the limitations of DXA scan results (e.g. for people from minority ethnic backgrounds). For DXA referrers, the top ranked recommendation was: ‘when communicating [DXA] results, emphasize that bone mineral density is only part of a person’s fracture risk and it needs to be considered in context of other fracture risk factors’. Other recommendations reaching agreement considered issues such as offering patients the opportunity to discuss DXA results, checking patient understanding, and training to understand and communicate scan results.

**Conclusions**

The INDEX recommendations will inform Royal Osteoporosis Society guidelines and resource development, with the aim of supporting clinical and shared decision-making and osteoporosis medicine uptake.

**P68**

### A preliminary study on hair and nails using Raman spectroscopy and its potential for assessing bone quality

Dr Nai-Hao Yin[1], Professor Frances Griffiths[2], Professor Helen Dawes[3], Dr Richard van Arkel[4], Dr Claire Mann[2], Dr Marwan Bukhari[5], Dr Jemma Kerns[1]

[1]Lancaster University, Lancaster, UK. [2]The University of Warwick, Warwick, UK. [3]University of Exeter, Exeter, UK. [4]Imperial College London, London, UK. [5]University Hospitals of Morecambe Bay NHS Trust, Lancaster, UK

**Abstract**

### Introduction

The structural protein keratin in hair and nails can show parallel changes to the collagen in bones. Studies have shown that the composition of hair and nails, both easily accessible tissues, can show changes associated with bone quality. Raman spectroscopy is a light-based technique and sensitive to molecular level changes and has previously been applied to hair and nails separately to identify spectral differences between healthy and osteoporotic patients. This study aims is to test the feasibility of spectral measurements from both hair and nail samples of the same individual to identify age-related changes.

### Methods

Ten female participants (Group A: 55–62 yrs, Group B: 79–87 yrs; n=5 for each), without a diagnosis of osteoporosis or history of fragility fractures, donated nail clippings and a strand of hair when upon referral to the rheumatology clinic for bone density scans. Hair samples were untreated while nail clippings were cleaned with an acetone-based solution and left to dry prior to Raman spectroscopy measurements (785nm, 300mW at source), using standardised protocols. Spectral data (450-1800cm$^{-1}$) were baseline corrected with a polynomial subtraction and normalised to phenylalanine (1002cm$^{-1}$) prior to statistical analyses for group comparisons.

### Results

In total 30 hair and 50 nail spectra were collected and analysed. In general, the nails have higher spectral quality than hair samples. Besides the peaks at 831cm$^{-1}$ and 851cm$^{-1}$, the hair spectra had higher overall Raman intensity than the nail (Fig. 1A). Independent t-tests showed significant age differences among nail samples in amide I at 1652cm$^{-1}$ (Group A: 1.42±0.14 vs. Group B: 1.29±0.23, $p$=0.027), $CH_2$ bending at 1450cm$^{-1}$ (1.27±0.09 vs. 1.18±0.13, $p$=0.007), amide III at 1316cm$^{-1}$ (0.74±0.06 vs. 0.68±0.09, $p$=0.007), and C-C stretching band at 935cm$^{-1}$ (0.73±0.08 vs. 0.64±0.11, $p$=0.003) while these peaks were not significantly different in hair samples. There were no significant group differences at 510cm$^{-1}$ for both hair (0.81±0.08 vs. 0.87±0.11, $p$=0.099) and nail samples (0.43±0.10 vs. 0.48±0.07, $p$=0.069). Differentiation due to age was apparent for both sample types when analysed using principal component analysis with linear discriminant analysis (Fig. 1B-C).

Discussion

This study demonstrated that Raman spectroscopy can identify age-related differences in both hair and nail samples using univariate and multivariate analyses. A larger dataset will be analysed to assess whether this combined tissue approach correlates with bone mineral density.

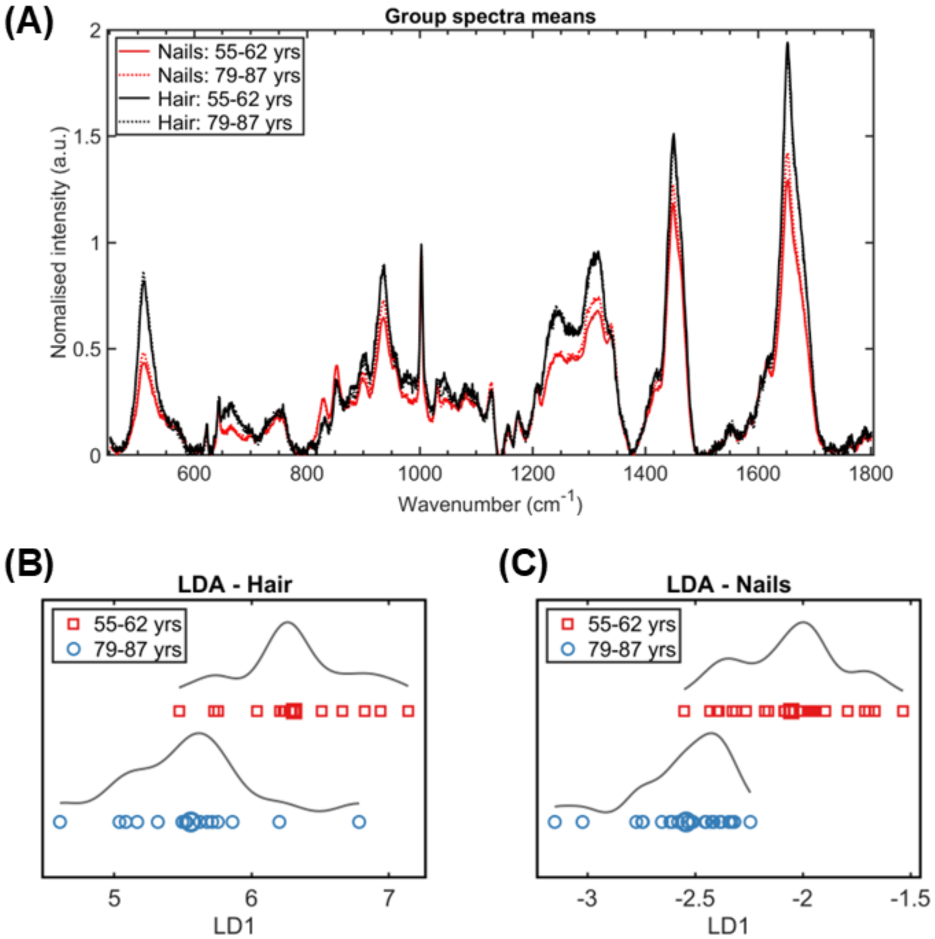

**P69**

Withdrawn

**P70**

Withdrawn

**P71**

Withdrawn

### P72

### ATDC5 chondrocyte micromass mineralising cultures as a novel in vitro model for investigating ochronosis of alkaptonuria

Miss Daisy Quinn[1], Dr Leah Wells[2], Mr Mark Hopkinson[2], Professor George Bou-Gharios[1], Professor James Gallagher[1], Professor Andrew Pitsillides[2], Dr Scott Roberts[2], Dr Juliette Hughes[1]

[1]University of Liverpool, Liverpool, UK. [2]Royal Veterinary College, London, UK

### Abstract

Alkaptonuria (AKU) is an ultra-rare disease characterized by rapid and progressive tissue deterioration and development of severe and early onset arthropathies. Defects in the liver *HGD* gene results in an enzyme deficiency that prevents the breakdown of homogentisic acid (HGA), leading to excessive circulating levels from birth. The multisystemic manifestations of AKU are the result of HGA accumulation which permits the formation of 'ochronotic pigment' that deposits in collagen rich tissues, most notably cartilage, causing degeneration.

Study of AKU mice and human AKU tissues has shown that the primary site of ochronotic pigmentation is within articular calcified cartilage (ACC), before spreading superficially, and to other connective tissues. To better understand the early pathological changes that occur in AKU, we have developed a novel in vitro model of ochronosis using ATDC5 mouse chondroprogenitor cells cultured as 3D mineralised micromasses to recapitulate the ACC.

Micromasses were cultured in HGA-supplemented media at concentrations ranging from 3-330 µM, with an initial chondrogenic differentiation phase followed by a mineralisation phase over a total of 12 days. The effects of HGA on calcium deposition and proteoglycan levels in the extracellular matrix (ECM) were assessed at day 12 using alizarin red and alcian blue staining, respectively (Figure.1). HGA-derived pigment deposition was evaluated pre-fixation using fluorescent imaging due to HGA's auto fluorescent properties at day 12 (Figure 1).

Quantification of alizarin red staining showed that HGA promoted calcification up to a concentration of 10 µM, by approximately 30%. Additionally, elevated proteoglycan levels were observed when HGA was supplemented into the media up to 33 µM, indicating shifts in the ECM composition. For the first time, we used fluorescence imaging to localise HGA-derived pigment in mineralising micromass cultures, and discovered that at day 12, pigment associated with regions of mineral deposition, suggesting an affinity to calcifying cartilage.

Our data show that excess HGA affects both hypertrophic chondrocytes and the surrounding ECM in the initial stages of disease progression in AKU. This study shows that this novel ATDC5 model has utility in unravelling the mechanisms underlying the early pathological changes in AKU calcified cartilage.

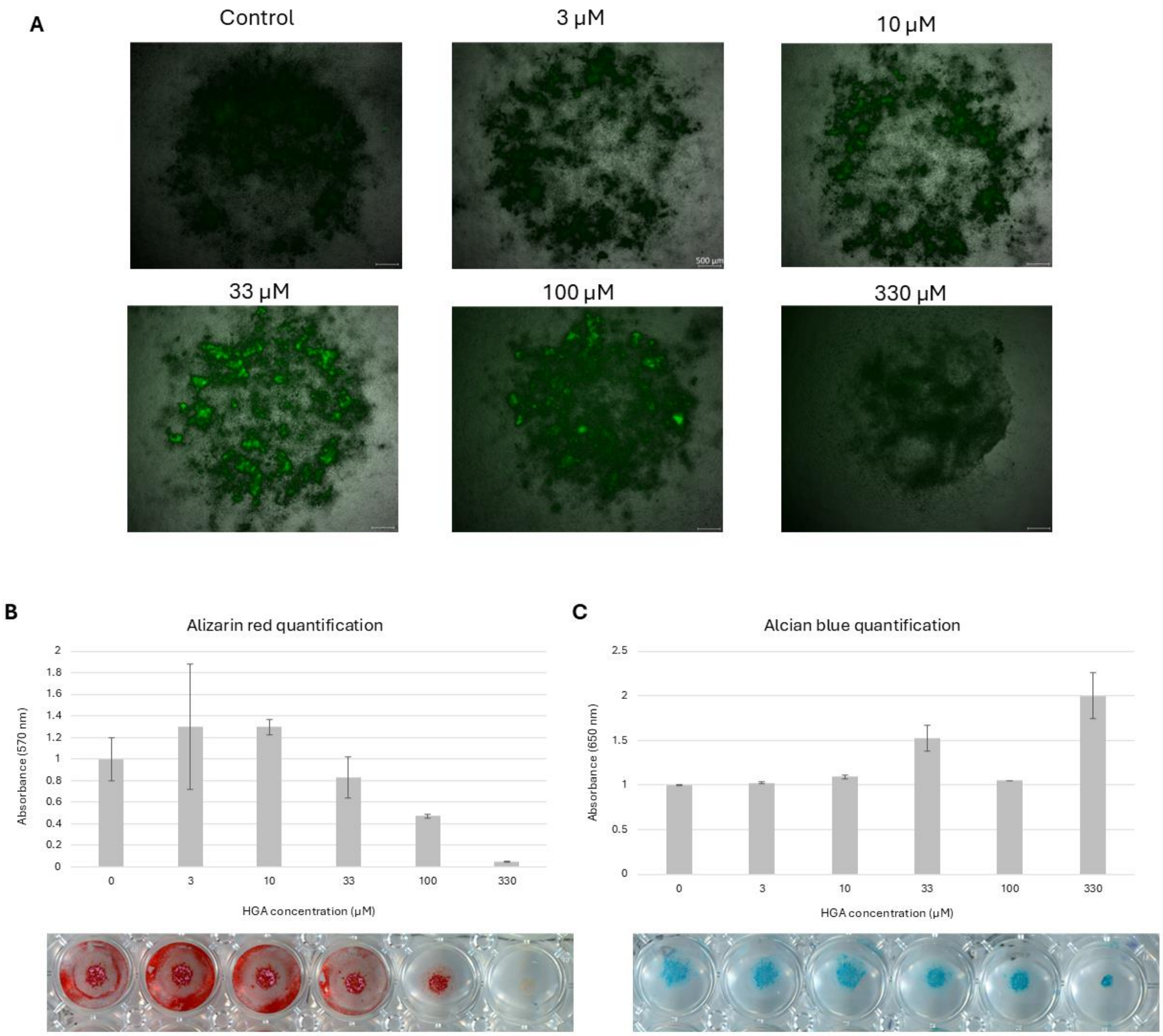

**Figure 1**. (A) Layered brightfield and 488 nm green channel images of auto-fluorescent HGA localised to mineral deposits at 3-330 µM HGA at day 12 (Scale bar = 500 µm). Quantification of (B) alizarin red staining and (C) alcian blue staining with representative images of stained masses aligned with corresponding HGA concentrations and optical density measurements.

**P73**

Withdrawn

**P74**

Withdrawn

**P75**

Withdrawn

### P76

### Impact of a Low Threshold for Serum Alkaline Phosphatase on Hypophosphatasia Case Finding

Dr Zahra M. Iftikhar[1], Dr Najla Elndari[1], Dr Onaopemipo Akingbade[1], Dr Gavin Mercer-Smith[2], Dr Marta Bertoli[1], Dr Nishanthi Thalayasingham[1], Dr Terry Aspray[1]

[1]Newcastle upon Tyne Hospitals NHS Foundation Trust, Newcastle, UK. [2]Northumbria Healthcare NHS Foundation Trust, Newcastle, UK

### Abstract

**Background**
As our hospital laboratory intended to introduce routine reporting of low serum alkaline phosphatase (ALP) levels (less than 30 U/L), we anticipated a need to support clinical colleagues in the interpretation and management of newly identified cases of low serum alkaline phosphatase and possible adult phenotype hypophosphatasia.

**Purpose**
This service evaluation aimed to assess the impact of change in laboratory reporting on clinical practice and case identification.

**Methods**
We reviewed all laboratory measurements of ALP for one year (2019) to scope the project and developed a clinical protocol to guide clinicians in responding to flagged results, with recommendations for further investigations and advice from the Bone clinics via NHS "Advice and Guidance". We compared results from before (2019) and after (January-February 2024) low-ALP reporting, and then after the distribution of the clinical protocol in March 2024 (July-August, 2024). Chi-squared tests for independence are reported.

**Results**
Retrospective review of laboratory measurements of ALP from 2019 found 390 cases with an ALP less than 30 U/L, from which a convenience sample of 100 cases identified 10 (10%) with a persistently low ALP (PL-ALP).

In January-February 2024, of 47 cases with low ALP, 14 (30%) had PL-ALP, with 6 (43%) being referred to the Bone Clinic. All cases had a raised PLP/PA ratio; 3 (50%) had genetic testing, and 2 had a recognized genotype (1 result awaited).

In July–August 2024, of 32 cases with low ALP, 16 (50%) had PL-ALP, and 2 (13%) were referred to the Bone Clinic. Both had elevated PLP/PA levels and subsequently underwent genetic testing, with 1 confirmed genotype (1 result awaited). In July-August, more cases had ALP repeated (31% vs. 6% ($X^2(1,n=79)$, $p<.01$)), as were tests for other causes of low ALP ($X^2(1,n=79)$, $p<0.05$), but there was a non-significant decrease in hospital referrals for PL-ALP from 43% vs.13% ($X^2(1,n=30)=3.5187$, $p=0.06$).

**Conclusions**
We have developed a protocol for low ALP, so that PL-ALP can be identified and appropriately investigated in primary care, with the support of a specialist clinic. Patients with adult phenotype hypophosphatasia have been identified with lifestyle and therapeutic advice and, in some cases, genetic counselling given.

### P77

### ACDC as a rare cause of premature chondrocalcinosis

Professor Muhammad Javaid[1], Dr Karen Partington[2]

[1]University of Oxford, Oxford, UK. [2]Oxford University Hospitals NHS Foundation Trust, Oxford, UK

### Abstract

A 37 Pakistani woman, with parents who are first cousins, was referred to the metabolic bone clinic with inflammatory chondrocalcinosis from the age of 13 years. There was a history of migratory joint swelling of the knees, elbows, DIP and PIP joints from 3 times a month to once every 6 months. There was a variable response to 15 mg of prednisolone and attacks were usually managed with naproxen. The patient also had painless oral ulcers occasionally and non-scaring hair loss.

In her family history, 2 paternal aunts had similar arthritis but also severe arterial leg ulceration requiring amputation at the age of 50 years.

The patient was normal height with no synovitis. There was reduced wrist extension, hip internal rotation with a normal range of movement of the spine.

Laboratory tests demonstrated normal bone, phosphate, PTH, 25OH vitamin D with negative CCP/ RF.

An x-ray of the pelvis demonstrated ectopic calcification around the hip joint but also extensive arterial calcification (Figure).

Whole genome sequencing demonstrated a homozygous likely pathogenic variant in *NT5E* that is associated with Arterial calcification due to deficiency of CD73 (ACDC). The mechanism by which ACDC causes vascular calcification is unclear and may involve both low adenosine and low pyrophosphate. Typically ACDC is a slowly progressive disease with multiple collaterals resulting in no amputations in the NIH cohort. Etidronate for 14 days every 3 months over 3 years may slow progression in early disease.

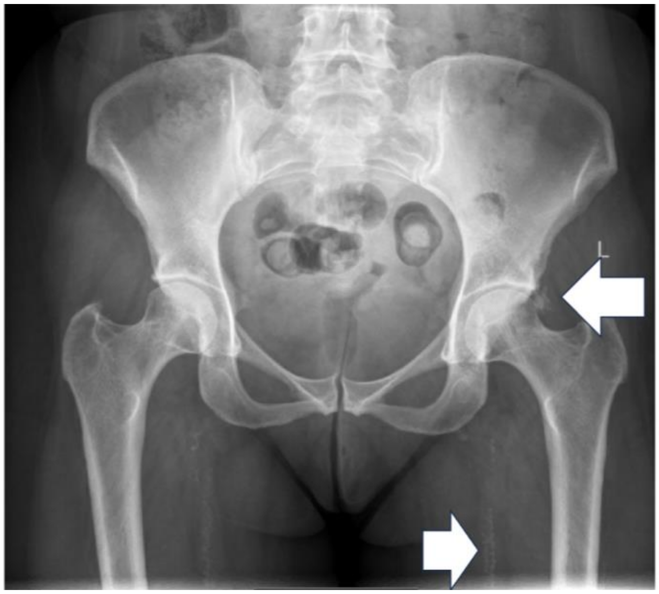

### P78

### The IMPACT Survey in the UK: genetic testing and diagnostic experiences of people with osteogenesis imperfecta

PhD Samantha Prince[1,2], Ms Ingunn Westerheim[3], Mr Taco van Welzenis[3], Ms Tracy Hart[4], MD Oliver Semler[5], PhD Frank Rauch[6], MD Cathleen Raggio[7], MSc Melanie Anderson[1], MD, PhD Lena Lande Wekre[8]

[1]Wickenstones Ltd, Oxford, UK. [2]Mereo BioPharma, London, UK. [3]Osteogenesis Imperfecta Federation Europe, Heffen, Belgium. [4]Osteogenesis Imperfecta Foundation, Gaithersburg, USA. [5]University of Cologne, Cologne, Germany. [6]McGill University, Montreal, Canada. [7]Hospital for Special Surgery, New York, USA. [8]Sunnaas Rehabilitation Hospital, Sunnaas, Norway

### Abstract

The UK healthcare journey of people with osteogenesis imperfecta (OI)—a rare hereditary connective tissue disorder—is not completely understood. This analysis aims to provide insights on patterns of genetic testing, and diagnostic experiences of individuals with OI.

The IMPACT Survey was developed by the OI Federation Europe, the OI Foundation and an international steering committee of experts to explore the clinical, humanistic and economic impact of OI. The survey was fielded online from July–September 2021 and open to adults (aged ≥18 years) and adolescents (aged 12–17 years) with OI, caregivers (CGs; with or without OI) and other close relatives. This analysis presents findings from adults with OI who responded to the survey themselves and results from CGs who responded on behalf of their care recipients: children (aged 0–17 years). Data were analysed using Microsoft Excel.

Among UK respondents (n=185), 40 were CGs providing proxy responses for children with OI (n=44) and 144 were adults with OI. Sex distribution of children was largely balanced (45.5% female) with a mean age of 6.4 years (standard deviation [SD] 4.5). Most adults (73.6%) were female with a mean age of 46.9 years (SD 14.8). Self-reported OI severity was found to be similar in children and adults (children: mild 45.5%, moderate 29.5% and severe 15.9%; adults: mild 41.0%, moderate 42.4% and severe 11.1%). Type I OI was the most reported in both children and adults (45.5% and 47.2%, respectively). Genetic confirmation of OI status was received by 81.8% of children and 71.5% of adults. Not being offered a test was the primary reason given for an unconfirmed genetic diagnosis. Mean age at diagnosis was 1.4 years (SD 2.2) for children and 4.5 years (SD 10.8) for adults; diagnosis had not been received by 11.8% of adults until after the age of 11 years. When experiences were queried, 1 child (3.2%) and 11 adults (7.6%) reportedly had initially been misdiagnosed with a different condition. Misdiagnoses included achondroplasia (n=1 child), hypermobility (n=3 adults) and coordination disorder (n=2 adults). Notably, 25–26% of children and adults indicated that their OI had initially been suspected non-accidental injury.

Data indicate that genetic testing for OI in the UK is commonly offered but highlight ongoing challenges around diagnosis for individuals with OI and their families. Increased disease-specific awareness is crucial for supporting both the clinical, and patient community in the diagnostic journey.

**LB01**

### Ultrasonic vs. Conventional Saw Bone Cutting in a Rat Tibial Fracture Model: Comparable Healing Outcomes

Dr. Nisreen Al-Namnam[1], Ms Steph Collishaw[1], Ms Melanie Wheeldon[1], Dr. Xuan Li[2,3], Prof. Margaret Lucas[3], Prof. A. Hamish R. W Simpson[1,4]

[1]University of Edinburgh, Edinburgh, UK. [2]University of Southampton, Southampton, UK. [3]University of Glasgow, Glasgow, UK. [4]Royal Infirmary of Edinburgh, Edinburgh, UK

### Abstract

INTRODUCTION

Ex vivo studies have shown that Ultrasonic bone scalpels cut bone with greater precision.  However, the effect of ultrasonic cutting of bone healing has not been evaluated. This study compared an innovative miniaturised ultrasonic bone scalpel and Conventional electrical saw in a rat tibial osteotomy model. The ultrasonic device employed a flextensional configuration with a pre-stressed piezoelectric (PZT) stack and a single metal frame, amplifying blade movement for greater cutting efficiency.

METHODS

Eight adult male Sprague-Dawley rats were divided into two groups: Conventional electrical saw (NS) and Ultrasonic (US) cutting. Anaesthesia and analgesics were given, and tibial osteotomies were created using either NS or US bone scalpel (25 kHz at 60 µm). Continuous saline cooling was applied, and intramedullary fixation was done with an 18G needle. X-rays were taken at multiple time points. All procedures were performed under a UK Home Office License. Bone healing was also assessed using micro-CT imaging and biomechanical four-point bending tests. Additionally, healing was assessed histologically and with fluorochrome bone labelling.

RESULTS and DISCUSSION

At 35 days post-osteotomy, bone mineral density (BMD) and stiffness were compared between the ultrasonic and Conventional electrical saw groups, with contralateral (undivided) limbs served as controls. No significant stiffness and BMD (NS: 0.93 ± 0.08; US: 0.98 ± 0.12) difference was observed between groups, although the US group (76.89 ± 25.50 N/mm) exhibited slightly lower stiffness than the NS group (95.66 ± 14.06 N/mm).Callus index and RUST scores was progressed at a nearly identical rate in both groups, indicating comparable bone healing. These findings suggest that US bone cutting does not negatively impact fracture healing compared to traditional saw methods (Fig 1).

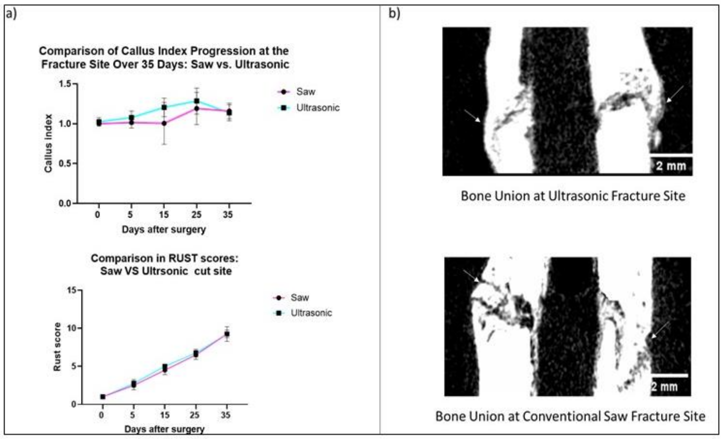

**Figure 1: Progression of the callus index and Rust score (a) and Micro-CT image (b) of the fracture site (white arrows) over 35 days at the Normal saw and ultrasonic cut sites displayed comparable results. Data are presented as mean ± SEM.**

Bone formation was visualised using time-specific fluorochromes: tetracycline (day 5, yellow), calcein (day 15, green), and alizarin (day 25, red), outlining the mineralisation timeline. Double-line labelling indicated progressive deposition between days 15 and 25. Both osteotomy techniques supported effective bone regeneration, but the Saw group displayed more advanced remodelling by day 35, with greater presence of osteonal structures (Fig 2a) and collagen maturation, indicating a higher degree of structural maturation. Both groups exhibited similar labelling patterns, with comparable area and perimeter measurements of newly deposited bone (Fig 2b).

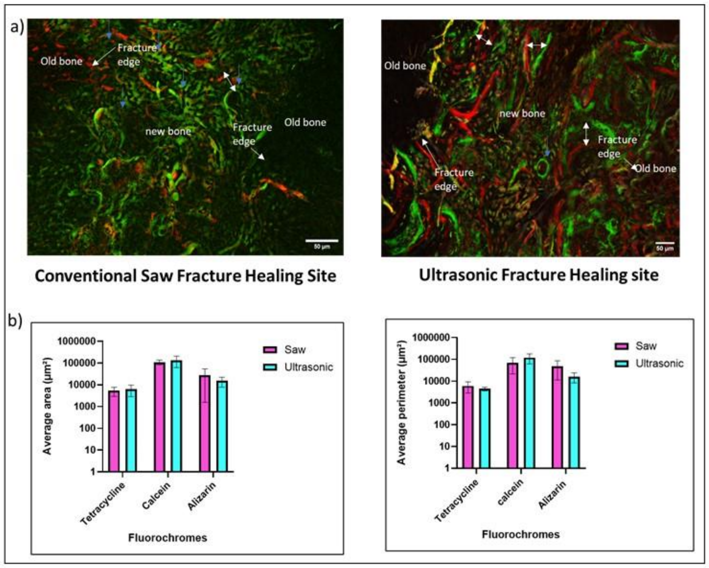

**Figure 4. Fluorescence photomicrograph of the fracture site and comparison of fluorochrome measurements in newly deposited bone. a) Triple fluorochrome labelling (tetracycline, calcein, alizarin) revealed sequential bone formation at ultrasonic fracture sites. Multicoloured osteons indicated active remodelling, with distinct deposition phases shown by separated fluorochrome lines. b) Fluorochrome-labelled bone area and perimeter were measured at 5, 15, and 25 days post-surgery, comparing ultrasonic and normal saw cuts. Data are presented as mean ± SEM from 4 rats per group, each with a healing tibial fracture.**

CONCLUSION

Ultrasonic cutting has demonstrated higher precision in ex vivo bone samples, but it was not known whether it was deleterious to bone healing. This study has shown that ultrasonic cutting does not impair fracture repair.

**LB02**

### Cannabinoid Treatment Induces Cell Death in Multiple Myeloma, Prostate and Pancreatic Cancer Cells and Enhances Impact of Chemotherapeutic Agents

Dr Charlotte Palmer[1], Dr Zeynep Kaya[1], Prof Claire Edwards[1], Dr Valentino Parravicini[2], Prof James Edwards[1]

[1]University of Oxford, Oxford, UK. [2]Octavian, Oxford, UK

### Abstract

For centuries, cannabis has been used as a medicine including for sore eyes and haemorrhoids by ancient Egyptians, and more recently in disorders such as neurological seizures and inflammation. Despite clear beneficial potential, research into medicinal cannabis has been vastly under-funded. This is particularly true in cancer sufferers where cannabis use might occur alongside treatment as an analgesic and antiemetic, easing pain and chemotherapy-induced sickness. In such patients, indirect beneficial effects on tumour development have been observed. Cannabis consists of multiple bioactive proteins (>130 endocannabinoids). We hypothesized that exposure to cannabinoids impacts tumour cell growth directly and improves chemotherapeutic action.

In this study, we tested a cannabinoid panel consisting of cannabidiol (CBD), cannabigerol (CBG), cannabinol (CBN) and novel CBD-, CBG-derivatives in normal and malignant prostate- and pancreatic cancer cells, and multiple myeloma. Cell growth, death and cell migration were assayed using Live cell imaging.

CBD, CBG, and CBN increased cell death in normal (PNT2) and cancerous (ArCapM) prostate cells (10-100uM, 24hrs), whilst novel agents OCT-1, -21 preferentially triggered cell death in malignant cells (by up to 40x fold in ArCapM, x 300fold in PC3, compared to non-tumour). Similarly, OCT-23 showed over x200 fold cell death induction, demonstrating significant preference for tumour cells. CBN reduced ArCapM cell migration (10mM, 36h by x40) and comparable to chemotherapeutic Docetaxel (DTX, 5.4nM). Interestingly, pre-treatment of PC3 with CBD, CBG, CBN prior to DTX, led to a significantly greater reduction in prostate cancer cell growth than DTX alone. This effect was seen using several novel CBD-, CBG-derivatives also, but interestingly, not with the naturally occurring endogenous endocannabinoid-like analogues oleoylethanolamide (OEA) or palmitoylethanolamide (PEA), which do not bind cannabinoid receptors. These data suggest this effect is mediated via direct canonical cannabinoid receptor binding.

Also, treatment of human myeloma cells (JJN3) vs normal monocytes (THP1) with CBD, CBG, CBN (1mM) showed up to 50-fold increase in cell death in tumour Vs non-tumour cells, and OCT-23 treatment preferentially killed myeloma cells at doses as low as 1nM ($p<0.001$). Importantly, CBD induced double myeloma cell death compared to established chemotherapeutic DTX. A similar increase in apoptosis was seen in pancreatic ductal adenocarcinoma cells (PANC-1, Vs DMSO vehicle), and with novel compounds OCT-1, -2, and -3 (1mM). This study indicates that principal cannabinoids (CBD, CBG, CBN) and new derivatives have potential to reduce tumour burden across multiple cancers and may prove beneficial as an adjuvant to established chemotherapeutic approaches.

### LB03

### Patient reported outcomes (PROs) from a real-world study of burosumab treatment in adults with X-linked hypophosphataemia (XLH) in the UK

Dr Judith Bubbear[1], Dr Robin Lachmann[2], Dr Elaine Murphy[2], Dr Gauri Krishna[2], Dr Gavin Clunie[3], Dr Jennifer Walsh[4], Dr Marian Schini[4], Dr Syazrah Salam[4], Dr Matthew Roy[5], Mrs Leigh Mathieson[6], Mrs Victoria Hayes[6], Mr Ben Johnson[6], Mr Daniel Stevens[7], Mr Rakesh Davda[7], Dr Annabel Bowden[8], Dr Mark Nixon[8], Professor Richard Keen[1]

[1]Royal National Orthopaedic Hospital, London, UK. [2]University College London Hospital, London, UK. [3]Cambridge University Hospital Trust, Addenbrookes, UK. [4]Sheffield Teaching Hospitals, Sheffield, UK. [5]University Hospitals, Bristol, UK. [6]Kyowa Kirin International, Marlow, UK. [7]Bionical Emas, Willington, UK. [8]Chilli Consultancy Ltd, Salisbury, UK

### Abstract

Purpose: X-linked hypophosphataemia (XLH; OMIM 307800) is a rare, genetic, progressive, phosphate-wasting disorder that causes lifelong skeletal morbidities, stiffness, pain and impaired physical function. Burosumab inhibits fibroblast growth factor 23, increasing renal phosphate reabsorption. An early access program (EAP) was established in 2019 to provide access to burosumab for adults with XLH in the UK. In this retrospective longitudinal study, medical record data collected during the EAP were used to assess the impact of burosumab on patient-reported outcomes (PROs) in a real-world setting.

Methods: PROs were assessed using the Brief Pain Inventory–short form (BPI-SF), Western Ontario and McMaster Universities Arthritis Index (WOMAC) and EuroQol 5-dimension 5-level (EQ-5D-5L) health survey at baseline and subsequent healthcare visits. Changes from baseline at 6 and 12 months in burosumab-naïve patients were evaluated using paired t-tests and the proportions of patients achieving clinically relevant improvements based on XLH-specific thresholds (Skrinar et al 2019a&b).

Results: Of 142 participants who received at least one dose of burosumab, 136 (96%) were burosumab-naïve at baseline. Their mean (SD) age was 43.4 (14.7) years; 64% were female. Mean (SD) baseline PRO scores in these patients were: BPI-SF Worst Pain 6.9 (2.1; n=101), Pain Severity 5.5 (2.1; n=100), Pain Interference 5.7 (2.5; n=101); WOMAC Stiffness 65.1 (23.9), Physical Function 50.5 (23.8), Pain 51.2 (21.0) and total score 52.0 (22.3) (all n=97); EQ-5D-5L utility 0.51 (0.29; n=108); EQ-VAS 54.2 (20.5; n=103). Significant improvements were seen in all PROs after 6 and 12 months' burosumab treatment (Table 1). After 12 months' burosumab treatment, >50% of patients had clinically relevant improvement in all PROs (where applicable) except BPI-SF Worst Pain.

Conclusions: Burosumab treatment in adults with XLH in real-world practice is associated with clinically meaningful improvements in PROs after 6 months, with improvements maintained at 12 months.

*Table 1 Change in PRO scores from baseline and clinically relevant improvements after 6 and 12 months' burosumab treatment*

| PRO measure | Domain | n at 6/12 months | Change in score from baseline, mean (SD) | | Clinically relevant improvement, n (%) | |
|---|---|---|---|---|---|---|
| | | | 6 months | 12 months | 6 months | 12 months |
| BPI-SF | Worst Pain | 32/33 | −1.8 (2.3) | −1.2 (2.0) | 15 (46.9) | 12 (36.4) |
| | Pain Severity | 32/32 | −1.6 (2.1) | −1.3 (2.0) | No XLH-specific threshold | |
| | Pain Interference | 32/32 | −1.9 (2.2) | −1.5 (2.1) | 19 (59.4) | 22 (68.8) |
| WOMAC | Stiffness | 29/33 | −15.9 (29.7) | −19.7 (24.8) | 16 (55.2) | 21 (63.6) |
| | Physical Function | 29/33 | −15.7 (19.7) | −11.9 (13.9) | 16 (55.2) | 20 (60.6) |
| | Pain | 29/33 | −11.4 (24.3) | −11.1 (16.1) | 18 (62.1) | 17 (51.5) |
| | Total score | 29/33 | −15.4 (18.3) | −12.1 (11.7) | 13 (44.8) | 18 (54.5) |
| EQ-5D-5L | VAS | 31/31 | 17.0 (21.6) | 13.7 (20.5) | No XLH-specific threshold | |
| | Utility | 34/34 | 0.16 (0.23) | 0.17 (0.27) | | |

Decreases in BPI-SF and WOMAC scores indicate improvement
All changes from baseline were significant (p=0.02 for WOMAC Pain at 6 months; p<0.01 for all other outcomes)
BPI-SF, Brief Pain Inventory–short form; EQ-5D-5L, EuroQol 5-dimension 5-Level PRO, patient-reported outcome; SD, standard deviation; VAS, visual analogue scale; WOMAC, Western Ontario and McMaster Universities Arthritis Index; XLH, X-linked hypophosphatemia

Skrinar et al, 2019a. Confirmatory psychometric validation of the Brief Pain Inventory (BPI-SF) in adult X-linked hypophosphatemia (XLH). Value in Health; 22:S870.
Skrinar et al, 2019b. Confirmatory psychometric validation of the Western Ontario McMaster Universities Osteoarthritis inventory (WOMAC) in adult X-linked hypophosphatemia (XLH). Value in Health; 22:S870.

### LB04

### Systematic review of running and risk of osteoarthritis at hip, ankle, hand and lumbar spine

Mr Benjamin Stokes, Professor Katherine Brooke-Wavell

Loughborough University, Loughborough, UK

### Abstract

**Background:** There is lack of consensus regarding whether physical activity is significantly associated with an increased risk of osteoarthritis (OA). Several reviews have examined the association of running – one of the most accessible forms of physical activity – and knee OA, with somewhat inconsistent findings, but there are few syntheses of associations of running with OA at other joints. Therefore, we aimed to systematically review research relating running and risk of OA at other joints.

**Methods:** The review was conducted according to PRISMA guidelines. Six databases were searched for relevant articles. For studies to be included, the study exposure had to be a self-reported assessment of running status, with a primary outcome of OA diagnosis. Included studies were assessed for risk of bias using the JBI Checklist for Cohort Studies.

**Results:** Twelve studies were identified. All were conducted in North America, Europe or Australia. The majority (nine) were of prospective cohort design, with two cross-sectional and one case-control comparisons. Only three studies were categorised as high quality. Ten studies examined hip OA: two reported incidence/prevalence to be lower with higher running exposure; three reported prevalence to be higher; three reported no significant association and one found higher incidence in male runners aged under 50 but not older men or women. Hand, lumbar spine and ankle OA were each examined in a single study, with none of these showing significant associations.

**Conclusion:** This systematic review suggests inconsistent findings on associations between running exposure and hip OA, with little evidence on OA at the hand, lumbar spine and ankle. Inconsistent findings may be explained by methodological differences and limitations. Given the public health importance of physical activity and popularity and accessibility of running, further high quality research is needed.

### LB05

### Craniofacial defects with dual TNAP and PHOSPHO1 deletion in a murine model

Dr Lucie Bourne[1], Dr Lucinda Evans[2,1], Dr Sonoko Narisawa[3], Mr Zain Ghani[1], Miss Humayra Iqbal[1], Dr Louise Stephen[4], Prof. José Luis Millán[3], Prof. Brian Foster[5], Prof. Colin Farquharson[4], Prof. Katherine Staines[1]

[1]University of Brighton, Brighton, UK. [2]Royal Veterinary College, London, UK. [3]Sanford Burnham Prebys Medical Discovery Institute, San Diego, USA. [4]The Roslin Institute and Royal (Dick) School of Veterinary Studies, University of Edinburgh, Edinburgh, United Kingdom. [5]The Ohio State University, Columbus, USA

### Abstract

Biomineralisation requires phosphatases for inorganic phosphate availability, yet it is unknown how TNAP and PHOSPHO1 coordinate their functions. We have previously generated mice with a conditional *Prx1*-specific deletion of TNAP on a global *Phospho1*$^{-/-}$ genetic background [(*Alpl*$^{fl/fl}$;*Prx1-Cre*$^{+/-}$;*Phospho1*$^{-/-}$), aka dKO] which exhibit an altered long bone skeletal phenotype. Herein, we examined their craniofacial phenotype to understand the dual role of TNAP and PHOSPHO1 in molar, mandibular and skull morphogenesis.

MicroCT analysis revealed that the dual deletion of *Alpl* and *Phospho1* in 1-day-old mice resulted in reduced bone surface area of the entire skull ($p<0.0001$) compared to WT and *Alpl*$^{fl/fl}$;*Prx1-Cre*$^{+/-}$ (*Alpl* cKO) mice. Similarly, global deletion of just *Phospho1* (*Phospho1*$^{-/-}$) and the single allele deletion of *Alpl* on this background (*Alpl cKO-het*;*Phospho1*$^{-/-}$) resulted in reduced bone area ($p<0.0001$). Interorbital distance and skull height were reduced in *Phospho1*$^{-/-}$ animals compared to all other genotypes ($p<0.05$), whilst mandible height was altered in *Phospho1*$^{-/-}$, *Alpl cKO-het*;*Phospho1*$^{-/-}$ and dKO mice ($p<0.05$).

At 25 days, cranial bone surface area was significantly reduced in the dKO animals compared to all other genotypes ($p<0.05$). In addition, mandibular measurements (height, plain, condylar axis, anterior and posterior length), nasal bone, frontal and skull lengths, were significantly reduced in these mice compared to the other genotypes ($p<0.05$). *Phospho1*$^{-/-}$ and *Alpl cKO-het;Phospho1*$^{-/-}$ mice were also detrimentally altered compared to WT and *Alpl cKO* in these parameters ($p<0.05$). Measurements of mandibular molar 1 (M1) at 25 days demonstrated a significant reduction in dentin volume ($p<0.01$) and thickness ($p<0.05$) in the dKO mice, yet the other genotypes were unaffected.

To align with humane practices in animal research, dKO animals were not maintained beyond 25 days. However, analysis of the other genotypes at 42 days, in males and females, showed cranial bone surface area was unchanged. Inner canthal distance and mandibular plain were reduced in female *Phospho1*$^{-/-}$ and *Alpl cKO-het;Phospho1*$^{-/-}$ mice ($p<0.05$), yet other parameters were unaltered, and males were largely unaffected. M1 analysis revealed limited differences in male and female mice between genotypes.

Data here suggests that mice with dual deletion of TNAP and PHOSPHO1 exhibit a severe brachycephalic craniofacial skeletal phenotype, with reduced bone surface and concurrent defects in their dental development. Ongoing characterisation of these mice will enable further delineation of this interaction.
